# 2-Acetyl-5,8-dihydro-6-(4-methyl-3-pentenyl)-1,4-naphthohydroquinone-Derived Chalcones as Potential Anticancer Agents

**DOI:** 10.3390/molecules28207172

**Published:** 2023-10-19

**Authors:** Javier Maldonado, Alfonso Oliva, Aurora Molinari, Waldo Acevedo

**Affiliations:** Instituto de Química, Facultad de Ciencias, Pontificia Universidad Católica de Valparaíso, Valparaíso 23732223, Chile; javier.maldonado@pucv.cl (J.M.); alfonso.oliva@pucv.cl (A.O.)

**Keywords:** chalcone-1,4-naphthoquinone/benzohydroquinone, antiproliferative activity, molecular docking

## Abstract

Based on previous results with benzoindazolequinone (BIZQ) and 3-methylnaphtho [2,3-d]isoxazole-4,9-quinone (NIQ) derivatives, a novel series of chalcone-1,4-naphthoquinone/benzohydroquinone (CNQ and CBHQ) compounds were synthesized from 2-acetyl-5,8-dihydro-6-(4-methyl-3-pentenyl)-1,4-naphthohydroquinone. Their structures were elucidated via spectroscopy. These hybrids were assessed in vivo for their antiproliferative activity on MCF-7 breast adenocarcinoma and HT-29 colorectal carcinoma cells, revealing cytotoxicity with IC_50_ values between 6.0 and 110.5 µM. CBHQ hybrids **5e** and **5f** displayed enhanced cytotoxicity against both cell lines, whereas CNQ hybrids **6a**–**c** and **6e** exhibited higher cytotoxic activity against MCF-7 cells. Docking studies showed strong binding energies (ΔG*_bin_*) of CNQs to kinase proteins involved in carcinogenic pathways. Furthermore, our in silico analysis of drug absorption, distribution, metabolism, and excretion (ADME) properties suggests their potential as candidates for cancer pre-clinical assays.

## 1. Introduction

The uncontrolled division and spread of abnormal cells remain a global medical challenge, with cancer being a leading cause of death [1,2]. Of the 9.9 million new cancer cases diagnosed, the most common are breast (11.7%), lung (11.4%), colorectal (10.0%), prostate (7.3%), and stomach cancer (5.6%) [3].

Quinones are widely studied compounds for their interesting biological activities [4,5,6], with daunorubicin and doxorubicin being key pillars in oncology treatments [7]. Their antitumor properties are primarily due to the inhibition of DNA topoisomerases through intercalation and the generation of reactive oxygen species (ROS), which contribute to cellular oxidative stress [8,9]. Naphthoquinones/hydroquinones regulate antiproliferative processes through p53, kinases, vascular endothelial growth factor receptor 2, COX-2, protein activators of transcription (STAT3), the inhibition of signaling via EGFR-NF-kB, and the inhibition of protein phosphatase Cdc25, among others [10,11,12,13,14].

Chalcones, 1,3-diaryl-2-propen-1-ones, are precursors to flavonoids, flavanones, and aurones [15,16,17] with diverse pharmacological benefits, including antineoplastic, antioxidant, and anti-inflammatory properties [18,19]. Studies of their antineoplastic properties have revealed interactions with various target proteins involved in proliferation [20,21,22]. These compounds disrupt the cell cycle by interfering with microtubules, which play critical roles in mitosis, motility, and transport. Their binding to tubulins prevents polymerization, altering mitotic spindle assembly and disrupting cytoskeletal function, leading to cell cycle arrest in the G2/M phase [23,24].

Chalcones also activate the p53 protein, regulating the cell cycle by binding to the human oncoprotein Mdm2 (murine double minute 2). They inhibit angiogenesis by blocking vascular endothelial growth factor (VEGF) and various enzymes, including topoisomerases, the androgen receptor, mitogen-activated protein kinases (ERK1/2), cyclooxygenase 2 (COX-2), and histone deacetylase (HDACs), ultimately inducing apoptosis [25,26,27]. The presence of a conjugated double bond with the carbonyl group is responsible for these biological properties due to its structural planarity, making this compound family a crucial pharmacophore for synthesizing new antitumor molecules [28].

**Figure 1:**
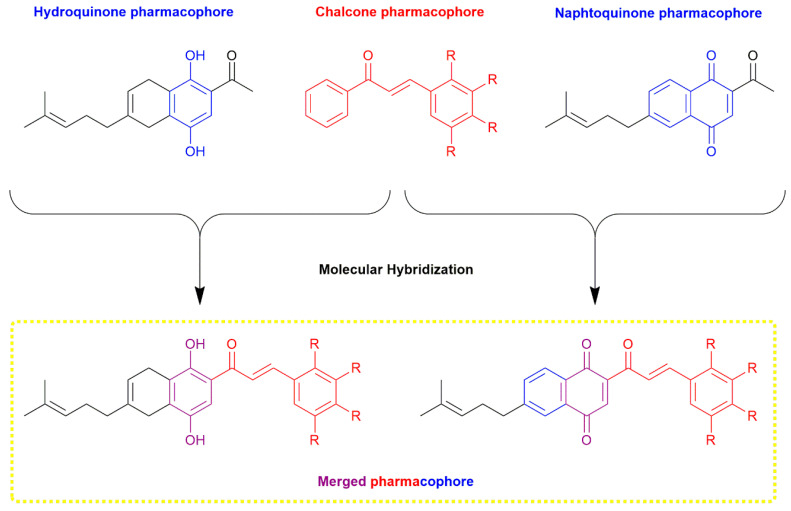
Rational design of novel chalcone-naphthoquinone/hydroquinone hybrids.

In the development of new anticancer drugs, molecular hybridization is a promising strategy, involving the rational design of compounds that combine multiple pharmacophores within a single structure to enhance antiproliferative activities [29,30,31]. Chalcone hybrids have shown significant antineoplastic potential against various cancer types [32,33,34,35], while quinone-based hybrids exhibit high cytotoxicity [10,36,37]. Our research group specializes in designing and synthesizing hybrid molecules by combining 1,4-naphthoquinones with azoles, demonstrating their significant anticancer properties against breast cancer (MCF-7) and gastric cancer (KATO-III) [38,39,40]. In this work, we propose the rational design, synthesis, and in vitro anticancer evaluation of novel chalcone-naphthoquinone/hydroquinone hybrids (Figure 1). For this purpose, we use 2-acetyl-5,8-dihydro-6-(4-methyl-3-pentenyl)-1,4-naphthohydroquinone as a precursor, since it was developed in our previous studies. This compound proved to be a pharmacophore that generated structures with promising in vitro antitumor activity against neoplastic cell cultures [41]. Additionally, we conduct molecular docking studies with cancer-related proteins and in silico ADME predictions to explore potential mechanisms of anticancer action and the drug properties of these newly synthesized hybrids.

## 2. Results and Discussion

### 2.1. Chemistry

**Scheme 1:**
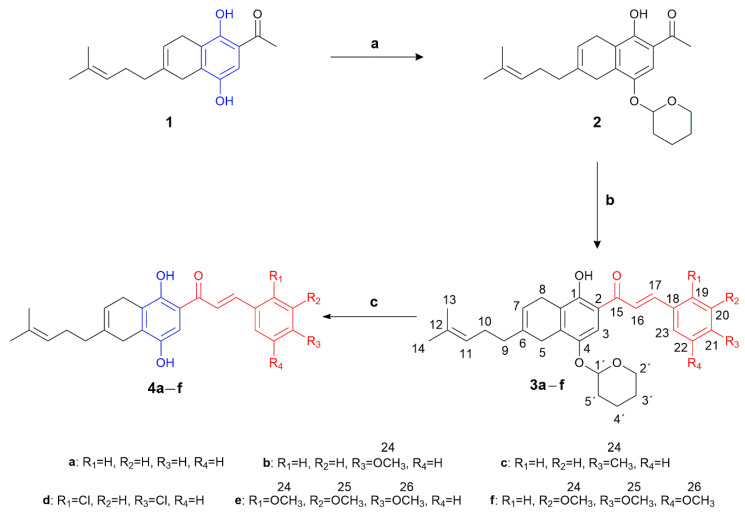
Synthesis route of compounds **4a**–**f**. Reagents and conditions: (**a**) DHP, PPTS, CH_2_Cl_2_, rt, 19 h. (**b**) Ba(OH)_2_·8H_2_O, benzaldehydes, EtOH, 90 °C, 25 min; (**c**) PTSA, MeOH, rt, 3 h.

As shown in Scheme 1, the synthesis of compounds **2**–**4a**–**f** was performed from the precursor 2-acetyl-5,8-dihydro-6-(4-methyl-3-pentenyl)-1,4-naphthohydroquinone **1**, following previous experimental methods [41,42]. Initially, it was necessary to protect the hydroxyl group attached to C-4 of the precursor to eliminate the acidic properties attributed to the phenolic 4-OH group. This protection was achieved using 3,4-dihydro-2H-pyran (DHP) as a protective agent, in the presence of p- pyridinium toluenesulfonate (PPTS), yielding 80%. The phenolic 1-OH group, on the other hand, remained unaltered due to its stabilization through intramolecular hydrogen bonding with the adjacent carbonyl group, forming a stable six-membered cyclic system [43].

The synthesis of the new chalcone-1,4-benzohydroquinone (CBHQ) **4a**–**f** hybrids involved Claisen–Schmidt condensation of the protected derivative **2** (as tetrahydropyranyl ether, 4-OTHP) with the respective benzaldehyde in the presence of barium hydroxide octahydrate [44]. By using an ace-pressure tube to carry out the reaction mixture, it was possible to increase the synthesis yields of these products and significantly reduce the reaction time compared to the traditional use of reflux heating. This process yielded chalcones **3a**–**f** with moderate yields (60–70%). The subsequent deprotection of the THP group by acid hydrolysis using 4-toluenesulfonic acid monohydrate (PTSA) was carried out with good yields (93–96%).

The structures of derivatives **2**–**4a**–**f**, along with other newly synthesized compounds, were elucidated spectroscopically by IR, ^1^H NMR, ^13^C NMR, and elemental analyses. In the infrared (IR) spectra, the characteristic stretching vibration (stv) absorption bands of phenolic O-H bonds were observed in the range of 3272–3492 cm^−1^, in addition to the *stv* bands of C=O bonds at 1630–1662 cm^−1^. The *stv* band of the C=C bond in the α,β-unsaturated fragment of chalcones was observed at 1558–1633 cm^−1^.

In the ^1^H NMR spectrum of derivative **2**, signals corresponding to the protons in the methylene groups of the tetrahydropyranyl protecting ring (H-3′, H-4′, H-2′, and H-5′) were observed between δ 1.66 and 3.90 ppm. The signal for the methine group H-1′ was observed at δ 5.33 ppm. Additionally, the methyl group H-16 appeared at δ 2.58 ppm, while the phenolic proton 1-OH exhibited a higher chemical shift (δ 12.45 ppm). The ^1^H NMR and ^13^C NMR spectra of compound **2** are shown in Figure S1 and Figure S2, respectively.

The ^1^H NMR spectra of derivatives **3a**–**f** showed signals belonging to the protons of the α,β-unsaturated fragment characteristic of chalcones H-16 and H-17 in the ranges of δ 7.46–7.69 and δ 7.90–8.20 ppm, respectively. The signals of the analogous protons H-16 and H-17 of hybrids **4a**–**f** were observed at δ 7.67–7.88 and δ 7.84–8.12 ppm, respectively, with both coupled protons of the unsaturated system having trans geometry (J 15, 3–16.0 Hz). Most of the phenolic protons at 4-OH and 1-OH appeared as singlets in the ranges of δ 7.94–8.12 and 12.78–13.08 ppm, respectively. Notably, in the ^1^H NMR spectra of hybrids **4b**, **4e,** and **4f**, methoxyl singlets were observed between δ 3.81 and 3.98 ppm, and the signal corresponding to the methyl group H-24 of compound **4c** appeared at δ 2.38 ppm. The ^1^H NMR spectra of compounds **3a**–**f** and **4a**–**f** are shown in Figures S3–S14.

In the ^13^C NMR spectra of compounds **3a**–**f** and **4a**–**f**, the characteristic carbonyl of chalcones C-15 is in the range of δ 192.7–193.6 and δ 192.7–193.4 ppm, respectively. For compounds **3a**–**f**, the presence of the signals corresponding to C-4 and C-1 occurred between δ 144.8 and 146.4 and δ 156.8 and 157.0 ppm, respectively. The signals of the analogous carbons C-4 and C-1 of the 1,4-benzohydroquinone fragment of hybrids **4a**–**f** were observed at δ 144.9–146.4 and δ 155.2–156.5 ppm, respectively. The ^13^C NMR spectrum of compounds **3a**–**f** and **4a**–**f** are shown in Figures S15–S26.

**Scheme 2:**
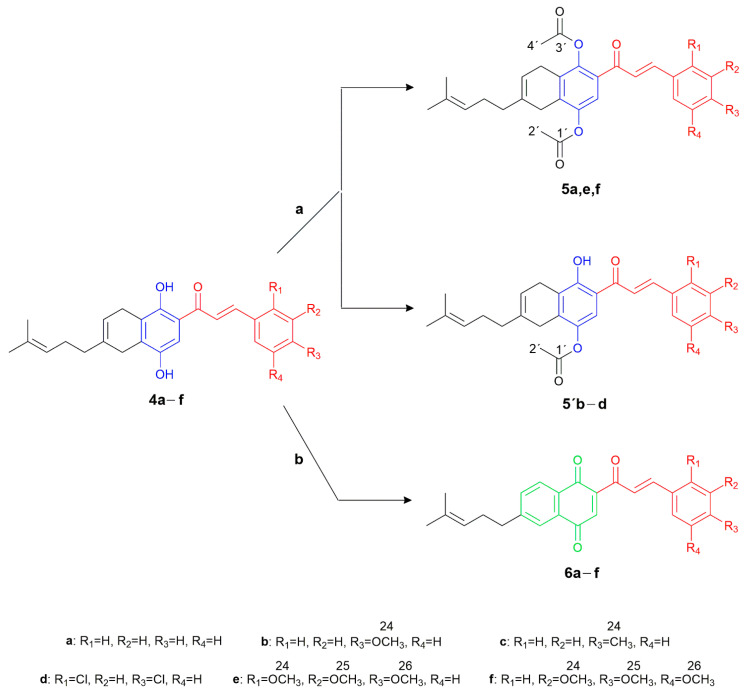
Synthesis of hybrid molecules **5a**,**e**,**f**, **5′b**–**d,** and **6a**–**f**. Reagents and conditions: (**a**) acetic anhydride, pyridine, rt, 24 h. (**b**) DDQ, CH_2_CH_2_, rt, 30 min.

The acetylation of the phenolic groups in the CBHQ **4a**–**f** hybrids was carried out using acetic anhydride in pyridine [45]. As depicted in Scheme 2, this process yielded 1,4-diacetylated derivatives **5e** and **5f**, as well as 4-monoacetylated derivatives **5′b**, **5′c**, and **5′d**. The formation of these derivatives resulted from the stabilization of the proton through intramolecular hydrogen bonding with the adjacent carbonyl group. These compounds were synthesized with favorable yields ranging from 85% to 95%, except for derivative **5a**, which could not be obtained in pure form.

Furthermore, the obtained compounds **4a**–**e** facilitated the synthesis of a new series of molecular hybrids, denoted as chalcones-1,4-naphthoquinones (CNQ) **6a**–**f** (Scheme 2). These hybrids were generated through in situ reactions involving the oxidation of carbons C-1 and C-4 in the phenolic groups, along with the aromatization of the ring fused to the 1,4-benzohydroquinone moiety, using an excess of DDQ in CH_2_Cl_2_ at room temperature [39]. The new hybrids **6a**–**e** were obtained with moderate yields ranging from 30% to 58%, while the derivative **6f** could not be adequately purified.

In the infrared (IR) spectra of compounds **5e** and **5f**, characteristic stretching vibration (*stv*) absorption bands of the C=O groups in the acetyl units were observed at 1766 cm^−1^. On the other hand, derivatives **5′b**, **5′c**, and **5′d** exhibited *stv* bands at 3379–3391 cm^−1^ and 1755–1758 cm^−1^, corresponding to the phenolic O-H bond and the C=O bond of the acetyl group, respectively. For hybrids **6a**–**e**, the *stv* band related to the C=O group of the quinone moiety was observed in the range of 1654–1668 cm^−1^.

In the ^1^H NMR spectra of derivatives **5e** and **5f**, signals corresponding to the protons within the acetyl group (H-2′ and H-4′) were detected between δ 2.29 and 2.33 and δ 2.33 and 2.37 ppm, respectively. Additionally, the signal for the methyl group H-2′ in derivatives **5′b**, **5′c**, and **5′d** appeared at δ 2.38–2.41 ppm, while the phenolic proton 1-OH manifested as a singlet in the range of δ 13.05–13.32 ppm. Concerning hybrids **6a**–**e**, the signals attributed to the aromatic protons of the naphthoquinone moiety (H-7, H-5, and H-8) were observed in the ranges of δ 7.47–7.64, δ 7.66–7.94, and δ 8.00–8.07 ppm, respectively. The ^1^H NMR spectrum of compounds **5′b**–**f** and **6a**–**e** are shown in Figures S27–S36.

In the ^13^C NMR spectra of compounds **5e** and **5f**, intense signals corresponding to both carbonyl groups of the acetyl units (C-1′ and C-3′) were evident at δ 169.0 ppm. For the derivatives **5′b**, **5′c**, and **5′d**, the signal of C-1′ appeared between δ 169.8 and 169.9 ppm. In the case of hybrids **6a**–**e**, signals attributed to the carbonyl groups of the quinone system (C-4 and C-1) were observed in the ranges of δ 183.4–183.5 ppm and δ 185.1–185.4 ppm, respectively. The ^13^C NMR spectra of compounds **5′b**–**f** and **6a**–**e** are shown in Figures S37–S46.

### 2.2. In Vitro Cytotoxicity Assays

The antiproliferative activity of the new derivatives **4a**–**f**, **5e**–**f**, **5′b**–**d**, and **6a**–**e** was evaluated on MCF-7 and HT-29 cancer cell lines using a CellTiter 96^®^ AQueous One Solution Cell Proliferation Assay (MTS). The results, presented in Table 1, are expressed as the half-maximal inhibitory concentration (IC_50_ ± standard deviation) for cell proliferation inhibition, along with the corresponding -log_10_ values (pIC_50_).

Overall, the compounds exhibiting noteworthy antineoplastic activity in this study demonstrated a higher cytotoxic effect against MCF-7 breast carcinoma, with pIC_50_ values ranging from 4.07 to 5.09. Similar results were observed for HT-29 colon cancer, with pIC_50_ values ranging from 3.74 to 5.22. Notably, the derivatives **5e**, **5f**, and **6b** exhibited outstanding cytotoxicity against MCF-7, while the compounds **5e** and **5f** demonstrated significant activity against the HT-29 cell line. Conversely, the series of CBHQ hybrids (**4a**–**f**) and the 4-monoacetylated CBHQ derivatives (**5′b**–**d**) did not exhibit significant cytotoxic activity in either cancer cell cultures (pIC_50_ < 3.52).

Regarding the HT-29 cell line, an analysis of cytotoxicity based on the type of substituent in the aromatic ring of the chalcone fragment revealed that the methoxyl groups enhanced antiproliferative effects. An increase in the number of these substituents corresponded to a marked increase in cytotoxicity, with the derivatives **5e** and **5f** displaying high pIC50 values of 4.87 and 5.22, respectively.

A similar trend was observed against the MCF-7 cell line, in which the trimethoxylated derivatives **5e** and **5f** achieved pIC_50_ values of 4.96 and 5.09, respectively. In contrast, the presence of 2,4-dichloro substituents in compound **6d** resulted in limited cytotoxic effects, with pIC_50_ values lower than 3.52 against both neoplastic cultures.

Regarding the in vitro cytotoxicity data (Table 1), the majority of the compounds in the CNQ hybrid series exhibited superior pIC_50_ values compared to those of CBHQs **4a**–**f** and the monoa-cetylated **5′a**–**d** hybrid series. This highlights the significance of the naphthoquinone and 1,4-diacetylated benzohydroquinone pharmacophores present in these structures in terms of their cytotoxic activity against both assessed carcinogenic cell lines.

**Figure 2:**
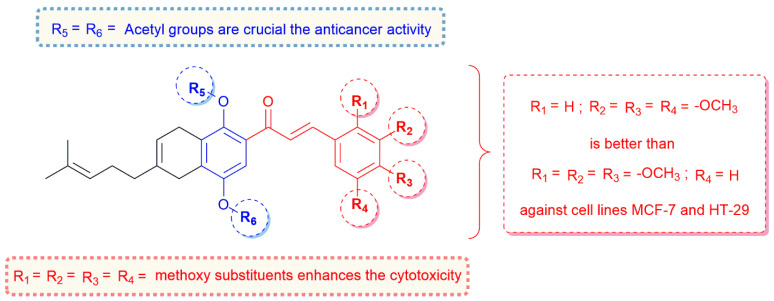
SAR of chalcone–hydroquinone hybrids as anticancer agents.

In terms of the structure–activity relationship, these findings underscore the exceptional cytotoxicity of the trimethoxylated 1,4-diacetylated CBHQ compounds **5e** and **5f** against both cancer lines. Compound **5f**, in particular, displayed the highest potency against the HT-29 line (pIC_50_ 5.22), comparable to that of the reference drug doxorubicin (pIC_50_ 5.39). This underscores the pivotal role played by the -OCH_3_ and 1,4-COCH_3_ substituents, which significantly influence the cytotoxicity exhibited by CBHQs **5e**–**f** derivatives (Figure 2). These results align with those of previous reports identifying various methoxychalcones with anti-tumor properties, attributed to the electron-donating properties of the aryl ring of chalcones. The presence of the methoxy group enhances the anticancer activity of these structures [46,47,48].

Furthermore, CNQs **6a**–**f** generally demonstrated superior pIC_50_ values compared to those of benzohydroquinone CBHQs **4a**–**f** and the monoacetylated benzohydroquinone compounds **5b**–**d**. Hence, the diacetylation of both hydroxyl groups and the presence of the quinone ring also play significant roles in their cytotoxic activity. These compounds hold promise for exploring their cytotoxic potential against other malignant cell lines. Indeed, research is evolving towards optimizing these compounds to develop lead molecules for potential antitumor drugs. This includes incorporating -OCH_3_ in different positions of the aryl group of diacetylated chalcones and acetylating both hydroxyl groups.

It is worth noting that the mechanism of action for doxorubicin, the reference drug in Table 1, involves the inhibition of DNA topoisomerases I and II. Doxorubicin stabilizes the tertiary adduct (DNA-drug-Topo), which prevents DNA strand rewinding, disrupts the cell cycle equilibrium, and ultimately induces apoptosis [38,49]. Since the tested compounds in this study contain a quinone system, they may potentially share a similar mechanism of action with doxorubicin. However, these compounds did not exhibit strong binding energies (ΔG*_bin_*) for topoisomerases compared to that of doxorubicin (Table S1). Therefore, in this study, we aimed to explore alternative mechanisms for the antineoplastic action of these cytotoxic hybrids through molecular-docking-based virtual screening with various cancer-related proteins, including growth factor receptors, transcription regulators, and enzymes (such as reductases, oxidases, and kinases).

### 2.3. In Silico Virtual Screening for Potential Antineoplastic Targets of Synthesized Cytotoxic Hybrids

Considering the higher cytotoxicity of the compounds (with pIC_50_ values greater than 4, as shown in Table 1), we conducted in silico molecular docking studies to identify potential biological targets for these new antiproliferative hybrids, providing insights into their possible mechanisms of action. To achieve this, we predicted the potential docking sites of these cytotoxic hybrids in several cancer-related proteins and calculated their corresponding ΔG*_bin_* values. For robust results, we focused on a subset of cancer-related proteins with known 3D structures, conducting independent searches with the compounds and utilizing their most stable conformers during interactions with these biological targets. The selected proteins are known to be overexpressed in breast cancer cells, including epidermal growth factor receptor (EGFR), epidermal growth factor receptor 2 (HER2), mesenchymal-epithelial transition factor (c-MET), tropomyosin receptor kinase A (TRKA), mitogen-activated protein kinases (MAPK1, ERK2, MEK1), tyrosine protein kinase (TPK), dihydrofolate reductase (DHFR), fibroblast growth factor receptor 2 (FGFR-2), vascular endothelial growth factor receptor 2 (VEGFR-2), estrogen receptors (ERs), cyclooxygenase (COX-2), and tubulin (TUB), among others. Additionally, COX-2 and MEK1 are overexpressed in colon adenocarcinoma cell lines [50,51,52,53,54,55,56,57].

The results of the virtual screening, encompassing all the mentioned proteins, indicated that the majority of the synthesized cytotoxic compounds exhibit a stronger binding affinity for kinase proteins. As shown in Table S1, when considering only kinase proteins (Table 2), it is evident that most of these compounds bind more strongly to c-MET, a receptor tyrosine kinase, with ΔG*_bin_* values ranging from −10.6 to −9.7 (with an average of −10.08) kcal/mol. This is followed by TRKA, with binding energies ranging from −11.0 to −8.9 (with an average of −9.97) kcal/mol. Interestingly, despite the higher cytotoxicity of CBHQ hybrids **5e** and **5f**, naphthoquinone derivatives **6a**–**c** and **6e** demonstrated a greater affinity for most of the studied proteins, with ΔG*_bin_* values surpassing those of the diacetylated benzohydroquinone derivative CBHQs. Among them, compound **6c** displayed the most favorable ΔG*_bin_* values for several kinase proteins, including TRKA (−11.0 kcal/mol), c-MET (−10.6 kcal/mol), and TPK (−10.4 kcal/mol), as detailed in Table 2. These findings underscore the significance of the naphthoquinone planar system in CNQ derivatives **6**, both in their affinity for cancer-related proteins and their cytotoxicity. In contrast, monoacetylated or diacetylated derivatives **5** exhibited lower binding energies due to their reduced potential for strong hydrogen bond interactions with amino acid residues, as they protect one or both of the hydroquinone hydroxyl groups.

While CBHQ derivatives **4** did not exhibit cytotoxicity against the tested cancer cell lines (Table 1), they demonstrated favorable binding energies within the active sites of kinase proteins, as presented in Table S2. This observation can be attributed to robust hydrogen bond interactions between the free hydroxyl groups of the hydroquinone system and oxygen- or nitrogen-containing groups within the proteins, as illustrated in Figures S47–S49.

Despite previous studies suggesting chalcone derivatives as tubulin polymerization inhibitors, the evaluated compounds did not consistently yield the best ΔG*_bin_* average values in comparison to those of other proteins, including c-MET and TRKA, among others (Table S1). Furthermore, it is worth noting that most of the compounds exhibited superior ΔG*_bin_* values compared to reference antiproliferative drugs such as erlotinib, larotrectinib, almonertinib, and anlotinib, all of which function as kinase inhibitors. Erlotinib, almonertinib, and larotrectininb are utilized for treating non-small cell lung cancer (NSCLC), while anlotinib is employed for various cancers, including NSCLC and different sarcoma types [58,59,60].

### 2.4. Binding Site and Docking of Synthesized Cytotoxic Hybrids in c-MET, TRKA, and HER2 Targets

As previously mentioned, the virtual screening results indicated that the majority of the cytotoxic hybrids exhibit a high affinity for target proteins, with an average ΔG*_bin_* of less than −8.6 kcal/mol. In general, these compounds displayed stronger binding to the c-MET receptor (with an average of −10.08 kcal/mol), followed by TRKA (with an average of −8.96 kcal/mol) and HER2 (with an average of −9.75 kcal/mol). These findings suggest that these hybrids might serve as potent inhibitors of c-MET, TRKA, and HER2, all of which are overexpressed in certain types of cancer, including human breast and colorectal cancer [55,61,62,63]. Thus, these synthesized hybrids hold promise for treating diseases driven by these enzymes and could be effective against proliferative disorders.

**Figure 3:**
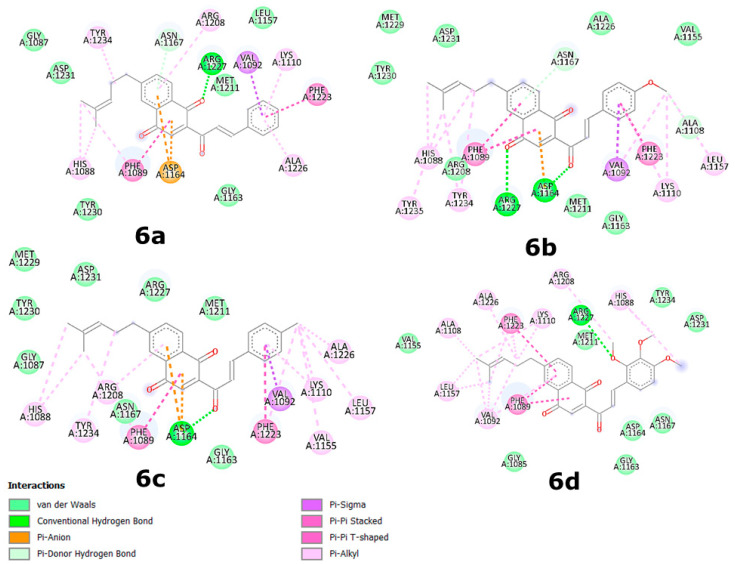
Plotted 2D maps of H-bonds and hydrophobic interactions of CNQ **6a**, **6b**, **6c,** and **6d** with c-MET residues. Van der Waals, Pi–Anion, Pi–Sigma, Pi–Pi stacked, and Pi–alkyl are considered hydrophobic interactions.

**Figure 4:**
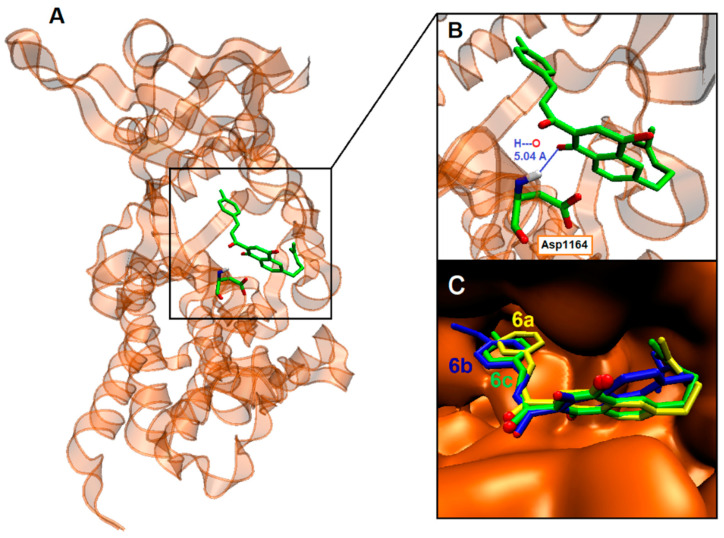
(**A**): Visualization of the potential binding site of the CNQ hybrid **6c** into c-MET; (**B**): Detail of its H-bonding with Asp1164; (**C**): Superimposition of the docking poses for CNQ hybrids **6a** (yellow), **6b** (blue), and **6c** (green).

Detailed configurations of the binding sites, along with the amino acids involved in the docking of synthesized cytotoxic hybrids and their corresponding ΔG*_bin_* values for c-MET, TRKA, and HER2, are presented in Table 3 and depicted in 2D maps in Figure 3. Additionally, 3D docking complexes of c-MET with **6a**, **6b**, and **6c** are illustrated in Figure 4. Complementary 2D maps for complexes involving **5e** and **5f** can be found in Figure S1, and binding site interactions of the synthesized cytotoxic hybrids with amino acids of MEK-1, TPK, and EGFR are outlined in Table S3.

Overall, as demonstrated in Table 2 and Table 3, CNQ derivatives **6** exhibited superior binding affinities for kinase proteins due to the presence of the C1 and C4 carbonyl groups within the quinone ring. These groups interacted with amino acid residues through hydrogen bonding. For instance, carbonyl groups from the quinone ring in derivatives **6a** and **6b** interacted with Arg1127 of c-MET (Figure 3). Additionally, these interactions were favored due to the greater planarity of naphthoquinone structures compared to that of benzohydroquinone structures. Specifically, CNQs **6a**, **6b**, and **6c** displayed excellent binding affinities for c-MET, with ΔG*_bin_* values of −10.3, −10.3, and −10.6 kcal/mol, respectively. Peptide sequences surrounding the CNQs revealed consistent docking in the same region of the enzyme, defined by the residues Arg1208 and Asp1231. All the compounds, including **5e** and **5f**, engaged in hydrogen bonding with c-MET residues, as well as various other interactions, including Van der Waals, Pi–Anion, Pi–Sigma, Pi–Pi stacked, and Pi–alkyl interactions.

Regarding hydrogen bonds, the residues Arg1208 and Asp1231 were most commonly involved in interactions with c-MET, serving as hydrogen bond donors toward carbonyl groups from the quinone moiety of **6a** and **6b**, the chalcone moiety of **6c** and **6e**, or the methoxy group of **5e** and **5f** (Figure 3 and Figure S1). In the case of TRKA, only the residues Ser672 and Arg673 interacted with carbonyl groups from the quinone and chalcone moieties of **6e**, while Met592 interacted with the carbonyl group from the methoxy group of **5f** through hydrogen bonds (Figure S2). HER2 exhibited interactions with residues such as Thr798, which primarily engaged in hydrogen bonds with carbonyl groups from the quinone and chalcone moieties of **6a**, **6b**, and **6e**, as well as Ser783, which interacted with the carbonyl groups from the chalcone moiety of **6a** and **6e** and the methoxy group of **5e** (Figure S3).

Aromatic interactions, similar to hydrogen bonds, play a crucial role in ligand–protein interfaces. Many contemporary ligand docking programs implicitly account for aromatic stacking through van der Waals and Coulombic potentials [64]. Residues Phe1089 and Phe1223 were notably involved in these interactions, engaging in π–π stacking with the aromatic rings of naphthoquinone systems in **6a**–**c/6d** and the chalcone system in **5e** and **5f**. Additionally, the aromatic ring of Phe1223 interacted with the aromatic rings of the chalcone moiety in **6a**–**c** through π–π stacking and with carbons of the hydroquinone system in **5e** and **5f** through π–alkyl interactions (Figure 3 and Figure S1).

In the case of TRKA, Phe669 was the primary residue involved in aromatic interactions, participating in π–π stacking with the aromatic rings of the chalcone moiety in **5e** and **5f** and the naphthoquinone moiety in **6a**–**c** and **6e**. Val524 interacted through π–sigma interactions with the aromatic rings of the quinone moiety in **5f**, **6a**, **6b**, and **6c** (Figure S2). Lastly, Phe864 played a prominent role in HER2 interactions, engaging in π–π stacking with the aromatic rings of the chalcone moiety in **5f** and the naphthoquinone moiety in **6b**, **6c**, and **6e**. Leu852 also contributed through π–sigma interactions with the aromatic rings of the chalcone moiety in **5e**, **6b**, **6c**, and **6e**, as well as the naphthoquinone moiety in **6a** (Figure S3).

To validate the binding sites of the synthesized cytotoxic hybrids within the kinases, we conducted a comparative analysis of CNQ **6c** complexes with those of known kinase ligands. The results revealed that the binding regions of CNQ **6c** indeed overlap with the catalytic sites of the target enzymes, sharing a common set of contacts with the respective inhibitors (Table 4). Notably, the active site residues involved in these interactions include Phe1089, Val1092, Lys1110, Leu1157, Gly1163, Met1211, Ala1226, and Arg1227 for c-MET, Val524, Ala542, Kys544, Glu560, Val573, Phe589, Leu657, Gly667, Asp668, and Phe669 for TRKA, and Leu726, Val734, Ala751, Kys753, Leu785, Leu796, Thr798, Gln799, Leu800, Met801, Gly804, Leu852, Thr862, Asp863, and Phe864 for HER2. These residues served as common contact points for CNQ **6c** and ligands 1, 2, and 3 in all three enzymes, respectively.

**Figure 5:**
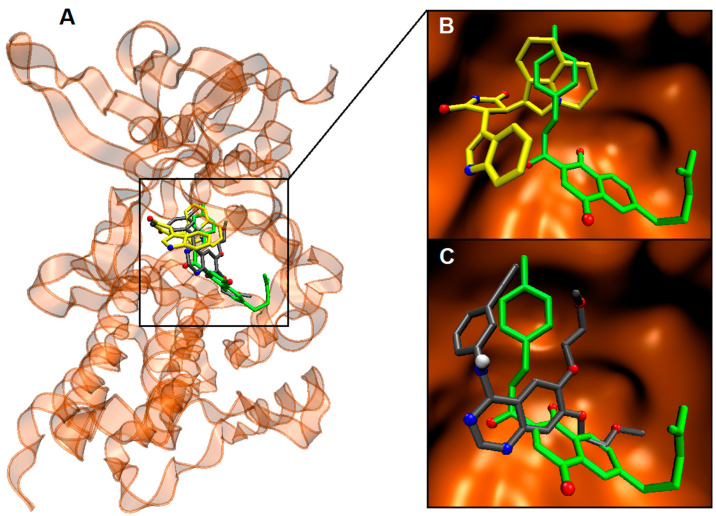
(**A**): Overlapping of the docking poses for CNQ hybrid **6c** (green), ligand 1 (yellow), and erlotinib (grey) into c-MET. Superimposition of the docking poses for (**B**): **6c** and ligand 1 as well as (**C**): **6c** and erlotinib.

Of particular interest is the observation that the energetic aspects of these interactions favored CNQ **6c** in comparison to erlotinib, with a favorable energy difference of 1.5 kcal/mol for c-MET and 2.2 kcal/mol for HER2. Moreover, **6c** exhibited the same in silico affinity as larotrectinib for TRKA, both achieving a ΔG*_bi__n_* value of −11.0 kcal/mol. Importantly, the aromatic ring within the chalcone moiety of CNQs plays a pivotal role in these interactions, directly contributing to the overlap with the ligands at the catalytic sites of the enzymes (Figure 5). This crucial involvement of the chalcone moiety is consistently observed in the case of TRKA and HER2 as well (Figures S50 and S51).

**Figure 6:**
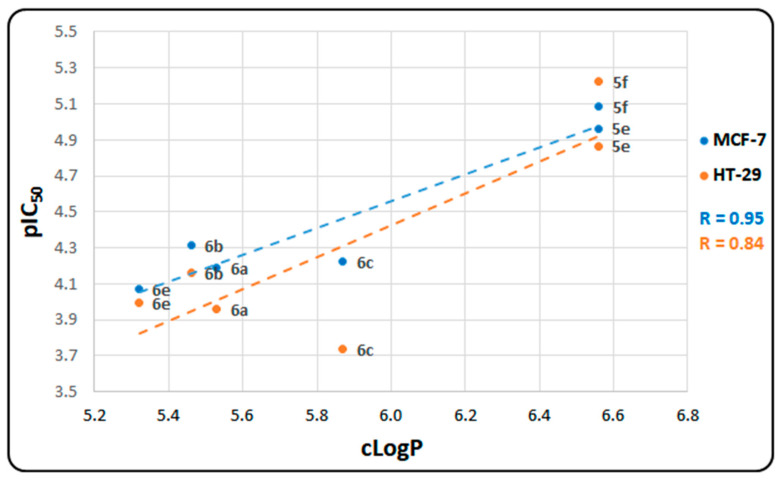
Relationship between pIC_50_ and cLogP values for synthesized cytotoxic hybrids.

To strengthen our research, it is important to identify and analyze the correlations between our calculated properties and experimental results. In this regard, we examined the correlation between experimental cytotoxicity (pIC_50_) and the hydrophobicity index (cLogP, as detailed in Table S2). Figure 6 illustrates the positive correlation between pIC_50_ values and the predicted cLogP values. Notably, the results indicate a stronger correlation between the pIC_50_ and cLogP values obtained in MCF-7 cell lines (R = 0.95) compared to those in HT-29 (R = 0.84).

Of particular interest, CBHQ derivatives **5e** and **5f** exhibit higher pIC_50_ values for both the MCF-7 and HT-29 cell lines. This observation aligns with their greater ability to traverse the cell membrane, as evidenced by their higher cLogP values compared to those of the other synthesized cytotoxic hybrids. However, despite their superior pIC_50_ values in both cell lines, **5e** and **5f** display lower binding affinities for the evaluated proteins, including c-MET, TRKA, and HER2, compared to the rest of the cytotoxic hybrids, including **6a**–**c** and **6e**.

Based on these findings, the compounds with the best cytotoxicity values tend to be less polar and possess lower cLogP values (as indicated in Table S1). These characteristics correspond to the diacetylated CBHQs and CNQs, which also exhibit lower cLogP values and feature a planar bicyclic system due to the aromatization of the fused cycle with the quinone system. These factors enhance their ability to permeate the cell membrane.

Furthermore, it is possible to speculate that the CBHQs **5e** and **5f** may act as prodrugs. They could undergo hydrolysis through deacetylation within the cell, catalyzed by a “deacetylase” enzyme, releasing the molecules in the form of the CBHQs **4e** and **4f**. Subsequently, these benzohydroquinone compounds might exhibit an inhibitory effect on cancer-related kinases. This assumption is supported by their favorable binding energies in the active site of the kinase domain of EGFR, as detailed in Table S2.

### 2.5. In Silico Drug-Likeness, Toxicity Risks, and ADME Predictions

The drug-likeness scores for compounds **5e**, **5f**, and **6a**–**c**,**e** were computed using the DataWarrior algorithm, and the results are presented in Table 5. Notably, derivative **5f** stands out as the only compound with a positive drug-likeness value of 2.15. This significant finding suggests that compound **5f** could be a promising lead candidate for further investigation. It is noteworthy that **5f** incorporates essential structural elements, such as the hydroquinone and chalcone fragments, which are commonly found in approved drugs. Additionally, both **5f** and **5e** feature hydroquinone fragments with acetylation at positions 1 and 4. These substituents are known to contribute significantly to the enhancement of the antineoplastic cytotoxicity of potential anticancer agents.

In terms of toxicity risks, compounds **5e** and **5f** are likely to exhibit a high level of irritant risk, whereas compounds **6a**, **6c**, and **6e** are expected to have no adverse effects, except for **6b**, which may present a high irritant risk and low effects on the reproductive system (Table 5). The high irritant risk associated with **5e** and **5f** can be attributed to the acetylation in the hydroquinone moiety, while for **6b**, it is likely due to the presence of a methoxy group in the naphthoquinone moiety.

The predicted values for several pharmacokinetic parameters of compounds **5e** and **5f** as well as **6a**, **6b**, **6c**, and **6e** related to oral absorption, Caco-2 cell permeability, blood–brain barrier permeability, and binding to human serum albumin, among others, are summarized in Table S4. These ADME descriptor values indicate that the percentage of predicted oral absorption for these compounds ranges from 84% to 100%, suggesting good oral bioavailability. Furthermore, all the evaluated compounds demonstrate good to excellent predicted values for Caco-2 cell permeability, with QPlogBB values falling between −1.44 and −0.98. Additionally, all the tested compounds are within the range of interaction with human serum albumin, suggesting their potential transport by plasma proteins to the target site.

However, it is worth noting that all compounds may block HERG K+-channels, which play a crucial role in cardiac repolarization, potentially increasing the risk of cardiac arrhythmias. Moreover, some compounds, including **6a**, **6b**, and **6e**, are expected to have sufficient to excellent solubility in water, while **5e**, **5f**, and **6c** are considered higher-lipophilicity compounds, enhancing their ability to penetrate cell membranes. In terms of compliance with Jorgensen’s rule of three, practically all the CNQs exhibit 1 or 2 violations, all of which remain within permissible limits.

Moreover, nearly all the evaluated compounds meet Lipinski’s rule of five and its Weber extension criteria, except for **5e** and **5f** (mol_MW > 500 amu and QPlog/Po/w > 5). However, even these two compounds have violations that fall within acceptable limits (Table S5). These results collectively suggest that, from a pharmacokinetic perspective, most of these compounds hold promise as potential candidates for preclinical assays.

## 3. Materials and Methods

### 3.1. Chemistry

All chemical reactions were carried out using commercially available solvents and reagent grade chemicals without further purification. The initial substrate, 2-acetyl-6-(4-methyl-3-pentenyl)-5,8-dihydro-1,4-naphthohydroquinone (designated as compound 1), was synthesized according to the method we previously described [41]. To record the IR and NMR spectra and carry out the elemental analyses of C, H, and N of the synthesized compounds, the experimental conditions that we previously reported were used [38].

#### 3.1.1. Procedure for the Synthesis and Molecular Characterization of Precursor 2

##### Synthesis of 1-{1-Hydroxy-6-(4-methylpent-3-en-1-yl)-4-[(tetrahydro-2H-pyran-2-yl)oxy]-5,8-dihydronaphthalen-2-yl}ethanone (2)

First, 3,4-dihydro-2H-pyran (5.00 mmol) and 1.14 mmol of pyridinium p-toluenesulfonate were added to a solution of 2-acetyl-5,8-dihydro-6-(4-methyl-3-pentenyl)-1,4-naphthohydroquinone **1** (1.00 mmol) in CH_2_Cl_2_ (10 mL). The reaction mixture was stirred for 19 h at rt. Then, the reaction mixture was washed with distilled H_2_O (2 × 10 mL); after separating the phases, the organic phase was dried with anhydrous Na_2_SO_4_, filtered, and evaporated under reduced pressure. The crude product was purified by recrystallization using hexane as solvent. White solid (333 mg, 80%), m.p. 82–84 °C. IR υ_max_ cm^−1^ (film) 3407 (O-H), 1632 (C=O), and 1033 (C-O). ^1^H NMR (CDCl_3_, TMS, ppm) δ 1.66 (m, 10H, 2CH_3_, 2CH_2_, H13, H14, H3′, H4′), 1.91 (m, 2H, CH_2_, H2′), 2.17 (m, 4H, 2CH_2_, H10, H9), 2.58 (s, 3H, CH_3_, H16), 3.29 (m, 4H, 2CH_2_, H8, H5), 3.64 (m, 1H, CH_2_, H5′), 3.90 (m, 1H, CH_2_, H5′), 5.16 (t, 1H, *J* = 6.7 Hz, CH, H11), 5.33 (t, 1H, *J* = 3.2 Hz, CH, H1′), 5.64 (s, 1H, CH, H7), 7.28 (s, 1H, CH, H3), and 12.45 (s, 1H, OH, H1). ^13^C NMR (CDCl_3_, TMS, ppm) δ 17.7 (C13), 19.0 (C3′), 24.6 (C8), 25.3 (C4′), 25.7 (C14), 26.1 (C10), 26.6 (C16), 28.4 (C5), 30.7 (C2′), 37.2 (C9), 62.0 (C5′), 97.1 (C1′), 111.6 (C3), 116.0 (C2), 117.6 (C11), 124.1 (C7), 124.8 (C8a), 131.8 (C4a), 133.4 (C12), 135.2 (C6), 146.3 (C4), 155.5 (C1), and 204.0 (C15). Elemental analysis calculated for C_23_H_30_O_4_: C, 74.56; H, 8.16. Found: C, 74.51; H, 8.22.

#### 3.1.2. General Procedure for the Synthesis and Characterization of Compounds **3a**–**f**

A solution containing the precursor **2** (1.00 mmol) and 1.00 mmol of barium hydroxide octahydrate in ethanol (8 mL) was maintained by constantly stirring it for 10 min. Then, the equivalent of 1.10 mmoles of the respective benzaldehyde was added and maintaining by stirring it for 25 min at 90 °C. After the end of the reaction time, the mixture was added to an ice/water bath and then vacuum-filtered, obtaining the respective impure products **3a**–**f**.

##### Synthesis of (E)-1-{1-Hydroxy-6-(4-methylpent-3-en-1-yl)-4-[(tetrahydro-2H-pyran-2-yl)oxy]-5,8-dihydronaphthalen-2-yl}-3-phenylprop-2-en-1-one (**3a**)

This compound was synthesized by the general procedure, using precursor **2** and benzaldehyde, and purified by recrystallization using methanol as solvent. Yellow solid (275 mg, 60%), m.p. 139–140 °C. IR υ_max_ cm^−1^ (film) 3406 (O-H), 1637 (C=O), 1574 (C=C), and 1033 (C-O). ^1^H NMR (CDCl_3_, TMS, ppm) δ 1.70 (m, 10H, 2CH_3_, 2CH_2_, H13, H14, H3′, H4′), 1.94 (m, 2H, CH_2_, H2′), 2.19 (m, 4H, 2CH_2_, H10, H9), 3.33 (m, 4H, 2CH_2_, H8, H5), 3.68 (m, 1H, CH_2_, H5′), 3.95 (m, 1H, CH_2_, H5′), 5.19 (t, 1H, *J* = 6.8 Hz, CH, H11), 5.42 (t, 1H, *J* = 3.2 Hz, CH, H1′), 5.68 (s, 1H, CH, H7), 7.48 (m, 4H, 4CH, H3, H20, H21, H22), 7.61 (d, 1H, *J* = 15.7 Hz, CH, H16), 7.69 (d, 2H, *J* = 8.6 Hz, 2CH, H19, H23), 7.92 (d, 1H, *J* = 15.7 Hz, CH, H17), and 13.15 (s, 1H, OH, H1). ^13^C NMR (CDCl_3_, TMS, ppm) δ 17.7 (C13), 19.0 (C3′), 24.8 (C8), 25.3 (C4′), 25.7 (C14), 26.2 (C10), 28.4 (C5), 30.7 (C2′), 37.2 (C9), 62.0 (C5′), 97.2 (C1′), 110.7 (C3), 116.3 (C2), 117.7 (C11), 120.6 (C16), 124.1 (C7), 125.0 (C8a), 128.6 (C19, C23), 129.0 (C20, C22), 130.7 (C21), 131.8 (C4a), 133.4 (C12), 134.8 (C6), 135.5 (C18), 144.7 (C17), 146.4 (C4), 156.9 (C1), and 193.2 (C15). Elemental analysis calculated for C_30_H_34_O_4_: C, 78.57; H, 7.47. Found: C, 78.61; H, 7.43.

##### Synthesis of (E)-1-{1-Hydroxy-6-(4-methylpent-3-en-1-yl)-4-[(tetrahydro-2H-pyran-2-yl)oxy]-5,8-dihydronaphthalen-2-yl}-3-(4-methoxyphenyl)prop-2-en-1-one (**3b**)

This compound was synthesized by the general procedure, using precursor **2** and 4-methoxybenzaldehyde, and purified by recrystallization using ethanol as solvent. White solid (322 mg, 66%), m.p. 134–136 °C. IR υ_max_ cm^−1^ (film) 3428 (O-H), 1632 (C=O), 1605 (C=C), and 1172 (C-O). ^1^H NMR (CDCl_3_, TMS, ppm) δ 1.70 (m, 10H, 2CH_3_, 2CH_2_, H13, H14, H3′, H4′), 1.94 (m, 2H, CH_2_, H2′), 2.20 (m, 4H, 2CH_2_, H10, H9), 3.34 (m, 4H, 2CH_2_, H8, H5), 3.68 (m, 1H, CH_2_, H5′), 3.88 (s, 3H, CH_3_, H24), 3.95 (m, 1H, CH_2_, H5′), 5.19 (t, 1H, *J* = 6.7 Hz, CH, H11), 5.42 (t, 1H, *J* = 3.1 Hz, CH, H1′), 5.68 (s, 1H, CH, H7), 6.97 (d, 2H, *J* = 8.6 Hz, 2CH, H20, H22), 7.48 (m, 2H, 2CH, H3, H16), 7.64 (d, 2H, *J* = 8.6 Hz, 2CH, H19, H23), 7.90 (d, 1H, *J* = 15.5 Hz, CH, H17), and 13.26 (s, 1H, OH, H1). ^13^C NMR (CDCl_3_, TMS, ppm) δ 17.8 (C13), 19.0 (C3′), 24.8 (C8), 25.3 (C4′), 25.7 (C14), 26.2 (C10), 28.4 (C5), 30.7 (C2′), 37.2 (C9), 55.5 (C24), 62.0 (C5′), 97.1 (C1′), 110.7 (C3), 114.5 (C20, C22), 116.4 (C2), 117.7 (C11), 118.1 (C16), 124.1 (C7), 124.9 (C8a), 127.6 (C18), 130.5 (C19, C23), 131.8 (C4a), 133.4 (C12), 135.1 (C6), 144.6 (C17), 146.3 (C4), 156.9 (C1), 161.8 (C21), and 193.2 (C15). Elemental analysis calculated for C_31_H_36_O_5_: C, 76.20; H, 7.43. Found: C, 76.26; H, 7.39.

##### Synthesis of (E)-1-{1-Hydroxy-6-(4-methylpent-3-en-1-yl)-4-[(tetrahydro-2H-pyran-2-yl)oxy]-5,8-dihydronaphthalen-2-yl}-3-(4-methylphenyl)prop-2-en-1-one (**3c**)

This compound was synthesized by the general procedure, using precursor **2** and 4-methybenzaldehyde, and purified by recrystallization using ethanol as solvent. Orange solid (307 mg, 65%), m.p. 132–133 °C. IR υ_max_ cm^−1^ (film) 3492 (O-H), 1634 (C=O), and 1584 (C=C). ^1^H NMR (CDCl_3_, TMS, ppm) δ 1.69 (m, 10H, 2CH_3_, 2CH_2_, H13, H14, H3′, H4′), 1.94 (m, 2H, CH_2_, H2′), 2.19 (m, 4H, 2CH_2_, H10, H9), 2.42 (s, 3H, CH_3_, H24), 3.35 (m, 4H, 2CH_2_, H8, H5), 3.69 (m, 1H, CH_2_, H5′), 3.96 (td, 1H, *J* = 9.6 Hz, *J* = 3.3 Hz, CH_2_, H5′), 5.19 (t, 1H, *J* = 6.4 Hz, CH, H11), 5.41 (t, 1H, *J* = 3.1 Hz, CH, H1′), 5.67 (s, 1H, CH, H7), 7.25 (d, 2H, *J* = 8.1 Hz, 2CH, H20, H22), 7.49 (s, 1H, 1CH, H3), 7.55 (m, 3H, 3CH, H16, H19, H23), 7.90 (d, 1H, *J* = 15.7 Hz, CH, H17), and 13.16 (s, 1H, OH, H1). ^13^C NMR (CDCl_3_, TMS, ppm) δ 17.7 (C13), 19.0 (C24), 21.6 (C3′), 24.8 (C8), 25.3 (C4′), 25.7 (C14), 26.2 (C10), 28.4 (C5), 30.7 (C2′), 37.2 (C9), 62.0 (C5′), 97.2 (C1′), 110.7 (C3), 116.3 (C2), 117.7 (C11), 119.5 (C16), 124.2 (C7), 125.0 (C8a), 128.7 (C19, C23), 129.7 (C20, C22), 131.7 (C4a), 132.1 (C18), 133.5 (C12), 135.3 (C6), 141.3 (C21), 144.8 (C17), 146.3 (C4), 156.9 (C1), and 193.3 (C15). Elemental analysis calculated for C_31_H_36_O_4_: C, 78.78; H, 7.68. Found: C, 78.74; H, 7.72.

##### Synthesis of (E)-3-(2,4-Dichlorophenyl)1-{1-hydroxy-6-(4-methylpent-3-en-1-yl)-4-[(tetrahydro-2H-pyran-2-yl)oxy]-5,8-dihydronaphthalen-2-yl}prop-2-en-1-one (**3d**)

This compound was synthesized by the general procedure, using precursor **2** and 2,4-dichlorobenzaldehyde, and purified by recrystallization using methanol as solvent. Yellow solid (369 mg, 70%), m.p. 134–136 °C. IR υ_max_ cm^−1^ (film) 3442 (O-H), 1634 (C=O), and 1581 (C=C). ^1^H NMR (CDCl_3_, TMS, ppm) δ 1.70 (m, 10H, 2CH_3_, 2CH_2_, H13, H14, H3′, H4′), 1.94 (m, 2H, CH_2_, H2′), 2.20 (m, 4H, 2CH_2_, H10, H9), 3.34 (m, 4H, 2CH_2_, H8, H5), 3.66 (m, 1H, CH_2_, H5′), 3.92 (m, 1H, CH_2_, H5′), 5.19 (t, 1H, *J* = 6.6 Hz, CH, H11), 5.41 (t, 1H, *J* = 3.1 Hz, CH, H1′), 5.67 (s, 1H, CH, H7), 7.32 (dd, 1H, *J* = 8.4 Hz, *J* = 2.1 Hz, CH, H22), 7.44 (s, 1H, 1CH, H3), 7.49 (d, 1H, *J* = 2.1 Hz, CH, H20), 7.55 (d, 1H, *J* = 15.7 Hz, CH, H16), 7.69 (d, 1H, *J* = 8.4 Hz, CH, H23), 8.20 (d, 1H, *J* = 15.7 Hz, CH, H17), and 12.97 (s, 1H, OH, H1). ^13^C NMR (CDCl_3_, TMS, ppm) δ 17.8 (C13), 18.9 (C3′), 24.8 (C8), 25.3 (C4′), 25.8 (C14), 26.2 (C10), 28.5 (C5), 30.7 (C2′), 37.2 (C9), 62.0 (C5′), 97.0 (C1′), 110.5 (C3), 116.1 (C2), 117.6 (C11), 123.6 (C16), 124.1 (C7), 125.1 (C8a), 127.6 (C22), 128.7 (C20), 130.2 (C23), 131.8 (C4a), 131.8 (C18), 133.4 (C12), 135.8 (C19), 136.2 (C21), 136.6 (C6), 139.1 (C17), 146.4 (C4), 157.0 (C1), and 192.7 (C15). Elemental analysis calculated for C_30_H_32_Cl_2_O_4_: C, 68.31; H, 6.11. Found: C, 68.36; H, 6.08.

##### Synthesis of (E)-1-{1-Hydroxy-6-(4-methylpent-3-en-1-yl)-4-[(tetrahydro-2H-pyran-2-yl)oxy]-5,8-dihydronaphthalen-2-yl}-3-(2,3,4-trimethoxyphenyl)prop-2-en-1-one (**3e**)

This compound was synthesized by the general procedure, using precursor **2** and 2,3,4-trimethoxybenzaldehyde, and purified by recrystallization using ethanol as solvent. Red solid (357 mg, 65%), m.p. 97–99 °C. IR υ_max_ cm^−1^ (film) 3415 (O-H), 1631 (C=O), 1564 (C=C), and 1107 (C-O). ^1^H NMR (CDCl_3_, TMS, ppm) δ 1.70 (m, 10H, 2CH_3_, 2CH_2_, H13, H14, H3′, H4′), 2.09 (m, 6H, 3CH_2_, H2′, H10, H9), 3.33 (m, 4H, 2CH_2_, H8, H5), 3.69 (m, 1H, CH_2_, H5′), 3.92 (m, 7H, 2CH_3_, CH_2_, H25, H26, H5′), 4.00 (s, 3H, CH_3_, H24), 5.19 (t, 1H, *J* = 6.7 Hz, CH, H11), 5.39 (t, 1H, *J* = 3.1 Hz, CH, H1′), 5.67 (s, 1H, CH, H7), 6.74 (d, 1H, *J* = 8.3 Hz, CH, H22), 7.39 (d, 1H, *J* = 8.3 Hz, CH, H23), 7.52 (s, 1H, CH, H3), 7.69 (d, 1H, *J* = 15.8 Hz, CH, H16), 8.07 (d, 1H, *J* = 15.8 Hz, CH, H17), and 13.23 (s, 1H, OH, H1). ^13^C NMR (CDCl_3_, TMS, ppm) δ 17.7 (C13), 19.0 (C3′), 24.8 (C8), 25.3 (C4′), 25.7 (C14), 26.2 (C10), 28.4 (C5), 30.7 (C2′), 37.2 (C9), 56.1 (C24), 61.0 (C25), 61.3 (C26), 62.0 (C5′), 97.3 (C1′), 107.6 (C22), 110.8 (C3), 116.5 (C2), 117.7 (C11), 119.9 (C16), 121.9 (C18), 124.2 (C8a), 124.7 (C7), 124.8 (C23), 131.7 (C4a), 133.5 (C12), 135.0 (C6), 140.2 (C17), 142.5 (C20), 146.4 (C4), 154.0 (C19), 156.0 (C21), 156.8 (C1), and 193.6 (C15). Elemental analysis calculated for C_33_H_40_O_7_: C, 72.24; H, 7.35. Found: C, 72.20; H, 7.37.

##### Synthesis of (E)-1-{1-Hydroxy-6-(4-methylpent-3-en-1-yl)-4-[(tetrahydro-2H-pyran-2-yl)oxy]-5,8-dihydronaphthalen-2-yl}-3-(3,4,5-trimethoxyphenyl)prop-2-en-1-one (**3f**)

This compound was synthesized by the general procedure, using precursor **2** and 3,4,5-trimethoxybenzaldehyde, and purified by recrystallization using ethanol as solvent. Orange solid (340 mg, 62%), m.p. 99–100 °C. IR υ_max_ cm^−1^ (film) 3394 (O-H), 1631 (C=O), and 1563 (C=C). ^1^H NMR (CDCl_3_, TMS, ppm) δ 1.68 (m, 10H, 2CH_3_, 2CH_2_, H13, H14, H3′, H4′), 2.05 (m, 6H, 3CH_2_, H2′, H10, H9), 3.34 (m, 4H, 2CH_2_, H8, H5), 3.66 (m, 1H, CH_2_, H5′), 3.82 (m, 1H, CH_2_, H5′), 3.94 (m, 9H, 3CH_3_, H24, H25, H26), 5.19 (t, 1H, *J* = 5.8 Hz, CH, H11), 5.34 (t, 1H, *J* = 3.3 Hz, CH, H1′), 5.67 (s, 1H, CH, H7), 6.88 (s, 2H, 2CH, H19, H23), 7.46 (d, 2H, *J* = 15.8 Hz, 2CH, H16, H3), 7.82 (d, 1H, *J* = 15.8 Hz, CH, H17), and 13.09 (s, 1H, OH, H1). ^13^C NMR (CDCl_3_, TMS, ppm) δ 17.7 (C13), 19.2 (C3′), 24.8 (C8), 25.3 (C4′), 25.7 (C14), 26.2 (C10), 28.5 (C5), 30.8 (C2′), 37.2 (C9), 56.3 (C24, C26), 61.0 (C25), 62.3 (C5′), 97.7 (C1′), 105.9 (C19, C23), 111.2 (C3), 116.3 (C2), 117.7 (C11), 120.1 (C16), 124.1 (C7), 125.0 (C8a), 130.4 (C18), 131.7 (C4a), 133.4 (C12), 135.5 (C6), 140.7 (C21), 144.8 (C4), 146.4 (C17), 153.5 (C20, C22), 156.9 (C1), and 193.1 (C15). Elemental analysis calculated for C_33_H_40_O_7_: C, 72.24; H, 7.35. Found: C, 72.21; H, 7.36.

##### General Procedure for the Synthesis and Characterization of Compounds **4a**–**f**

Acid monohydrate 4-toluenesulfonic (0.80 mmol) was added to a solution of the respective compound **3a**–**f** (1.00 mmol) in methanol (10 mL). The reaction mixture was stirred for 3 h at rt. After the end of the reaction time, the mixture was added to an ice/water bath and then vacuum-filtered, obtaining the respective impure products **4a**–**f**.

##### Synthesis of (E)-1-[1,4-Dihydroxy-6-(4-methylpent-3-en-1-yl)-5,8-dihydronaphthalen-2-yl]-3-phenylprop-2-en-1-one (**4a**)

This compound was synthesized by the general procedure using precursor **3a** and purified by recrystallization using methanol as solvent. Orange solid (348 mg, 93%), m.p. 141–143 °C. IR υ_max_ cm^−1^ (film) 3403 (O-H), 1662 (C=O), and 1633 (C=C). ^1^H NMR (Acetone-d6, TMS, ppm) δ 1.63 (s, 3H, CH_3_, H13), 1.68 (s, 3H, CH_3_, H14), 2.20 (m, 4H, 2CH_2_, H10, H9), 3.30 (s, 4H, 2CH_2_, H8, H5), 5.19 (t, 1H, *J* = 6.7 Hz, CH, H11), 5.68 (s, 1H, CH, H7), 7.48 (m, 4H, 4CH, H3, H20, H21, H22), 7.85 (m, 4H, 4CH, H16, H17, H19, H23), 8.01 (s, 1H, OH, H4), and 12.95 (s, 1H, OH, H1). ^13^C NMR (Acetone-d6, TMS, ppm) δ 16.9 (C13), 24.5 (C8), 25.7 (C14), 26.0 (C10), 28.0 (C5), 37.0 (C9), 110.6 (C3), 116.4 (C2), 117.6 (C11), 120.9 (C16), 124.0 (C7), 124.2 (C8a), 128.7 (C19, C23), 129.0 (C20, C22), 130.8 (C21), 131.2 (C4a), 133.2 (C12), 133.5 (C6), 135.0 (C18), 144.3 (C17), 146.3 (C4), 155.3 (C1), and 193.3 (C15). Elemental analysis calculated for C_25_H_26_O_3_: C, 80.18; H, 7.00. Found: C, 80.25; H, 6.96.

##### Synthesis of (E)-1-[1,4-Dihydroxy-6-(4-methylpent-3-en-1-yl)-5,8-dihydronaphthalen-2-yl]-3-(4-methoxyphenyl)prop-2-en-1-one (**4b**)

This compound was synthesized by the general procedure using precursor **3b** and purified by recrystallization using methanol as solvent. Orange solid (388 mg, 96%), m.p. 156–158 °C. IR υ_max_ cm^−1^ (film) 3353 (O-H), 1631 (C=O), and 1605 (C=C). ^1^H NMR (Acetone-d6, TMS, ppm) δ 1.63 (s, 3H, CH_3_, H13), 1.68 (s, 3H, CH_3_, H14), 2.19 (m, 4H, 2CH_2_, H10, H9), 3.29 (m, 4H, 2CH_2_, H8, H5), 3.88 (s, 3H, CH_3_, H24), 5.18 (t, 1H, *J* = 6.8 Hz, CH, H11), 5.67 (s, 1H, CH, H7), 7.05 (d, 2H, *J* = 8.6 Hz, 2CH, H20, H22), 7.44 (s, 1H, CH, H3), 7.67 (d, 1H, *J* = 15.7 Hz, CH, H16), 7.80 (d, 2H, *J* = 8.6 Hz, 2CH, H19, H23), 7.87 (d, 1H, *J* = 15.7 Hz, CH, H17), and 7.98 (s, 1H, OH, H4), 13.07 (s, 1H, OH, H1). ^13^C NMR (Acetone-d6, TMS, ppm) δ 16.9 (C13), 24.5 (C8), 25.0 (C14), 26.0 (C10), 28.0 (C5), 37.0 (C9), 55.0 (C24), 110.5 (C3), 114.5 (C20, C22), 116.4 (C2), 117.6 (C11), 118.2 (C16), 124.1 (C7), 124.1 (C8a), 127.5 (C18), 130.6 (C19, C23), 131.2 (C4a), 132.8 (C12), 133.5 (C6), 144.4 (C17), 146.2 (C4), 155.2 (C1), 162.1 (C21), and 193.2 (C15). Elemental analysis calculated for C_26_H_28_O_4_: C, 77.20; H, 6.98. Found: C, 77.18; H, 7.03.

##### Synthesis of (E)-1-[1,4-Dihydroxy-6-(4-methylpent-3-en-1-yl)-5,8-dihydronaphthalen-2-yl]-3-(4-methylphenyl)prop-2-en-1-one (**4c**)

This compound was synthesized by the general procedure using precursor **3c** and purified by recrystallization using methanol as solvent. Orange solid (361 mg, 93%), m.p. 185–186 °C. IR υ_max_ cm^−1^ (film) 3272 (O-H), 1630 (C=O), and 1558 (C=C). ^1^H NMR (Acetone-d6, TMS, ppm) δ 1.62 (s, 3H, CH_3_, H13), 1.67 (s, 3H, CH_3_, H14), 2.18 (m, 4H, 2CH_2_, H10, H9), 2.38 (s, 3H, CH_3_, H24), 3.28 (s, 4H, 2CH_2_, H8, H5), 5.17 (t, 1H, *J* = 6.2 Hz, CH, H11), 5.66 (s, 1H, CH, H7), 7.28 (d, 2H, *J* = 7.7 Hz, 2CH, H20, H22), 7.41 (s, 1H, CH, H3), 7.68 (m, 3H, 3CH, H16, H19, H23), 7.85 (d, 1H, *J* = 15.3 Hz, CH, H17), 7.96 (s, 1H, OH, H4), and 12.96 (s, 1H, OH, H1). ^13^C NMR (Acetone-d6, TMS, ppm) δ 17.1 (C13), 20.8 (C24), 24.5 (C8), 25.1 (C14), 26.0 (C10), 28.1 (C5), 37.1 (C9), 110.4 (C3), 116.4 (C2), 117.6 (C11), 119.7 (C16), 124.1 (C7), 124.2 (C8a), 128.7 (C19, C23), 129.7 (C20, C22), 131.2 (C4a), 132.2 (C18), 133.0 (C12), 133.5 (C6), 141.2 (C21), 144.4 (C17), 146.3 (C4), 155.3 (C1), and 193.1 (C15). Elemental analysis calculated for C_26_H_28_O_3_: C, 80.38; H, 7.26. Found: C, 80.43; H, 7.20.

##### Synthesis of (E)-3-(2,4-Dichlorophenyl)-1-[1,4-dihydroxy-6-(4-methylpent-3-en-1-yl)-5,8-dihydronaphthalen-2-yl]prop-2-en-1-one (**4d**)

This compound was synthesized by the general procedure using precursor **3d** and purified by recrystallization using methanol as solvent. Yellow solid (421 mg, 95%), m.p. 155–157 °C. IR υ_max_ cm^−1^ (film) 3390 (O-H), 1634 (C=O), and 1581 (C=C). ^1^H NMR (Acetone-d6, TMS, ppm) δ 1.63 (s, 3H, CH_3_, H13), 1.68 (s, 3H, CH_3_, H14), 2.19 (m, 4H, 2CH_2_, H10, H9), 2.89 (s, 4H, 2CH_2_, H8, H5), 5.19 (t, 1H, *J* = 6.3 Hz, CH, H11), 5.67 (s, 1H, CH, H7), 7.49 (m, 2H, 2CH, H22, H3), 7.63 (d, 1H, *J* = 2.1 Hz, CH, H20), 7.88 (d, 1H, *J* = 15.8 Hz, CH, H16), 8.12 (m, 3H, 2CH, H23, H17, OH, H4), and 12.78 (s, 1H, OH, H1). ^13^C NMR (Acetone-d6, TMS, ppm) δ 16.9 (C13), 24.5 (C8), 24.9 (C14), 26.0 (C10), 28.1 (C5), 37.0 (C9), 110.7 (C3), 116.3 (C2), 117.6 (C11), 124.1 (C16), 124.3 (C8a), 124.3 (C7), 127.9 (C22), 129.5 (C20), 129.7 (C23), 131.2 (C4a), 131.8 (C18), 133.5 (C12), 133.8 (C19), 135.6 (C21), 136.2 (C6), 137.9 (C17), 146.4 (C4), 155.4 (C1), and 192.7 (C15). Elemental analysis calculated for C_25_H_24_Cl_2_O_3_: C, 67.73; H, 5.46. Found: C, 67.79; H, 5.51.

##### Synthesis of (E)-1-[1,4-Dihydroxy-6-(4-methylpent-3-en-1-yl)-5,8-dihydronaphthalen-2-yl]-3-(2,3,4-trimethoxyphenyl)prop-2-en-1-one (**4e**)

This compound was synthesized by the general procedure using precursor **3e** and purified by recrystallization using methanol as solvent. Red solid (441 mg, 95%), m.p. 149–151 °C. IR υ_max_ cm^−1^ (film) 3434 (O-H), 1633 (C=O), and 1579 (C=C). ^1^H NMR (Acetone-d6, TMS, ppm) δ 1.63 (s, 3H, CH_3_, H13), 1.68 (s, 3H, CH_3_, H14), 2.20 (m, 4H, 2CH_2_, H10, H9), 3.29 (s, 4H, 2CH_2_, H8, H5), 3.85 (s, 3H, CH_3_, H25), 3.93 (s, 3H, CH_3_, H26), 3.98 (s, 3H, CH_3_, H24), 5.19 (t, 1H, *J* = 6.8 Hz, CH, H11), 5.68 (s, 1H, CH, H7), 6.92 (d, 1H, *J* = 8.9 Hz, CH, H22), 7.40 (s, 1H, CH, H3), 7.59 (d, 1H, *J* = 8.9 Hz, CH, H23), 7.79 (d, 1H, *J* = 16.0 Hz, CH, H16), 8.02 (s, 1H, OH, H4), 8.07 (d, 1H, *J* = 16.0 Hz, CH, H17), and 13.08 (s, 1H, OH, H1). ^13^C NMR (Acetone-d6, TMS, ppm) δ 16.9 (C13), 24.5 (C8), 24.9 (C14), 26.0 (C10), 28.0 (C5), 37.0 (C9), 55.6 (C24), 60.1 (C25), 60.9 (C26), 108.2 (C22), 110.2 (C3), 116.5 (C2), 117.7 (C11), 119.4 (C16), 121.4 (C18), 124.1 (C23), 124.2 (C8a), 124.4 (C7), 131.1 (C4a), 132.8 (C12), 133.6 (C6), 139.6 (C17), 142.6 (C20), 146.3 (C4), 153.9 (C19), 155.2 (C21), 156.5 (C1), and 193.4 (C15). Elemental analysis calculated for C_28_H_32_O_6_: C, 72.39; H, 6.94. Found: C, 72.43; H, 6.90.

##### Synthesis of (E)-1-[1,4-Dihydroxy-6-(4-methylpent-3-en-1-yl)-5,8-dihydronaphthalen-2-yl]-3-(3,4,5-trimethoxyphenyl)prop-2-en-1-one (**4f**)

This compound was synthesized by the general procedure using precursor **3f** and purified by recrystallization using methanol as solvent. Orange solid (432 mg, 93%), m.p. 168–169 °C. IR υ_max_ cm^−1^ (film) 3433 (O-H), 1634 (C=O), and 1582 (C=C). ^1^H NMR (Acetone-d6, TMS, ppm) δ 1.66 (m, 6H, 2CH_3_, H13, H14), 2.22 (m, 4H, 2CH_2_, H10, H9), 3.30 (s, 4H, 2CH_2_, H8, H5), 3.81 (s, 3H, CH_3_, H25), 3.93 (s, 6H, 2CH_3_, H24, H26), 5.19 (t, 1H, *J* = 6.5 Hz, CH, H11), 5.68 (s, 1H, CH, H7), 7.19 (s, 2H, 2CH, H19, H23), 7.40 (s, 1H, CH, H3), 7.74 (d, 1H, *J* = 15.3 Hz, CH, H16), 7.84 (d, 1H, *J* = 15.3 Hz, CH, H17), 7.94 (s, 1H, OH, H4), and 13.03 (s, 1H, OH, H1). ^13^C NMR (Acetone-d6, TMS, ppm) δ 16.9 (C13), 24.5 (C8), 25.0 (C14), 26.0 (C10), 28.0 (C5), 37.0 (C9), 55.7 (C24, C26), 69.8 (C25), 106.5 (C19, C23), 110.7 (C3), 116.4 (C2), 117.6 (C11), 119.9 (C16), 124.1 (C7), 124.2 (C8a), 130.3 (C18), 131.2 (C4a), 133.1 (C12), 133.5 (C6), 141.0 (C21), 144.9 (C4), 146.2 (C17), 153.8 (C20, C22), 155.3 (C1), and 193.2 (C15). Elemental analysis calculated for C_28_H_32_O_6_: C, 72.39; H, 6.94. Found: C, 72.44; H, 6.92.

##### General Procedure for the Synthesis and Characterization of Compounds **5′b**–**d** and **5e**,**f**

A total of 0.50 mL (5,3 mmol) of acetic anhydride was added to a solution of the respective compound **4b**–**f** (0.25 mmol) in pyridine (0.50 mL), and the reaction mixture was maintained in the dark with occasional stirring for 24 h at room temperature. After the end of the reaction time, the mixture was added to an ice/water bath. Subsequently, the mixture was dissolved with CH_2_Cl_2_ (40 mL), and successive extractions were performed with 10% HCl solution (2 × 20 mL) and with H_2_O (2 × 10 mL) until a neutral pH of the aqueous phase was attained. The organic phase was dried with anhydrous Na_2_SO_4_, filtered, and evaporated under reduced pressure. The crude product was purified by column chromatography with hexane/ethyl acetate as eluent in varying proportions.

##### Synthesis of (E)-4-Hydroxy-3-[3-(4-methoxyphenyl)prop-2-enoyl]-7-(4-methylpent-3-en-1-yl)-5,8-dihydronaphthalen-1-yl ethanoate (**5′b**)

This compound was synthesized by the general procedure using precursor **4b** and purified by CC with hexane/ethyl acetate 2:1. Yellow solid (94 mg, 85%), m.p. 176–177 °C. IR υ_max_ cm^−1^ (film) 3391 (O-H), 1758 (C=O), and 1572 (C=C). ^1^H NMR (CDCl_3_, TMS, ppm) δ 1.68 (m, 6H, 2CH_3_, H13, H14), 2.17 (m, 4H, 2CH_2_, H10, H9), 2.39 (s, 3H, CH_3_, H2′), 3.15 (s, 2H, CH_2_, H8), 3.38 (s, 2H, CH_2_, H5), 3.88 (s, 3H, CH_3_, H24), 5.16 (t, 1H, *J* = 6.6 Hz, CH, H11), 5.67 (s, 1H, CH, H7), 6.96 (d, 2H, *J* = 8.6 Hz, 2CH, H20, H22), 7.43 (d, 2H, *J* = 15.7 Hz, 2CH, H16, H3), 7.64 (d, 2H, *J* = 8.6 Hz, 2CH, H19, H23), 7.90 (d, 1H, *J* = 15.6 Hz, CH, H17), and 13.32 (s, 1H, OH, H1). ^13^C NMR (CDCl_3_, TMS, ppm) δ 17.8 (C13), 20.9 (C2′), 24.7 (C8), 25.7 (C14), 26.1 (C10), 28.2 (C5), 37.0 (C9), 55.4 (C24), 114.5 (C20, C22), 116.8 (C2), 117.6 (C3), 117.9 (C11), 118.9 (C16), 123.9 (C7), 125.7 (C8a), 127.4 (C18), 130.6 (C19, C23), 131.9 (C4a), 132.6 (C12), 136.4 (C6), 139.9 (C4), 145.3 (C17), 159.3 (C1), 162.0 (C21), 169.9 (C1′), and 192.8 (C15). Elemental analysis calculated for C_28_H_30_O_5_: C, 75.31; H, 6.77. Found: C, 75.26; H, 6.80.

##### Synthesis of (E)-4-Hydroxy-7-(4-methylpent-3-en-1-yl)-3-[3-(4-methylphenyl)prop-2-enoyl]-5,8-dihydronaphthalen-1-yl ethanoate (**5′c**)

This compound was synthesized by the general procedure using precursor **4c** and purified by CC with hexane/ethyl acetate 2:1. Yellow solid (99 mg, 90%), m.p. 168–169 °C. IR υ_max_ cm^−1^ (film) 3389 (O-H), 1757 (C=O), and 1583 (C=C). ^1^H NMR (CDCl_3_, TMS, ppm) δ 1.64 (s, 3H, CH_3_, H13), 1.72 (s, 3H, CH_3_, H14), 2.19 (m, 4H, 2CH_2_, H10, H9), 2.41 (m, 6H, 2CH_3_, H24, H2′), 3.14 (s, 2H, CH_2_, H8), 3.39 (s, 2H, CH_2_, H5), 5.16 (t, 1H, *J* = 6.8 Hz, CH, H11), 5.68 (s, 1H, CH, H7), 7.26 (m, 2H, 2CH, H20, H22), 7.53 (m, 4H, 4CH, H3, H16, H19, H23), 7.92 (d, 1H, *J* = 15.5 Hz, CH, H17), and 13.26 (s, 1H, OH, H1). ^13^C NMR (CDCl_3_, TMS, ppm) δ 17.8 (C13), 20.8 (C24), 21.6 (C2′), 24.7 (C8), 25.7 (C14), 26.1 (C10), 28.3 (C5), 37.0 (C9), 116.7 (C2), 117.9 (C3), 118.9 (C11), 119.0 (C16), 123.9 (C7), 125.8 (C8a), 128.8 (C19, C23), 129.8 (C20, C22), 131.9 (C4a), 131.9 (C18), 132.6 (C12), 136.6 (C6), 140.0 (C4), 141.6 (C21), 145.5 (C17), 159.3 (C1), 169.8 (C1′), and 193.0 (C15). Elemental analysis calculated for C_28_H_30_O_4_: C, 78.11; H, 7.02. Found: C, 78.17; H, 6.98.

##### Synthesis of (E)-3-[3-(2,4-Dichlorophenyl)prop-2-enoyl]-4-hydroxy-7-(4-methylpent-3-en-1-yl)-5,8-dihydronaphthalen-1-yl ethanoate (**5′d**)

This compound was synthesized by the general procedure using precursor **4d** and purified by CC with hexane/ethyl acetate 2:1. Yellow solid (112 mg, 93%), m.p. 196–197 °C. IR υ_max_ cm^−1^ (film) 3379 (O-H), 1755 (C=O), and 1585 (C=C). ^1^H NMR (CDCl_3_, TMS, ppm) δ 1.68 (m, 6H, 2CH_3_, H13, H14), 2.17 (m, 4H, 2CH_2_, H10, H9), 2.38 (s, 3H, CH_3_, H2′), 3.13 (s, 2H, CH_2_, H8), 3.38 (s, 2H, CH_2_, H5), 5.15 (t, 1H, *J* = 6.4 Hz, CH, H11), 5.67 (s, 1H, CH, H7), 7.31 (d, 1H, *J* = 8.3 Hz, CH, H22), 7.42 (s, 1H, CH, H3), 7.49 (m, 2H, 2CH, H16, H20), 7.71 (d, 1H, *J* = 8.3 Hz, CH, H23), 8.23 (d, 1H, *J* = 15.5 Hz, CH, H17), and 13.05 (s, 1H, OH, H1). ^13^C NMR (CDCl_3_, TMS, ppm) δ 17.7 (C13), 20.8 (C2′), 24.7 (C8), 25.7 (C14), 26.1 (C10), 28.3 (C5), 37.0 (C9), 116.5 (C2), 117.8 (C3), 118.9 (C11), 123.0 (C7), 123.9 (C16), 126.0 (C8a), 127.6 (C22), 128.6 (C20), 130.2 (C23), 131.5 (C4a), 132.0 (C18), 132.5 (C12), 136.4 (C19), 136.9 (C21), 137.2 (C6), 139.7 (C17), 140.0 (C4), 159.5 (C1), 169.8 (C1′), and 192.3 (C15). Elemental analysis calculated for C_27_H_26_Cl_2_O_4_: C, 66.81; H, 5.40. Found: C, 66.87; H, 5.35.

##### Synthesis of (E)-2-[3-(2,3,4-Trimethoxyphenyl)prop-2-enoyl]-6-(4-methylpent-3-en-1-yl)-5,8-dihydronaphthalen-1,4-diyl diethanoate (**5e**)

This compound was synthesized by the general procedure using precursor **4e** and purified by CC with hexane/ethyl acetate 3:1. Orange solid (121 mg, 87%), m.p. 143–144 °C. IR υ_max_ cm^−1^ (film) 1766 (C=O), and 1585 (C=C). ^1^H NMR (CDCl_3_, TMS, ppm) δ 1.67 (m, 6H, 2CH_3_, H13, H14), 2.15 (m, 4H, 2CH_2_, H10, H9), 2.33 (m, 6H, 2CH_3_, H2′, H4′), 3.22 (m, 4H, 2CH_2_, H8, H5), 3.91 (m, 9H, 3CH_3_, H24, H25, H26), 5.14 (m, 1H, CH, H11), 5.59 (s, 1H, CH, H7), 6.71 (d, 1H, *J* = 8.7 Hz, CH, H22), 7.19 (d, 1H, *J* = 16.1 Hz, CH, H16), 7.34 (d, 2H, *J* = 8.7 Hz, CH, H3, H23), and 7.84 (d, 1H, *J* = 16.1 Hz, CH, H17). ^13^C NMR (CDCl_3_, TMS, ppm) δ 17.7 (C13), 20.8 (C2′), 20.8 (C4′), 25.3 (C8), 25.7 (C14), 26.0 (C10), 27.9 (C5), 37.0 (C9), 56.1 (C26), 60.9 (C25), 61.5 (C24), 107.6 (C22), 116.9 (C3), 120.7 (C16), 121.7 (C18), 123.8 (C7), 123.9 (C11), 124.0 (C23), 130.3 (C4a), 130.4 (C2), 132.0 (C8a), 132.6 (C12), 133.3 (C20), 140.8 (C17), 142.4 (C6), 144.5 (C4), 145.7 (C1), 153.9 (C19), 156.0 (C21), 169.0 (C1′, C3′), and 190.5 (C15). Elemental analysis calculated for C_32_H_36_O_8_: C, 70.06; H, 6.61. Found: C, 70.11; H, 6.57.

##### Synthesis of (E)-2-[3-(3,4,5-Trimethoxyphenyl)prop-2-enoyl]-6-(4-methylpent-3-en-1-yl)-5,8-dihydronaphthalen-1,4-diyl diethanoate (**5f**)

This compound was synthesized by the general procedure using precursor **4f** and purified by CC with hexane/ethyl acetate 3:1. Orange solid (115 mg, 85%), m.p. 142–143 °C. IR υ_max_ cm^−1^ (film) 1766 (C=O), and 1580 (C=C). ^1^H NMR (CDCl_3_, TMS, ppm) δ 1.64 (s, 3H, CH_3_, H13), 1.71 (s, 3H, CH_3_, H14), 2.16 (m, 4H, 2CH_2_, H10, H9), 2.29 (s, 3H, CH_3_, H2′), 2.37 (s, 3H, CH_3_, H4′), 3.18 (m, 2H, CH_2_, H5), 3.28 (m, 2H, CH_2_, H8), 3.91 (s, 9H, 3CH_3_, H24, H25, H26), 5.14 (m, 1H, CH, H11), 5.60 (s, 1H, CH, H7), 6.82 (s, 2H, 2CH, H19, H23), 7.02 (d, 1H, *J* = 16.0 Hz, CH, H16), 7.28 (s, 1H, CH, H3), and 7.53 (d, 1H, *J* = 16.0 Hz, CH, H17). ^13^C NMR (CDCl_3_, TMS, ppm) δ 17.7 (C13), 20.8 (C2′), 20.9 (C4′), 25.3 (C8), 25.7 (C14), 26.0 (C10), 27.9 (C5), 36.9 (C9), 56.2 (C24, C26), 56.3 (C25), 105.7 (C19, C23), 116.4 (C3), 120.7 (C16), 123.8 (C7), 124.5 (C11), 130.0 (C18), 130.1 (C4a), 130.5 (C2), 132.0 (C8a), 132.8 (C12), 133.4 (C6), 140.6 (C21), 144.3 (C4), 145.6 (C1), 146.2 (C17), 153.8 (C20, C22), 169.0 (C1′, C3′), and 190.7 (C15). Elemental analysis calculated for C_32_H_36_O_8_: C, 70.06; H, 6.61. Found: C, 70.13; H, 6.56.

##### General Procedure for the Synthesis and Characterization of Compounds **6a**–**e**

2,3-dichloro-5,6-dicyanobenzoquinone (1.05 mmol) was added to a solution of the respective compound **4a**–**e** (0.50 mmol) in CH_2_Cl_2_ (15 mL). The reaction mixture was stirred for 30 min at rt. Then, the mixture was filtered over silica gel 230–400 mesh, and the organic solution was extracted with 5% NaHCO_3_ solution (2 × 10 mL) and H_2_O (1 × 20 mL). The organic phase was dried with anhydrous Na_2_SO_4_, filtered, and evaporated under reduced pressure, obtaining the respective impure products **6a**-**e**.

##### Synthesis of (E)-6-(4-Methylpent-3-en-1-yl)-2-(3-phenylprop-2-enoyl)naphthalene-1,4-dione (**6a**)

This compound was synthesized by the general procedure using precursor **4a** and purified by recrystallization using hexane as solvent. Yellow solid (74 mg, 40%), m.p. 134–137 °C. IR υ_max_ cm^−1^ (film) 1654 (C=O), and 1618 (C=C). ^1^H NMR (CDCl_3_, TMS, ppm) δ 1.55 (s, 3H, CH_3_, H13), 1.70 (s, 3H, CH_3_, H14), 2.38 (m, 2H, CH_2_, H10), 2.82 (t, 2H, *J* = 7.3 Hz, CH_2_, H9), 5.14 (m, 1H, CH, H11), 7.19 (m, 2H, H3, H16), 7.44 (m, 3H, 3CH, H20, H21, H22), 7.64 (m, 4H, 4CH, H17, H19, H23, H7), 7.94 (d, 1H, *J* = 1.9 Hz, CH, H5), and 8.07 (d, 1H, *J* = 7.9 Hz, CH, H8). ^13^C NMR (CDCl_3_, TMS, ppm) δ 17.8 (C13), 25.7 (C14), 29.3 (C10), 36.3 (C9), 122.5 (C11), 123.6 (C16), 125.3 (C5), 127.1 (C8), 128.9 (C19, C23), 129.1 (C20, C22), 129.8 (C8a), 131.3 (C21), 131.8 (C4a), 133.4 (C12), 134.7 (C18), 134.8 (C3), 136.9 (C7), 143.4 (C2), 146.5 (C17), 150.1 (C6), 183.4 (C4), 185.3 (C1), and 190.2 (C15). Elemental analysis calculated for C_25_H_22_O_3_: C, 81.06; H, 5.99. Found: C, 81.00; H, 6.03.

##### Synthesis of (E)-2-[3-(4-Methoxyphenyl)prop-2-enoyl]-6-(4-methylpent-3-en-1-yl) naphthalene-1,4-dione (**6b**)

This compound was synthesized by the general procedure using precursor **4b** and purified by recrystallization using hexane as solvent. Orange solid (112 mg, 56%), m.p. 110–112 °C. IR υ_max_ cm^−1^ (film) 1668 (C=O), and 1600 (C=C). ^1^H NMR (CDCl_3_, TMS, ppm) δ 1.55 (s, 3H, CH_3_, H13), 1.70 (s, 3H, CH_3_, H14), 2.39 (c, 2H, *J* = 7.6 Hz, CH_2_, H10), 2.82 (t, 2H, *J* = 7.6 Hz, CH_2_, H9), 3.87 (s, 3H, CH_3_, H24), 5.15 (t, 1H, *J* = 7.5 Hz, CH, H11), 6.94 (d, 2H, *J* = 8.6 Hz, 2CH, H20, H22), 7.07 (d, 2H, *J* = 15.7 Hz, 2CH, H3, H16), 7.60 (m, 4H, 4CH, H17, H19, H23, H7), 7.94 (d, 1H, *J* = 1.8 Hz, CH, H5), and 8.07 (d, 1H, *J* = 7.9 Hz, CH, H8). ^13^C NMR (CDCl_3_, TMS, ppm) δ 17.7 (C13), 25.7 (C14), 29.4 (C10), 36.3 (C9), 55.5 (C24), 114.6 (C20, C22), 122.5 (C11), 123.1 (C16), 126.2 (C5), 126.8 (C18), 127.0 (C8), 129.8 (C8a), 130.8 (C19, C23), 131.8 (C4a), 133.4 (C12), 134.8 (C3), 136.6 (C7), 146.3 (C2), 146.6 (C17), 150.0 (C6), 162.3 (C21), 183.4 (C4), 185.4 (C1), and 190.1 (C15). Elemental analysis calculated for C_26_H_24_O_4_: C, 77.98; H, 6.04. Found: C, 78.02; H, 6.01.

##### Synthesis of (E)-6-(4-Methylpent-3-en-1-yl)-2-[3-(4-methylphenyl)prop-2-enoyl] naphthalene-1,4-dione (**6c**)

This compound was synthesized by the general procedure using precursor **4c** and purified by recrystallization using hexane as solvent. Yellow solid (111 mg, 58%), m.p. 115–117 °C. IR υ_max_ cm^−1^ (film) 1666 (C=O), and 1601 (C=C). ^1^H NMR (CDCl_3_, TMS, ppm) δ 1.63 (m, 6H, 2CH_3_, H13, H14), 2.40 (m, 5H, CH_2_, CH_3_, H10, H24), 2.81 (t, 2H, *J* = 7.7 Hz, CH_2_, H9), 5.15 (t, 1H, *J* = 7.4 Hz, CH, H11), 7.20 (m, 4H, 4CH, H3, H16, H20, H22), 7.51 (d, 2H, *J* = 8.3 Hz, 2CH, H19, H23), 7.64 (m, 2H, 2CH, H17, H7), 7.94 (d, 1H, *J* = 1.7 Hz, CH, H5), and 8.07 (d, 1H, *J* = 7.9 Hz, CH, H8). ^13^C NMR (CDCl_3_, TMS, ppm) δ 17.7 (C13), 21.7 (C24), 25.7 (C14), 29.3 (C10), 36.3 (C9), 122.5 (C11), 124.3 (C16), 126.2 (C5), 127.0 (C8), 129.0 (C20, C22), 129.8 (C8a), 129.8 (C19, C23), 131.4 (C12), 131.8 (C18), 133.3 (C4a), 134.7 (C3), 136.7 (C7), 142.1 (C21), 146.1 (C2), 146.8 (C17), 150.0 (C6), 183.4 (C4), 185.3 (C1), and 190.2 (C15). Elemental analysis calculated for C_26_H_24_O_3_: C, 81.22; H, 6.29. Found: C, 81.15; H, 6.34.

##### Synthesis of (E)-2-[3-(2,4-Dichlorophenyl)prop-2-enoyl]-6-(4-methylpent-3-en-1-yl) naphthalene-1,4-dione (**6d**)

This compound was synthesized by the general procedure using precursor **4d** and purified by recrystallization using hexane as solvent. Orange solid (92 mg, 42%), m.p. 86–88 °C. IR υ_max_ cm^−1^ (film) 1668 (C=O), and 1624 (C=C). ^1^H NMR (CDCl_3_, TMS, ppm) δ 1.55 (s, 3H, CH_3_, H13), 1.69 (s, 3H, CH_3_, H14), 2.39 (m, 2H, CH_2_, H10), 2.82 (m, 2H, CH_2_, H9), 5.14 (t, 1H, *J* = 7.4 Hz, CH, H11), 7.20 (d, 1H, *J* = 2.8 Hz, CH, H20), 7.29 (m, 2H, 2CH, H3, H22), 7.47 (m, 2H, 2CH, H7, H16), 7.66 (m, 2H, 2CH, H23, H5), and 8.00 (m, 2H, 2CH, H17, H8). ^13^C NMR (CDCl_3_, TMS, ppm) δ 17.7 (C13), 25.7 (C14), 29.3 (C10), 36.3 (C9), 122.4 (C11), 126.3 (C16), 127.0 (C22), 127.5 (C20), 127.7 (C5), 128.7 (C8), 129.7 (C8a), 130.2 (C3), 131.1 (C18), 131.8 (C21), 133.4 (C12), 134.8 (C7), 136.4 (C4a), 137.3 (C19), 137.6 (C23), 140.1 (C17), 145.4 (C2), 150.2 (C6), 183.5 (C4), 185.1 (C1), and 189.5 (C15). Elemental analysis calculated for C_25_H_20_Cl_2_O_3_: C, 68.35; H, 4.59. Found: C, 68.43; H, 4.55.

##### Synthesis of (E)-2-[3-(2,3,4-Trimethoxyphenyl)prop-2-enoyl]-6-(4-methylpent-3-en-1-yl) naphthalene-1,4-dione (**6e**)

This compound was synthesized by the general procedure, using precursor **4e** and purified by recrystallization using hexane as solvent. Red solid (92 mg, 40%), m.p. 118–120 °C. IR υ_max_ cm^−1^ (film) 1666 (C=O), and 1596 (C=C). ^1^H NMR (CDCl_3_, TMS, ppm) δ 1.56 (s, 3H, CH_3_, H13), 1.70 (s, 3H, CH_3_, H14), 2.37 (m, 2H, CH_2_, H10), 2.82 (m, 2H, CH_2_, H9), 3.91 (m, 9H, 3CH_3_, H24, H25, H26), 5.15 (m, 1H, CH, H11), 6.73 (d, 1H, *J* = 8.8 Hz, CH, H22), 7.11 (s, 1H, CH, H3), 7.18 (d, 1H, *J* = 16.5 Hz, CH, H16), 7.38 (d, 1H, *J* = 8.8 Hz, CH, H23), 7.62 (dd, 1H, *J* = 8.3 Hz, *J* = 1.9 Hz, CH, H7), 7.9 (d, 1H, *J* = 16.5 Hz, CH, H17), 7.94 (d, 1H, *J* = 1.8 Hz, CH, H5), and 8.06 (d, 1H, *J* = 8.1 Hz, CH, H8). ^13^C NMR (CDCl_3_, TMS, ppm) δ 17.7 (C13), 25.7 (C14), 29.3 (C10), 36.3 (C9), 56.2 (C26), 60.9 (C26), 61.5 (C24), 107.7 (C22), 121.2 (C18), 122.5 (C11), 124.2 (C16), 124.3 (C23), 126.2 (C5), 127.0 (C8), 129.8 (C8a), 131.8 (C3), 133.3 (C12), 134.7 (C7), 136.5 (C4a), 142.4 (C17), 142.5 (C2), 146.5 (C20), 149.9 (C6), 154.1 (C19), 156.6 (C21), 183.4 (C4), 185.4 (C1), and 190.3 (C15). Elemental analysis calculated for C_28_H_28_O_6_: C, 73.03; H, 6.13. Found: C, 73.07; H, 6.04.

### 3.2. Antiproliferative Assay

MCF-7 (human breast adenocarcinoma) and HT-29 (human colon adenocarcinoma) cell lines were obtained from the American Type Culture Collection (ATCC). Cells were subcultured, and antiproliferative assays were carried out, following the procedure that we have previously described [38]. Doxorubicin was included in all evaluations as a reference drug.

### 3.3. Computational Details

#### 3.3.1. Ligand Preparation

The 3D structure of each compound was prepared using Chem Draw Ultra version 12.0, as previously described [38]. Hydrophobicity index (cLogP), drug-likeness values, and toxicity risks were predicted through DataWarrior algorithms [68,69].

#### 3.3.2. In Silico ADME Prediction

Pharmacokinetics parameters were calculasted using QikProp (QP) version 4.3 of the Schrodinger suite based on Lipinski’s rule of five and its extensions, as previously described [38].

#### 3.3.3. Macromolecule Selection and Retrieval

The crystal structure of 14 selected proteins (Table S1), including growth factor receptors, transcription regulators, and enzymes (such as reductases, oxidases, and kinases) were retrieved from the Protein Data Bank [70]. They are overexpressed in some malignancies, including breast and colon adenocarcinoma, as described in the literature [50,51,52,53,55,56,57,61,62,63,71,72,73,74,75,76].

#### 3.3.4. Molecular Docking of Ligand–Protein Interaction

We resorted to virtual screening using Autodock Vina, a target-specific scoring method useful for virtual screening [77]. All chalcone–naphthoquinone/hydroquinone hybrids were docked into the set of proteins of known 3D structure to identify those potentially inhibited by these compounds. Both ligands and proteins were prepared using AutoDock Tools version 1.5.7 (ADT), as previously described [38,78,79]. Finally, the binding site and energies of each compound were predicted into each receptor using Autodock Vina [77]. The graphic analysis of the molecular coupling studies was performed using Visual Molecular Dynamics 1.9 (VMD) [80] and Discovery Studio Biovia [81].

## 4. Conclusions

In this study, a novel series of chalcone-1,4-naphthoquinones/benzohydroquinones (CNQs and CBHQs) was synthesized from the precursor 2-acetyl-5,8-dihydro-6-(4-methyl-3-pentenyl)-1,4-naphthohydroquinone. The synthesis process involved protecting the hydroxyl group at C-4 of the precursor to eliminate its acidic properties associated with the phenolic 4-OH group. This step was necessary to proceed with the Claisen–Schmidt condensation reaction. In general, CNQs **6** exhibited superior pIC_50_ values compared to those of CBHQs **4** and **5**, except for CBHQs **5e** and **5f**, which are diacetylated. This suggests that the coplanar structure of the naphthoquinone system and an appropriate level of lipophilicity, facilitating cell membrane penetration, favor the antineoplastic activity of the newly synthesized hybrid derivatives against both the MCF-7 and HT-29 cancer cell lines. It can also be inferred that the precursor derivatives of the 1,4-benzohydroquinone system, obtainable through the enzymatic hydrolysis of the respective diacetylated derivatives, are suitable due to the presence of hydroxyl groups, which enhance the binding energy when interacting with target proteins through hydrogen bonds. From a theoretical perspective, the binding energy of cancer-related proteins with CNQs and CBHQs was generally higher for kinases such as cMET, TRKA, and HER2, with ΔG*_bin_* values ranging from −11.1 to −7.2 kcal/mol. In this context, the synthesized cytotoxic hybrids (SCHs) are potential multi-kinase inhibitors and could serve as promising candidates for further research in the development of novel multi-target anticancer agents. However, experimental validation of the predictions and theoretical results for SCHs is essential before proceeding with acute toxicity and efficacy preclinical assays. Furthermore, the favorable predictions for physicochemical and pharmacokinetic parameters for most SCHs, aligning well with previous in vitro anti-proliferative results, underscore their potential as promising candidates for antineoplastic drug development.

## Figures and Tables

**Table 1:** In vitro cytotoxicity data for compounds **4a**–**f**, **5a**,**e**,**f**, **5′b**–**d,** and **6a**–**f** on MCF-7 breast adenocarcinoma and HT-29 colon adenocarcinoma cells.

Compounds	MCF-7		HT-29	
	IC_50_, µM ^[a]^	pIC_50_^[b]^	IC_50_, µM	pIC_50_
**4a**	>300	-	>300	-
**4b**	>300	-	>300	-
**4c**	>300	-	>300	-
**4d**	>300	-	>300	-
**4e**	>300	-	>300	-
**4f**	>300	-	>300	-
**5a**	nt	-	nt	-
**5′b**	>300	-	>300	-
**5′c**	>300	-	>300	-
**5′d**	>300	-	>300	-
**5e**	**10.9 ± 0.21**	**4.96**	**13.6 ± 0.14**	**4.87**
**5f**	**8.2 ± 0.42**	**5.09**	**6.0 ± 0.53**	**5.22**
**6a**	64.8 ± 1.09	4.19	110.5 ± 1.93	3.96
**6b**	**48.7 ± 0.76**	**4.31**	69.0 ± 0.21	4.16
**6c**	59.8 ± 1.83	4.22	183.3 ± 1.21	3.74
**6d**	>300	-	>300	-
**6e**	85.0 ± 0.93	4.07	101.7 ± 1.10	3.99
**6f**	nt	-	nt	-
**Doxorubicin**	**0.27 ± 0.08**	**6.57**	**4.07 ± 0.92**	**5.39**

^[a]^: IC_50_ (half-maximal inhibitory concentration) mean ± sd values; ^[b]^: pIC_50_ = −log IC_50_(M); in bold: significant cytotoxic effect IC_50_ < 50 µM; IC_50_ > 300 µM: compounds inactive; nt: not tested.

**Table 2:** Comparison (ΔG*_bin_*, kcal/mol) of synthesized cytotoxic hybrids and kinase inhibitors approved by the FDA for cancer.

Compounds	Target Proteins					
	EGFR	HER2	c-MET	TRKA	MEK1	TPK
**5e**	−7.2	−8.3	−9.8	−8.9	−9.2	−8.6
**5f**	−7.4	−8.6	−9.7	−9.1	−8.7	−8.2
**6a**	−10.6	−10.8	−10.3	−10.5	−10.5	−10.3
**6b**	−10.6	−10.2	−10.3	−10.7	−9.8	−10.3
**6c**	−11.1	−10.4	−10.6	−11.0	−10.2	−10.4
**6e**	−10.0	−10.2	−9.8	−9.6	−9.9	−9.9
P avge.	−9.53	−9.75	−10.08	−9.97	−9.72	−9.62
Erlotinib	−8.6	−8.0	−9.1	−8.8	−8.0	−8.5
Larotrectinib	−10.3	−8.8	−10.8	−11.0	−9.4	−9.5
Almonertinib	−7.7	−8.4	−10.4	−9.8	−8.8	−10.1
Anlotinib	−9.2	−10.3	−9.0	−10.7	−9.7	−9.0

Proteins with their respective (PDB) entries: EGFR: Epidermal growth factor receptor (5GTY); HER2: Epidermal growth factor receptor 2 (7JXH); c-MET: Mesenchymal-epithelial transition factor (3RHK); TRKA: Tropomyosin receptor kinase A (6PL2); MEK1: MAPK/ERK kinase (4AN3); TPK: Tyrosine-protein kinase (4EHZ).

**Table 3:** Predicted binding free energy values (ΔG*_bin_*, kcal/mol) and binding site contacts of synthesized cytotoxic hybrids with amino acids of c-MET, TRKA, and HER2.

Compounds	ΔG*_bin_*	H-Bonds and Hydrophobic Contacts in the Binding Site *
	**c-MET** (mean ΔG*_bin_* = −10.08 kcal/mol)	
**5e**	−9.8	Gly1028, Ile1084, Gly1085, His1088, Phe1089 *, Val1092/Ala1108, Lys1110, Val1155, Leu1157, **Asp1164**, Gly1163/Arg1208, Met1211, Phe1223 *, Ala 1226, **Arg1227**, Asp1231, Tyr1234 *
**5f**	−9.7	Ile1084, Gly1085, Arg1086, Gly1087, His1088, Phe1089 *, Val1092/Ala1108, Lys1110, Val1155, Leu1157, Gly1163, Asp1164, Asn1167 /Arg1208, Met1211, Phe1223 *, Ala1226, **Arg1227**, Asp1231, Tyr1234
**6a**	−10.3	Gly1087, His1088, Phe1089 *, Val1092 */Lys1110 *, Leu1157, Gly1163, Asp1164/Arg1208, Val1092, Met1211, Phe1223 *, Ala1226 *, **Arg1227**, Tyr1230, Asp1231, Tyr1234 *
**6b**	−10.3	His1088, Phe1089 *, Val1092 */Val1155, Leu1157, Gly1163, **Asp1164**/Arg1208, Met1211, Phe1223 *, Ala1226, **Arg1227**, Met1229, Tyr1230, Asp1231/Tyr1234 *, Tyr1235 *
**6c**	−10.6	Gly1087, His1088, Phe1089 *, Val1092 */Lys1110, Val1155, Leu1157, Gly1163, **Asp1164**, Asn1167/Arg1208, Met1211, Ala1226, Arg1227, Met1229, Tyr1230, Asp1231
**6e**	−9.8	Gly1085, His1088, Phe1089 *, Val1092/Ala1108, Lys1110, Val1155, Leu1157, Gly1163, Asp1164, Asn1167/Arg1208, Met1211, Phe1223 *, Ala1226, **Arg1227**, Asp1231, Tyr1234
	**TRKA** (mean ΔG*_bin_* = −9.97 kcal/mol)	
**5e**	−8.9	Leu516 *, Gly517, Glu518, Val524 */Ala542, Lys544, Glu560/Leu564, Val573 *, Phe589 *, Tyr591/Gly595, Asp596, Arg599, Leu657, Gly667/Asp668, Phe669 *, Arg673, Ile675, Tyr676
**5f**	−9.1	Leu516 *, Gly517, Gly519, Phe521 *, Gly522, Val524 */Ala542 *, Lys544, Glu560/Phe589, Glu590, Tyr591/**Met592**, Gly595, Asp596, Arg599, Leu657/Gly670, Ser672, Arg673, Ile675, Phe669 *
**6a**	−10.5	Leu516, Val524 */Lys544, Glu560/Leu564 *, Ile572, Val573, Phe589, Glu590, Tyr591/Gly595, Asp596, Leu657/Ile666, Gly667, Asp668, Phe669 *, Arg673
**6b**	−10.7	Leu516, Val524 */Ala542 *, Lys544,/Leu564 *, Ile572, Val573 *, Glu590, Phe589, Tyr591/Gly595, Asp596, Leu641, His648, Leu657/Gly667, Asp668, Phe669 *
**6c**	−11.0	Leu516, Val524 */Ala542 *, Lys544, Glu560/Leu564 *, Il572, Val573, Phe589, Glu590, Tyr591/Gly595, Asp596, Leu657/Ille666, Gly667, Asp668, Phe669 *
**6e**	−9.5	Leu516, Gly519, Phe521, Gly522, Val524 */Ala542, Lys544 *, Glu560/Phe589, Tyr591/Asp596, Arg599, Leu657 */Asp668, Phe669 *, Gly670, **Ser672**, **Arg673**, Ile675, Tyr676
	**HER2** (mean ΔG*_bin_* = −9.75 kcal/mol)	
**5e**	−8.3	Leu726 *, Val734 *, Ala751 */Lys753 *, Ile767, Glu770, Ala771, Met774/**Ser783**, Arg784, Leu785, Leu796, **Thr798**/Leu800, Met801, Gly804, Cys805, Leu852 */**Thr862**, Asp863, Phe864 *
**5f**	−8.6	Leu726, Val734 *, Ala751 */Ile752, Lys753, Ile767, Glu770, Ala771, Met774/Arg784, Leu785 *, Leu796 *, Thr798/Gln799, Leu800, Met801, Gly804, Cys805, Leu852 */Phe864, Gly865
**6a**	−10.8	Leu726, Gly727, Val734, Ala751 */Lys753, Ile767, Glu770, Ala771 */**Ser783**, Arg784, Leu785 *, Leu796 *, **Thr798**/Glu799, Leu800, Met801, Leu852 */Thr862, Asp863, Phe864
**6b**	−10.2	Leu726 *, Phe731, Val734 *, Ala751 */Lys753, Leu755, Ile767, Glu770, Met774,/Ser783, Arg784, Leu785 *, Leu796, **Thr798**/Gln799, Leu800, Met801, Gly804, Cys805, Leu852 */Thr862, Asp863, Phe864 *, Gly865
**6c**	−10.4	Leu726 *, Phe731, Val734 *, Ala751 */Lys753, Leu755, Ile767, Glu770, Met774/Ser783, Arg784, Leu785 *, Leu796, **Thr798**/Gln799, Leu800, Met801, Gly804, Leu852 */Thr862, Asp863, Phe864 *, Gly865
**6e**	−10.2	Phe731/Glu770, Met774/**Ser783**, Arg784, **Thr798**/Gln799, Met801, Gly804/Thr862, Asp863, Gly865, Arg849

Bolded names correspond to the amino acids involved in H-bonds with the corresponding synthesized cytotoxic hybrids. Partially interacting peptide sequences are separated by/and differentiated with colors to facilitate comparisons between similar interactions. Residues with * correspond to amino acids that interact with the ligand by any type of Pi interaction.

**Table 4:** Binding site contacts of compound **6c**, ligand, and drug into c-MET, TRKA, and HER2.

Compounds	ΔG*_bin_* (kcal/mol)	H-Bonds and Hydrophobic Contacts in the Binding Site
		c-MET
**6c**	−10.6	Gly1087, His1088, Phe1089, Val1092, Lys1110, Val1155, Leu1157, Gly1163, **Asp1164**, Asn1167, Arg1208, Met1211, Ala1226, Arg1227, Met1229, Tyr1230, Asp1231
**Ligand 1 ^[a]^**	−14.6	Ile1084, Gly1085, Phe1089, Val1092, Ala1108, Lys1110, Leu1140, Leu1157, Tyr1159, **Met1158**, **Met1160**, Gly1163, Met1211, Phe1223, Ala1226, Arg1227
**Erlotinib ^[b]^**	−9.1	Phe1089, Val1092, Ala1108, Lys1110, Val1155, Leu1157, Gly1163, Asp1164, Asn1167, Arg1208, Met1211, Arg1221, Phe1223, Ala1226, **Arg1227**, Asp1231, Tyr1234
		**TRKA**
**6c**	−11.0	Leu516, Val524, Ala542, Lys544, Glu560, Leu564, Ile572, Val573, Phe589, Glu590, Tyr591, Gly595, Asp596, Leu657, Ille666, Gly667, Asp668, Phe669
**Ligand 2 ^[a]^**	−14.2	Leu516, Val524, Ala542, Lys544, Arg559, **Glu560**, Leu563, Leu564, Leu567, Ile572, Val573, Phe589, Glu590, Tyr591, **Met592**, Leu641, Phe646, His648, Leu657, Ile666, Gly667, **Asp668**, Phe669
**Larotrectinib ^[b]^**	−11.0	Gly517, Glu518, Gly519, Phe521, Gly522, Val524, Ala542, Lys544, Glu560, Val573, Met587, Phe589, Leu657, Gly667, Asp668, Phe669, Gly670, **Ser672**, Arg673
		**HER2**
**6c**	−10.2	Leu726, Phe731, Val734, Ala751, Lys753, Leu755, Ile767, Glu770, Met774, Ser783, Arg784, Leu785, Leu796, **Thr798**, Gln799, Leu800, Met801, Gly804, Leu852, Thr862, Asp863, Phe864, Gly865,
**Ligand 3 ^[a]^**	−14.5	Leu726, Val734, Ala751, Lys753, Met774, **Ser783**, Arg784, Leu785, Leu796, Thr798, Gln799, Leu800, Met801, Pro802, Gly804, **Cys805**, Leu807, Asp808, Arg849, Leu852, Thr862, Asp863, Phe864
**Erlotinib ^[b]^**	−8.0	Leu726, Val734, Ala751, Lys753, Leu785, Leu796, Thr798, Gn799, Leu800, Met801, Gly804, Cys805, Asn850, Leu852, **Thr862**, Asp863, Phe864

^[a]^ Ligand 1, 2 and 3 respectively correspond to 1-[(3R,4R)-4-(1H-indol-3-yl)-2,5-dioxopyrrolidin-3-yl]pyrrolo [3,2,1-ij]quinolinium, N-(3-tert-butyl-1-phenyl-1H-pyrazol-5-yl)-2-{[1-(4-hydroxyphenyl)-1H-tetrazol-5-yl]sulfanyl}acetamide and (2E)-N-[3-cyano-7-ethoxy-4-({3-methyl-4-[([1,2,4]triazolo [1,5-a]pyridin-7-yl)oxy]phenyl}amino)quinolin-6-yl]-4-(dimethylamino)but-2-enamide, respectively. 3D structures of ligands 1, 2, and 3 were extracted from the Protein Data Bank, using the PDB IDs 3RHK, 6PL2, and 7JXH, respectively. ^[b]^ These compounds are drugs that act as inhibitors of biological targets [65,66,67]. Words in blue, green, and red correspond to amino acids shared by **6c** and ligand, **6c** and drug, and **6c**, ligand, and drug, respectively. Bolded names correspond to the amino acids involved in H-bond’s 6c enzyme.

**Table 5:** Comparative toxicity risks ^a^ predicted and *drug-likeness* scores ^a^ for compounds **5e**,**f**, and **6a**–**c**,**e**.

Compound	M	T	I	R	*Drug-Likeness*
**5e**	*N*	*n*	***h***	*n*	−0.14
**5f**	*N*	*n*	***h***	*n*	**2.15**
**6a**	*N*	*n*	*n*	*n*	−5.81
**6b**	*N*	*n*	***h***	***l***	−2.28
**6c**	*N*	*n*	*n*	*n*	−4.04
**6e**	*N*	*n*	*n*	*n*	−1.47

^a^: Predicted through DataWarrior algorithm, M: Mutagenic, T: Tumorigenic, I: Irritant, R: Reproductive effective; levels: none (*n*), low (*l*), and high (*h*).

## Data Availability

Not applicable.

## Supplementary Materials

The following supporting information can be downloaded at: https://www.mdpi.com/article/10.3390/molecules28207172/s1, **Figure S1**: ^1^H NMR spectrum of compound **2**. Figure S2: ^13^C NMR spectrum of compound **2.** Figure S3: ^1^H NMR spectrum of compound **3a**. Figure S4: ^1^H NMR spectrum of compound **3b**. Figure S5: ^1^H NMR spectrum of compound **3c**. Figure S6: ^1^H NMR spectrum of compound **3d**. Figure S7: ^1^H NMR spectrum of compound **3e**. Figure S8: ^1^H NMR spectrum of compound **3f**. Figure S9: ^1^H NMR spectrum of compound **4a**. Figure S10: ^1^H NMR spectrum of compound **4b**. Figure S11: ^1^H NMR spectrum of compound **4c**. Figure S12: ^1^H NMR spectrum of compound **4d**. Figure S13: ^1^H NMR spectrum of compound **4e**. Figure S14: ^1^H NMR spectrum of compound **4f**. Figure S15: ^13^C NMR spectrum of compound **3a**. Figure S16: ^13^C NMR spectrum of compound **3b**. Figure S17: ^13^C NMR spectrum of compound **3c**. Figure S18: ^13^C NMR spectrum of compound **3d**. Figure S19: ^13^C NMR spectrum of compound **3e**. Figure S20: ^13^C NMR spectrum of compound **3f**. Figure S21: ^13^C NMR spectrum of compound **4a**. Figure S22: ^13^C NMR spectrum of compound **4b**. Figure S23: ^13^C NMR spectrum of compound **4c**. Figure S24: ^13^C NMR spectrum of compound **4d**. Figure S25: ^13^C NMR spectrum of compound **4e**. Figure S26: ^13^C NMR spectrum of compound **4f**. Figure S27: ^1^H NMR spectrum of compound **5′b**. Figure S28: ^1^H NMR spectrum of compound **5′c**. Figure S29: ^1^H NMR spectrum of compound **5′d**. Figure S30: ^1^H NMR spectrum of compound **5e**. Figure S31: ^1^H NMR spectrum of compound **5f**. Figure S32: ^1^H NMR spectrum of compound **6a**. Figure S33: ^1^H NMR spectrum of compound **6b**. Figure S34: ^1^H NMR spectrum of compound **6c**. Figure S35: ^1^H NMR spectrum of compound **6d**. Figure S36: ^1^H NMR spectrum of compound **6e**. Figure S37: ^13^C NMR spectrum of compound **5′b**. Figure S38: ^13^C NMR spectrum of compound **5′c**. Figure S39: ^13^C NMR spectrum of compound **5′d**. Figure S40: ^13^C NMR spectrum of compound **5e**. Figure S41: ^13^C NMR spectrum of compound **5f**. Figure S42: ^13^C NMR spectrum of compound **6a**. Figure S43: ^13^C NMR spectrum of compound **6b**. Figure S44: ^13^C NMR spectrum of compound **6c**. Figure S45: ^13^C NMR spectrum of compound **6d**. Figure S46: ^13^C NMR spectrum of compound **6e**. Figure S47: Plotted 2D maps of H-bonds and hydrophobic interactions of CNQ **5e** and **5f** with c-MET residues. Figure S48: Plot 2 D-maps of H-bonds and hydrophobic interactions of **5e**, **5f**, and **6a**–**c**,**e** with TRKA residues. Figure S49: Plotted 2D maps of H-bonds and hydrophobic interactions of CNQ **5e**, **5f**, and **6a**–**c**,**e** with HER2 residues. Figure S50: A: Overlapping of the docking poses for CNQ hybrid **6c** (green), ligand 2 (yellow), and larotrectinib (grey) into TRKA. Superimposition of the docking poses for B: **6c** and ligand 1, and C: **6c** and larotrectinib. Figure S51: A: Overlapping of the docking poses for CNQ hybrid **6c** (green), ligand 2 (yellow), and erlotinib (grey) into HER2. Superimposition of the docking poses for B: **6c** and ligand 1, and C: **6c** and erlotinib. Table S1: Predicted binding free energy values (∆G*_bin_* kcal/mol) of synthesized cytotoxic hybrids with selected proteins overexpressed in cancer. Table S2: Comparison (ΔG*_bin_*, kcal/mol) of chalcones-1,4-Naphthoquinone/Hydroquinone hybrids with kinase proteins overexpressed in cancer. Table S3: Predicted binding free energy values (ΔG*_bin_*, kcal/mol) and binding site contacts of chalcones hybrids with amino acids of MEK1, TPK, and EGFR. Table S4: Physical and pharmacokinetic data predicted by QikPropa extensions for compounds **5e**, **5f**, and **6a**–**c**,**e**. Table S5: Evaluation parameters of Lipinski’s rule of five and its extensions for compounds **5e**, **5f**, and **6a**–**c**,**e**.
