# Supplementary material for: 2-Acetyl-5,8-dihydro-6-(4-methyl-3-pentenyl)-1,4-naphthohydroquinone-Derived Chalcones as Potential Anticancer Agents
Source: Molecules. 2023 Oct 19;28(20):7172. doi: 10.3390/molecules28207172 (PMC10609043; doi:10.3390/molecules28207172)
Supplement: Supplementary file 1 [file molecules-28-07172-s001.zip › molecules-2627251-Supplementary figures.pdf]

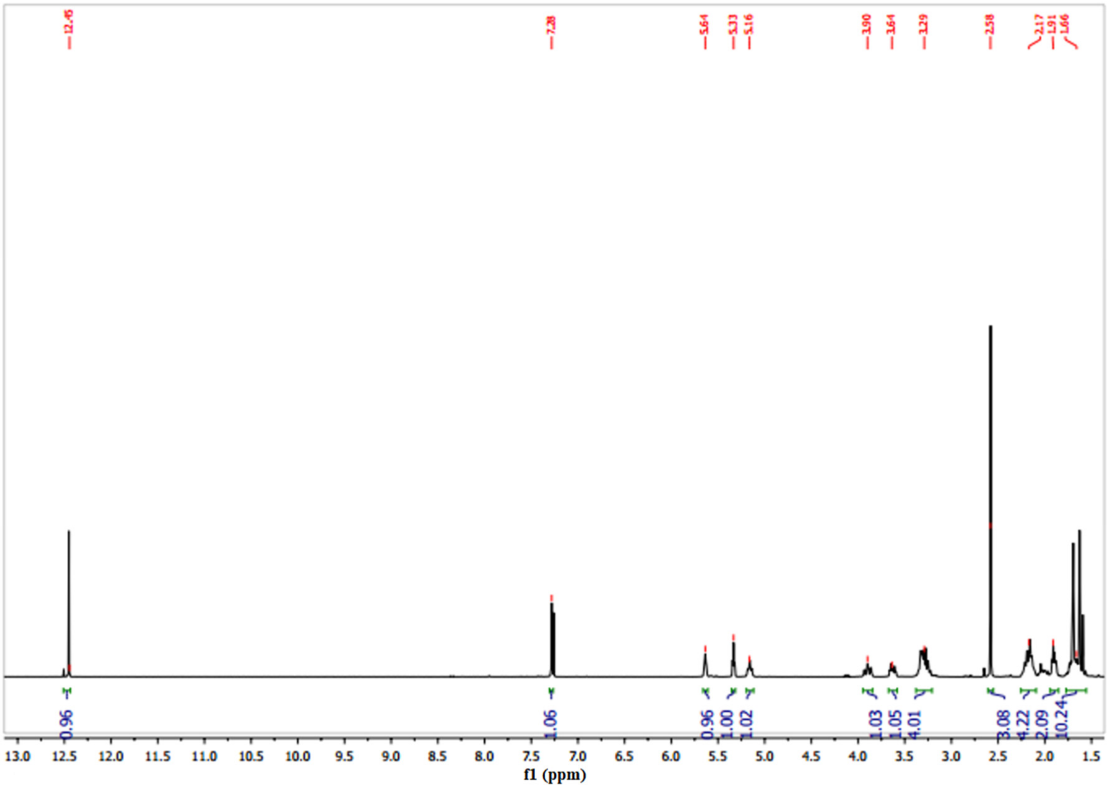

Figure S1. ^{1}H NMR spectrum of compound **2**.

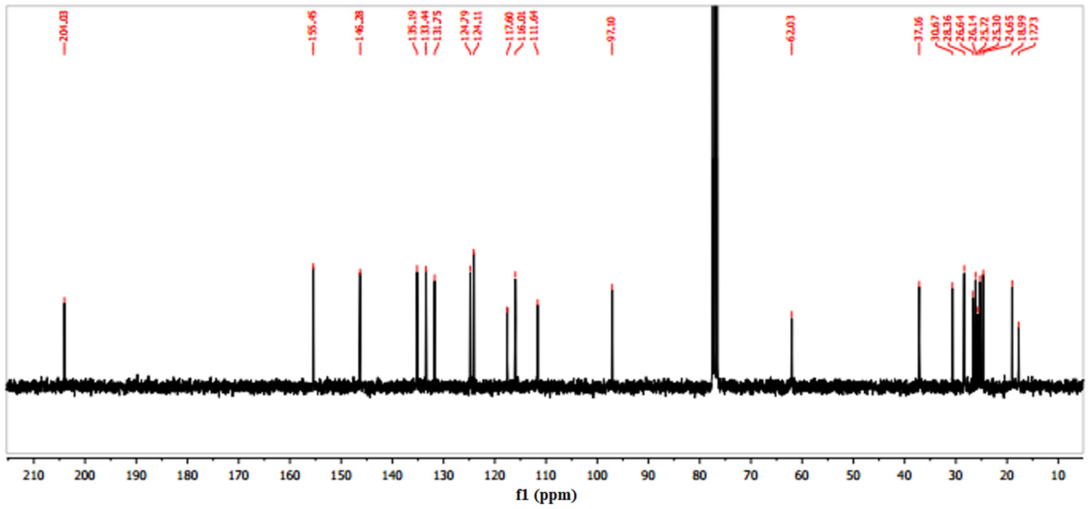

Figure S2. ^{13}C NMR spectrum of compound **2**.

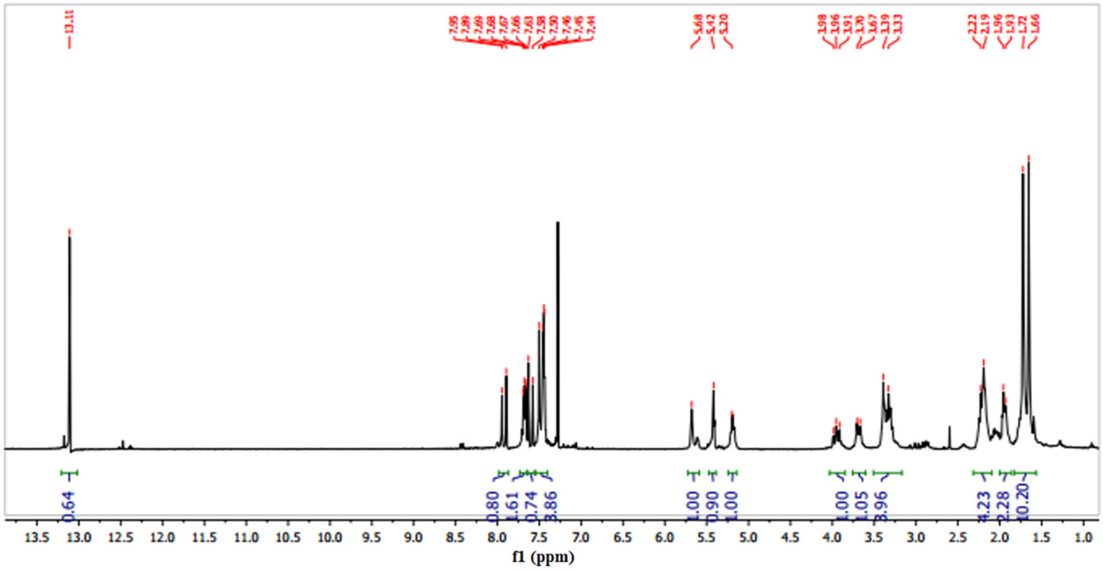

Figure S3. ^{1}H NMR spectrum of compound **3a**.

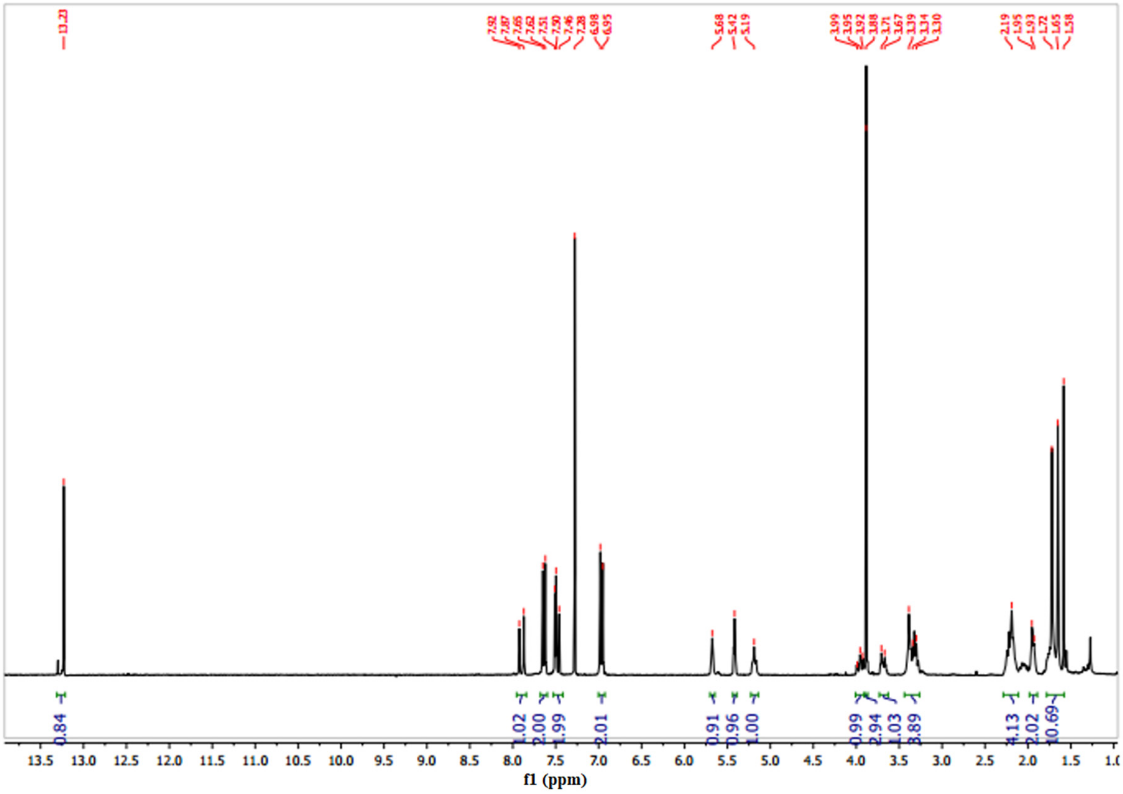

Figure S4. ^{1}H NMR spectrum of compound **3b**.

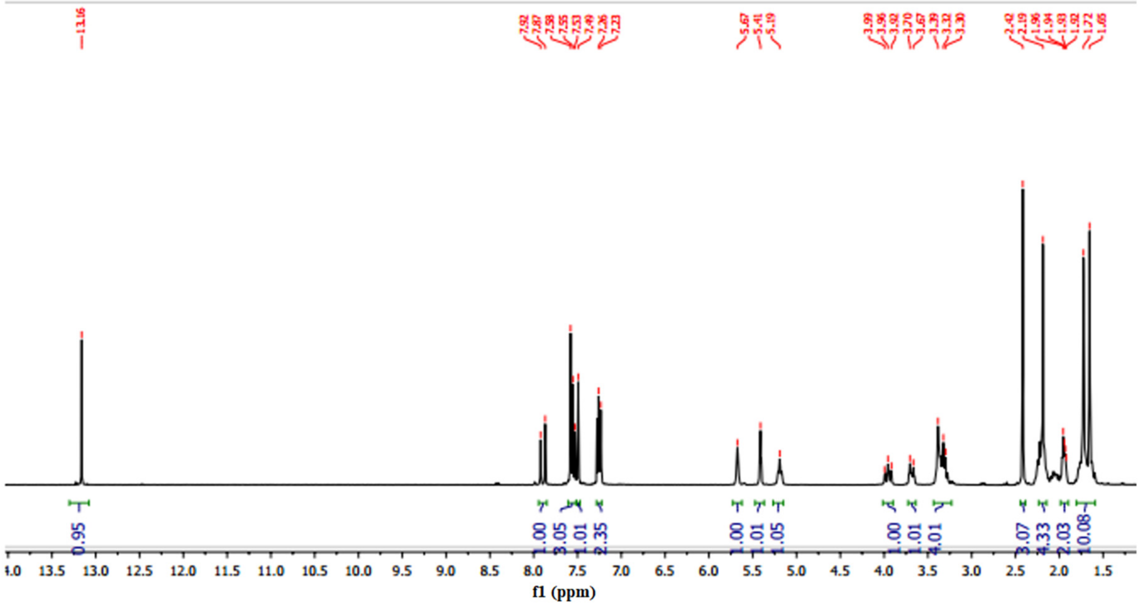

Figure S5. ^{1}H NMR spectrum of compound **3c**.

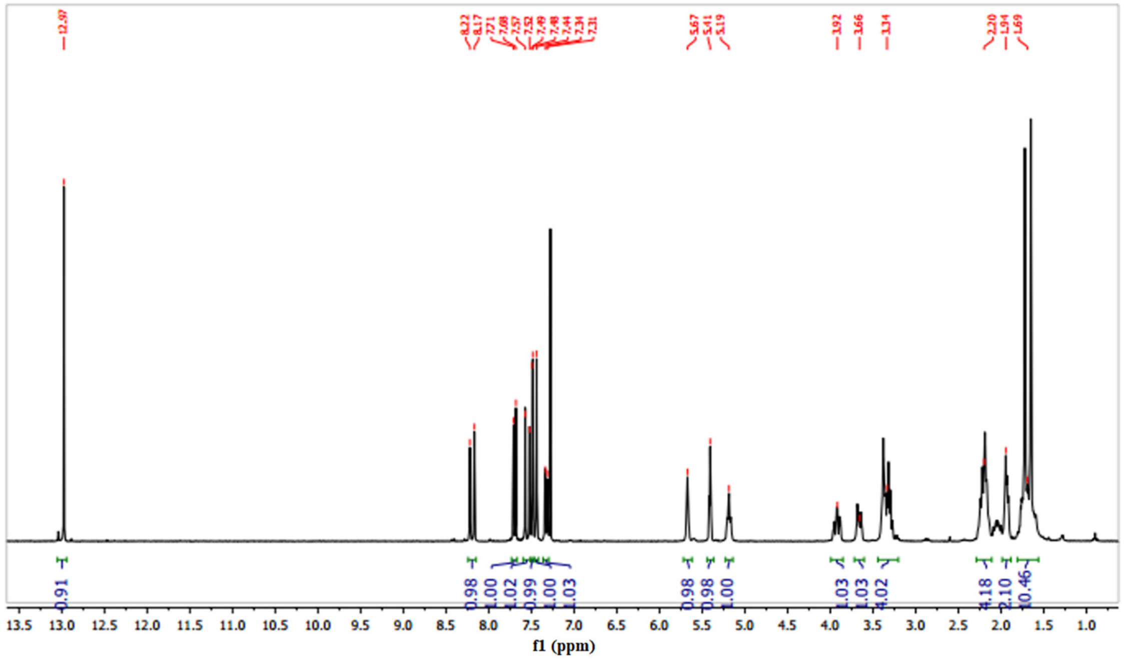

Figure S6. ^{1}H NMR spectrum of compound **3d**.

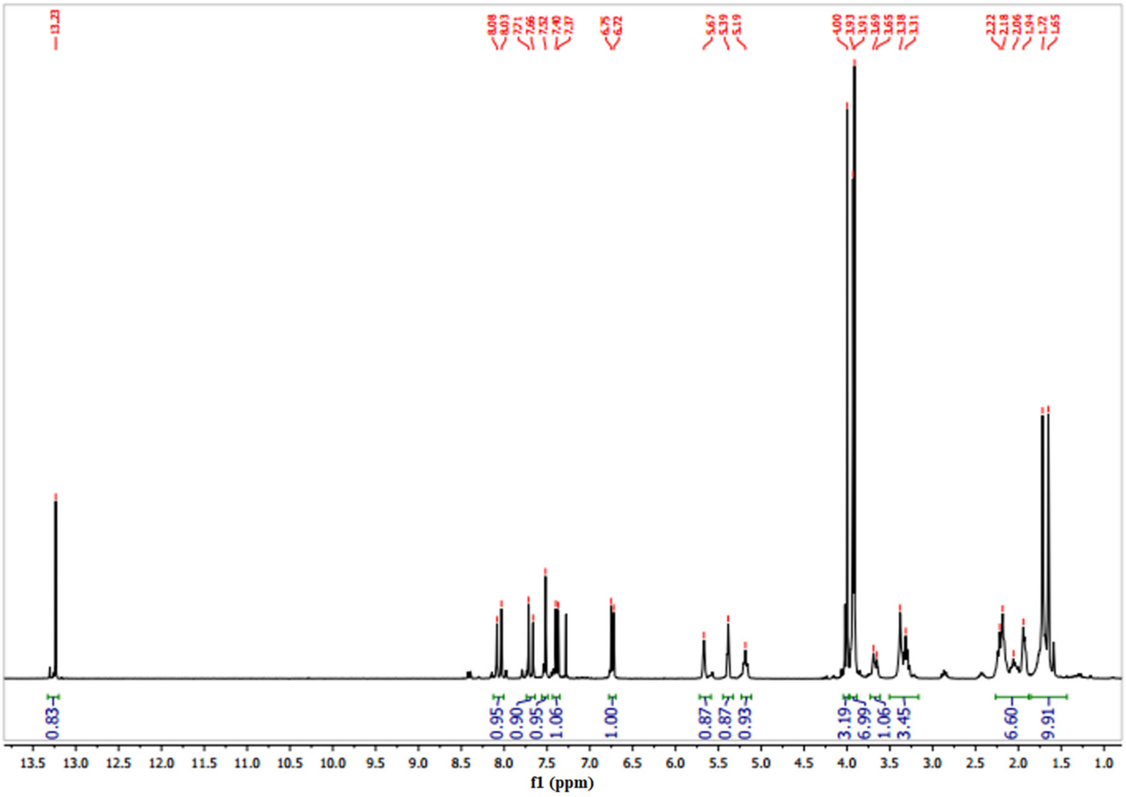

Figure S7. ^{1}H NMR spectrum of compound **3e**.

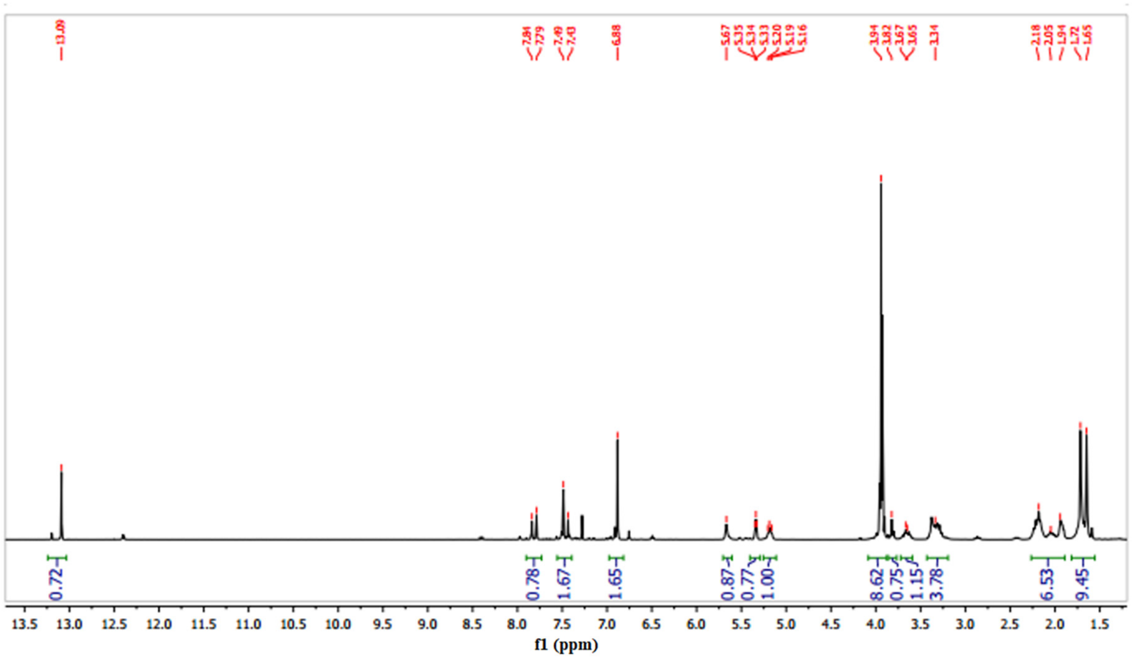

Figure S8. ^{1}H NMR spectrum of compound **3f**.

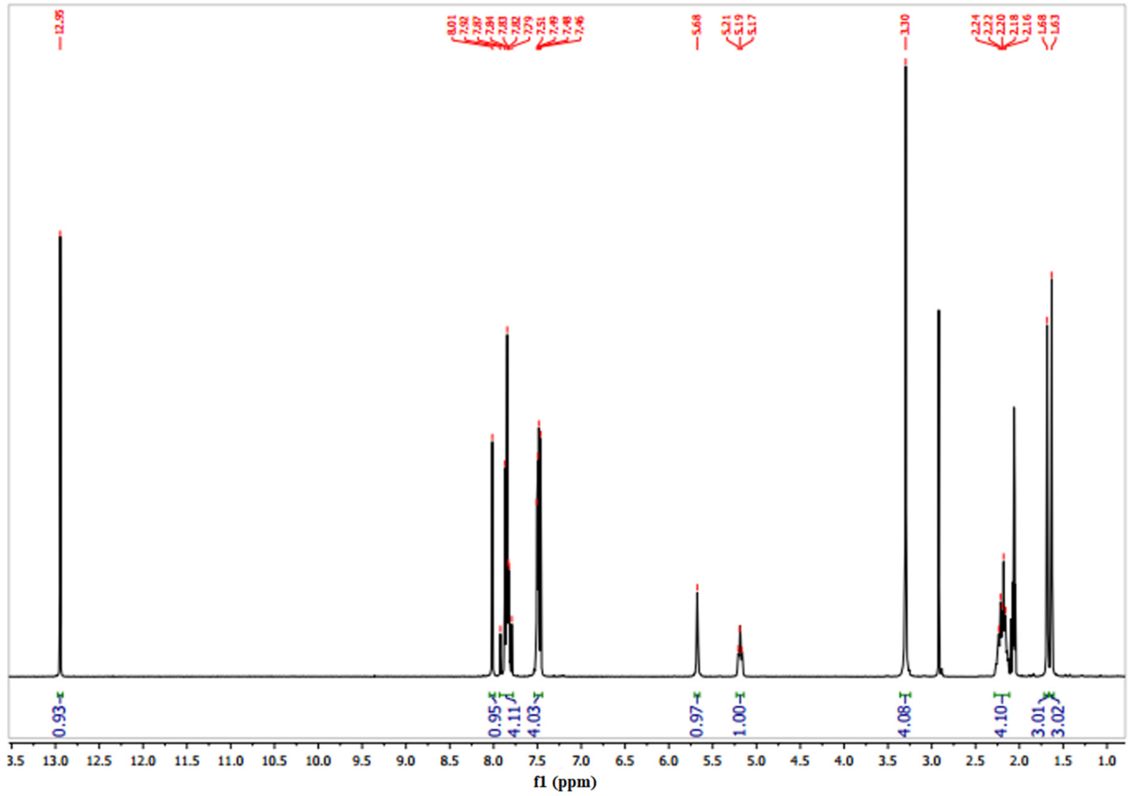

Figure S9. ^{1}H NMR spectrum of compound **4a**.

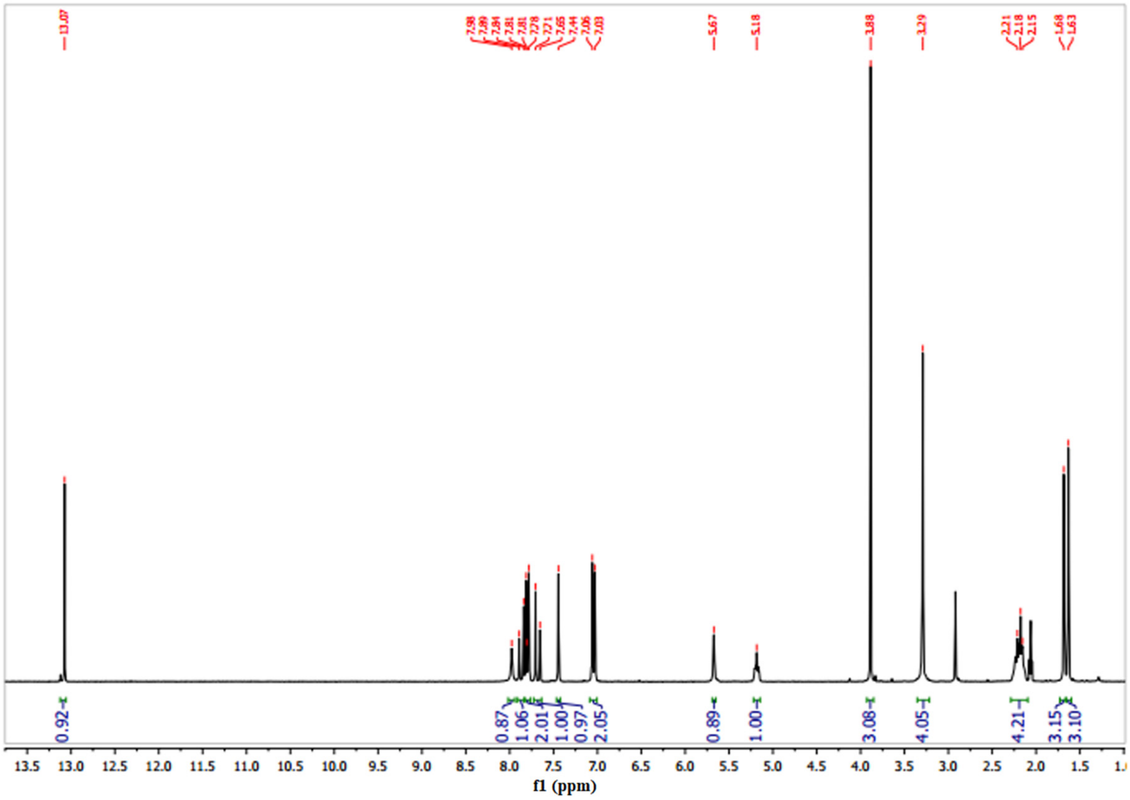

Figure S10. ¹H NMR spectrum of compound **4b**.

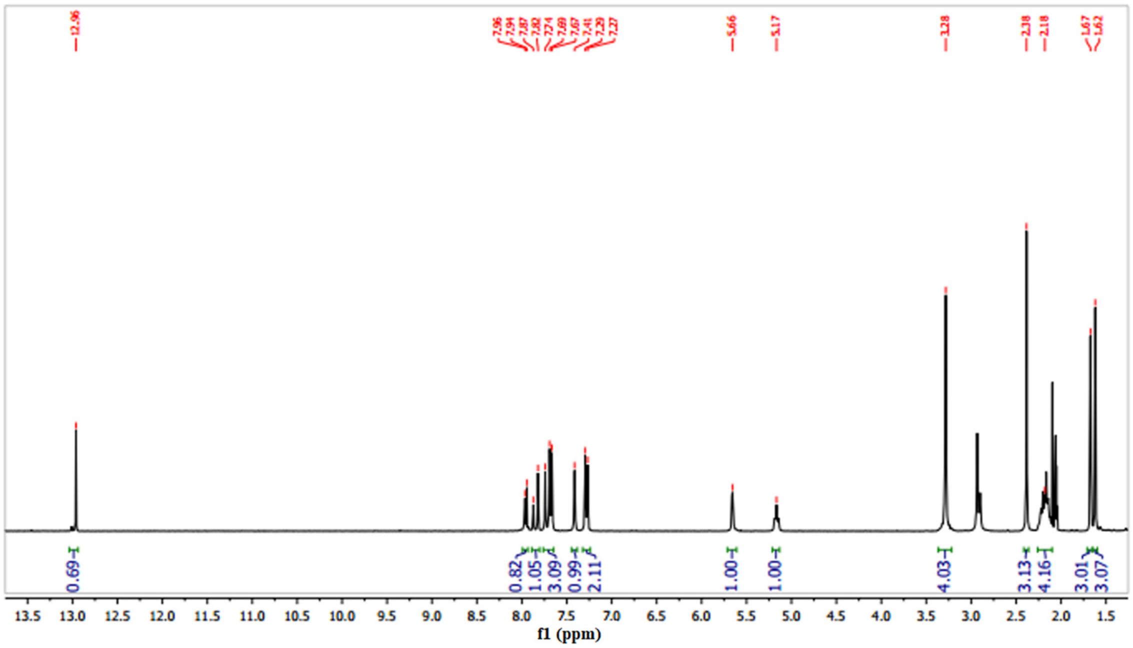

Figure S11. ^{1}H NMR spectrum of compound **4c**.

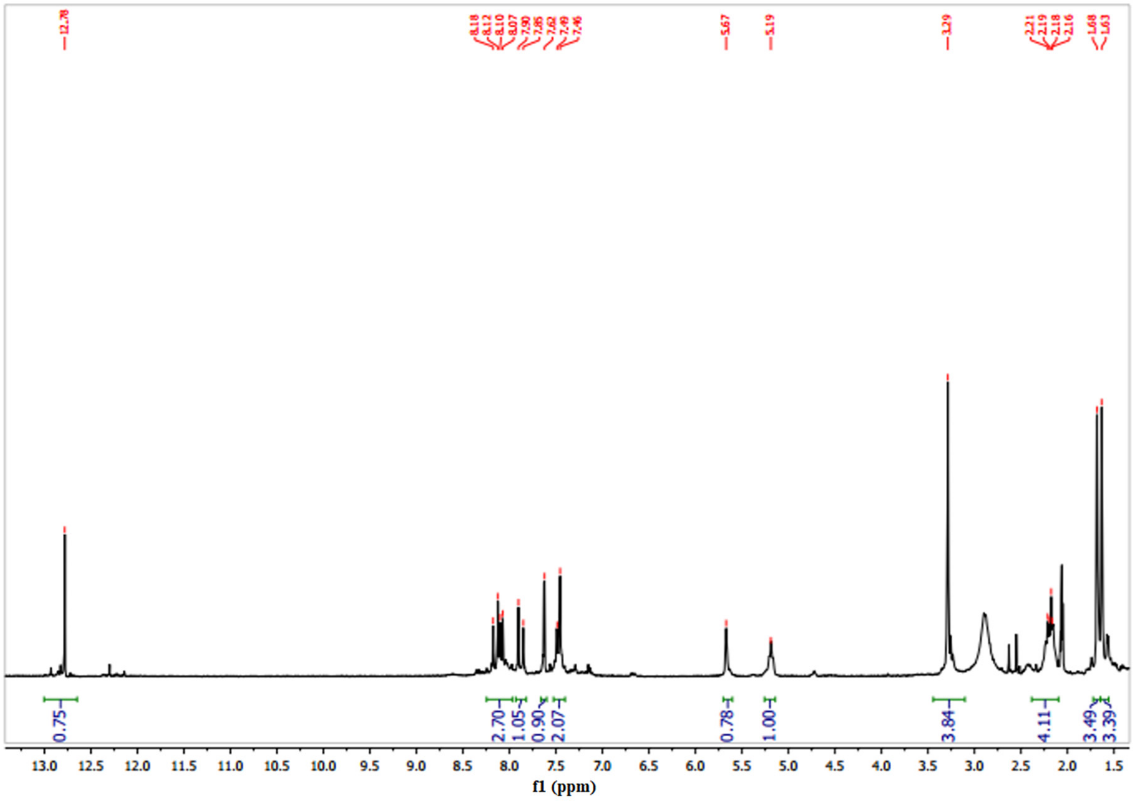

Figure S12. ^{1}H NMR spectrum of compound **4d**.

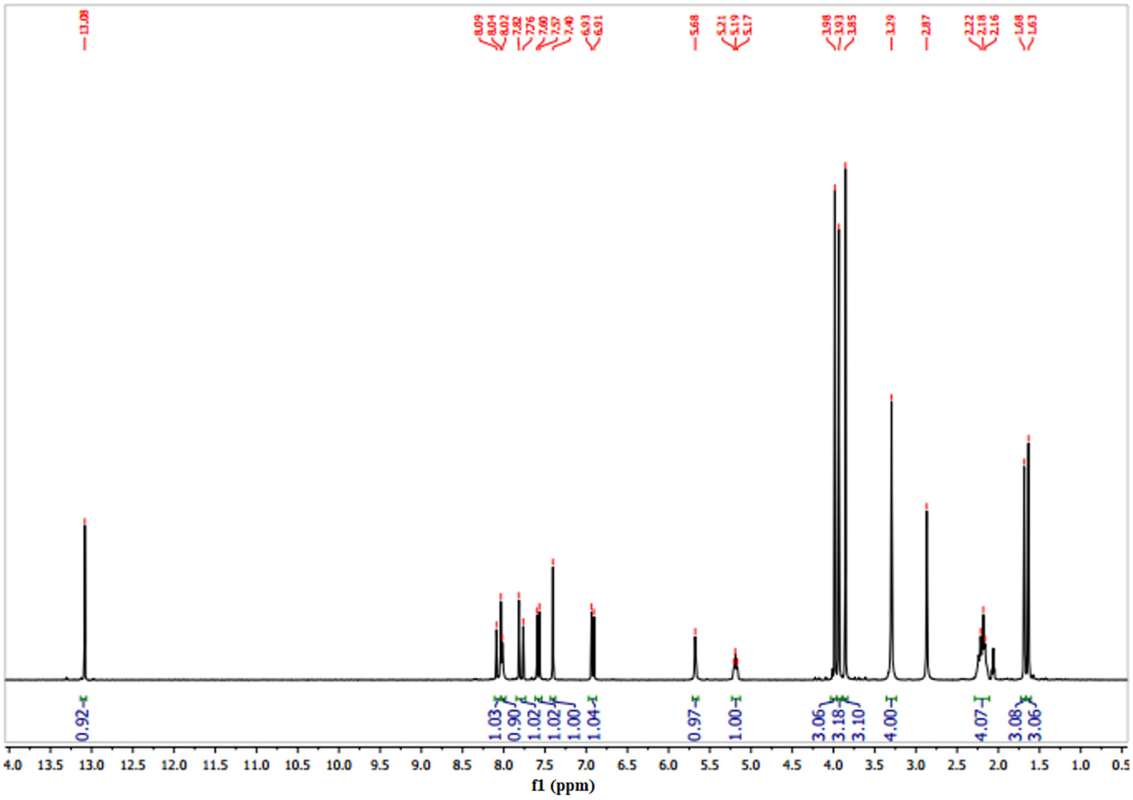

Figure S13. ^{1}H NMR spectrum of compound **4e**.

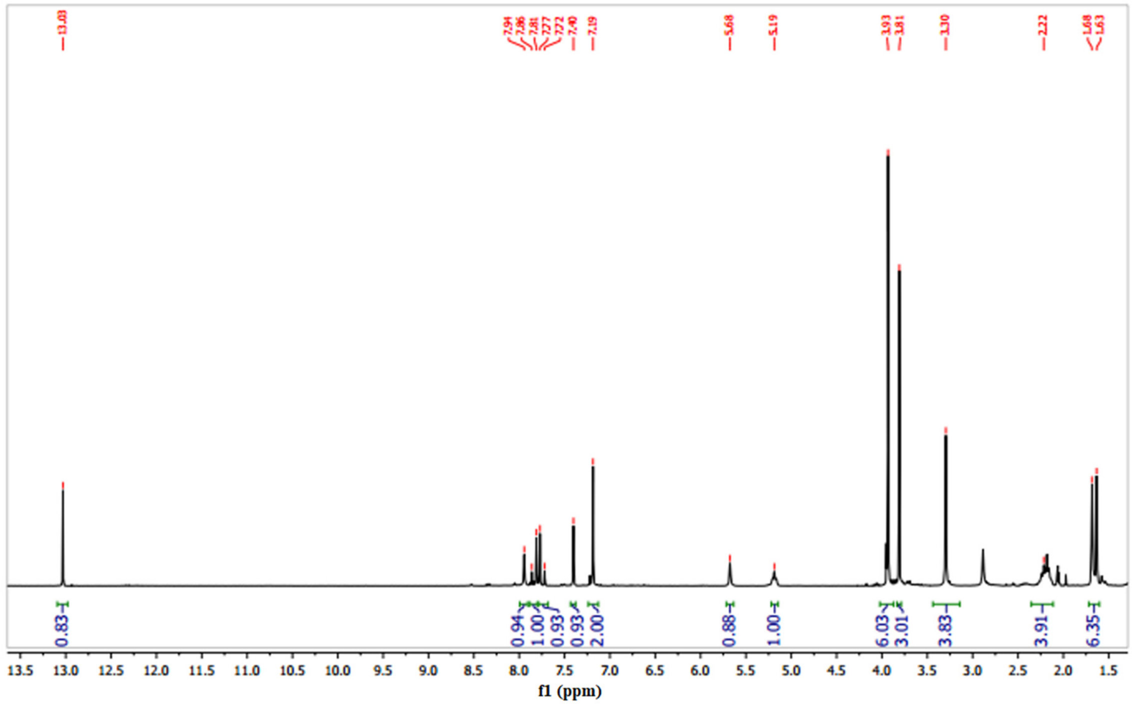

Figure S14. ^1H NMR spectrum of compound **4f**.

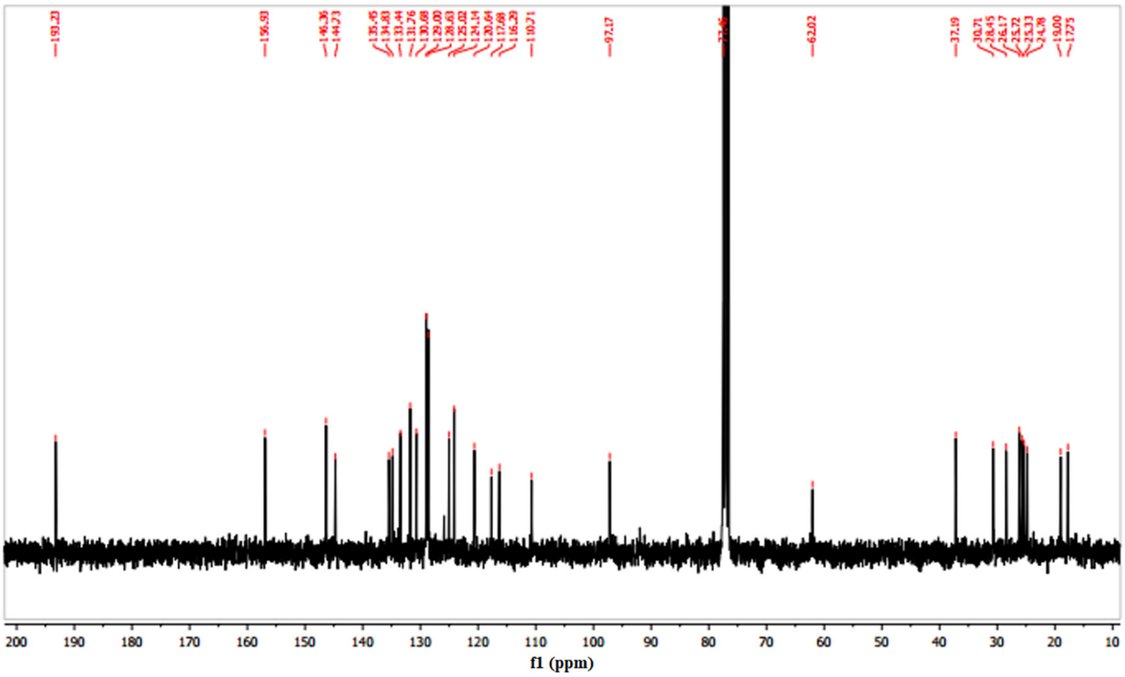

Figure S15. ^{13}C NMR spectrum of compound **3a**.

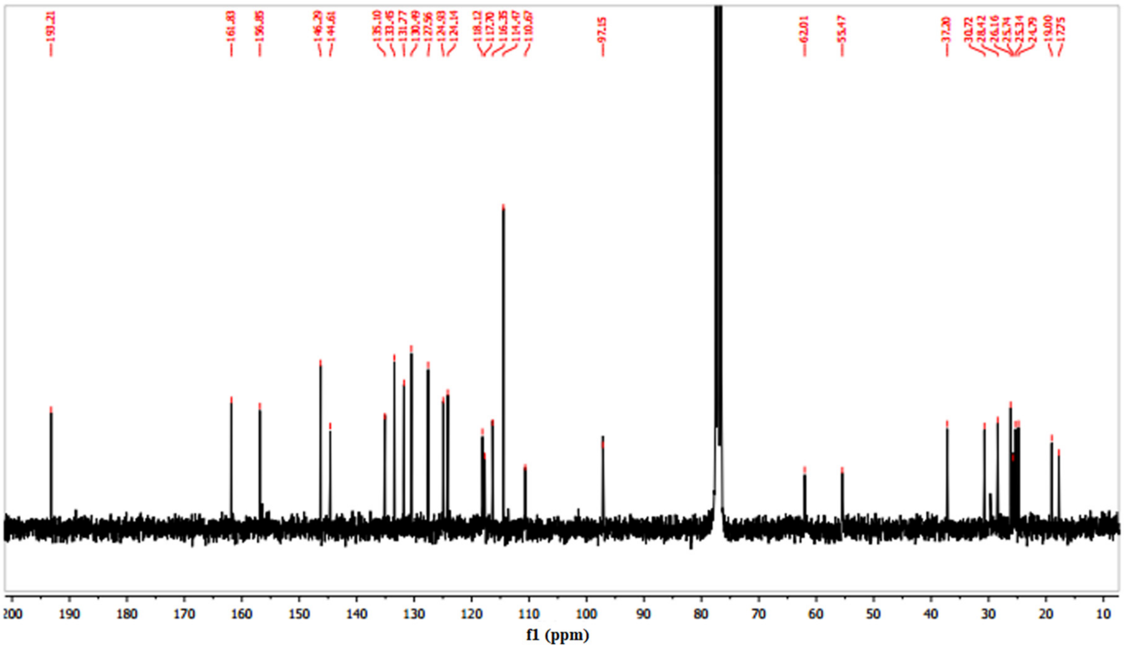

Figure S16. ^{13}C NMR spectrum of compound **3b**.

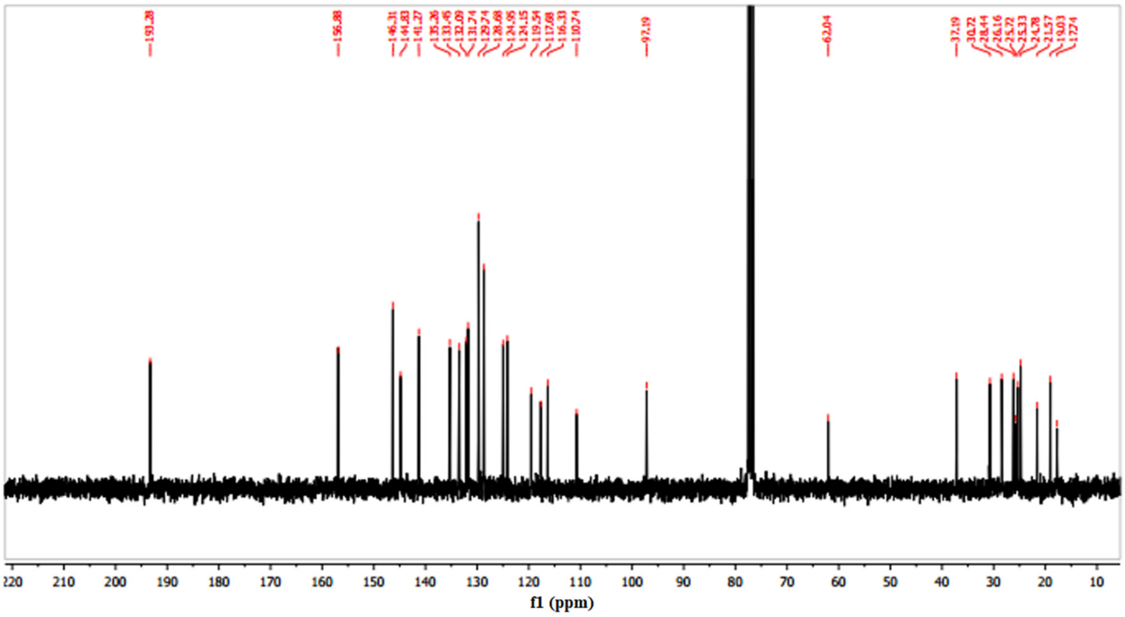

Figure S17. ^{13}C NMR spectrum of compound **3c**.

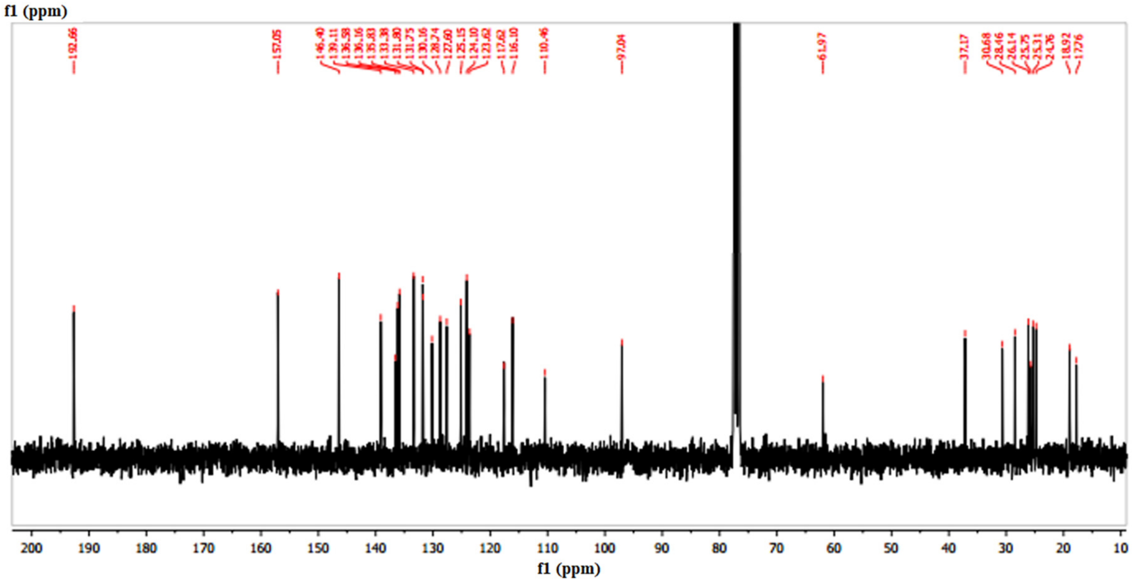

Figure S18. ^{13}C NMR spectrum of compound **3d**.

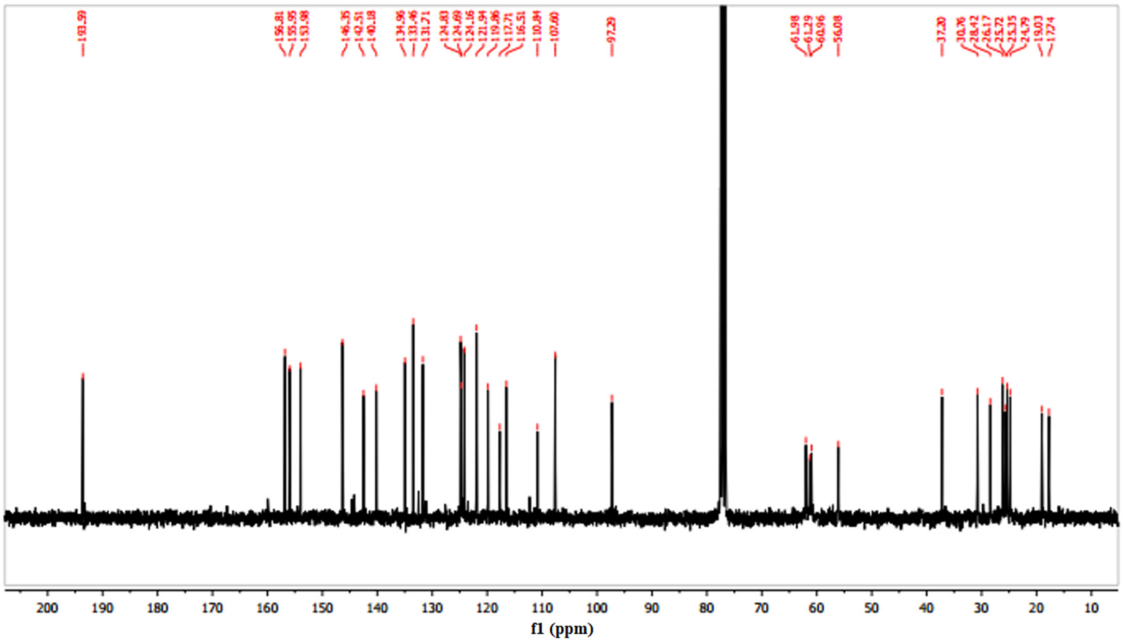

Figure S19. ^{13}C NMR spectrum of compound **3e**.

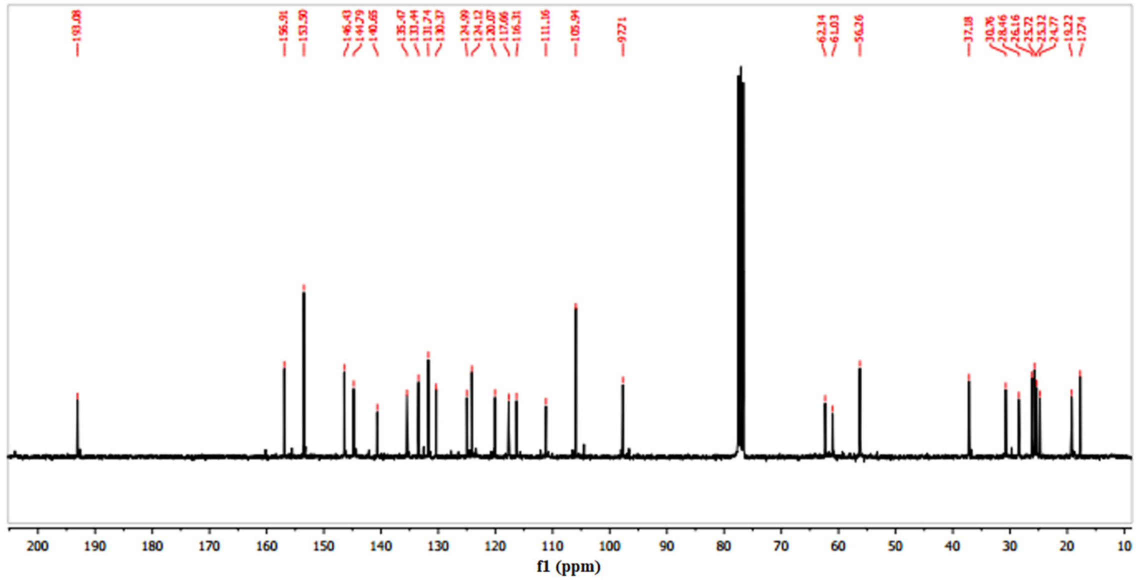

Figure S20. ^{13}C NMR spectrum of compound **3f**.

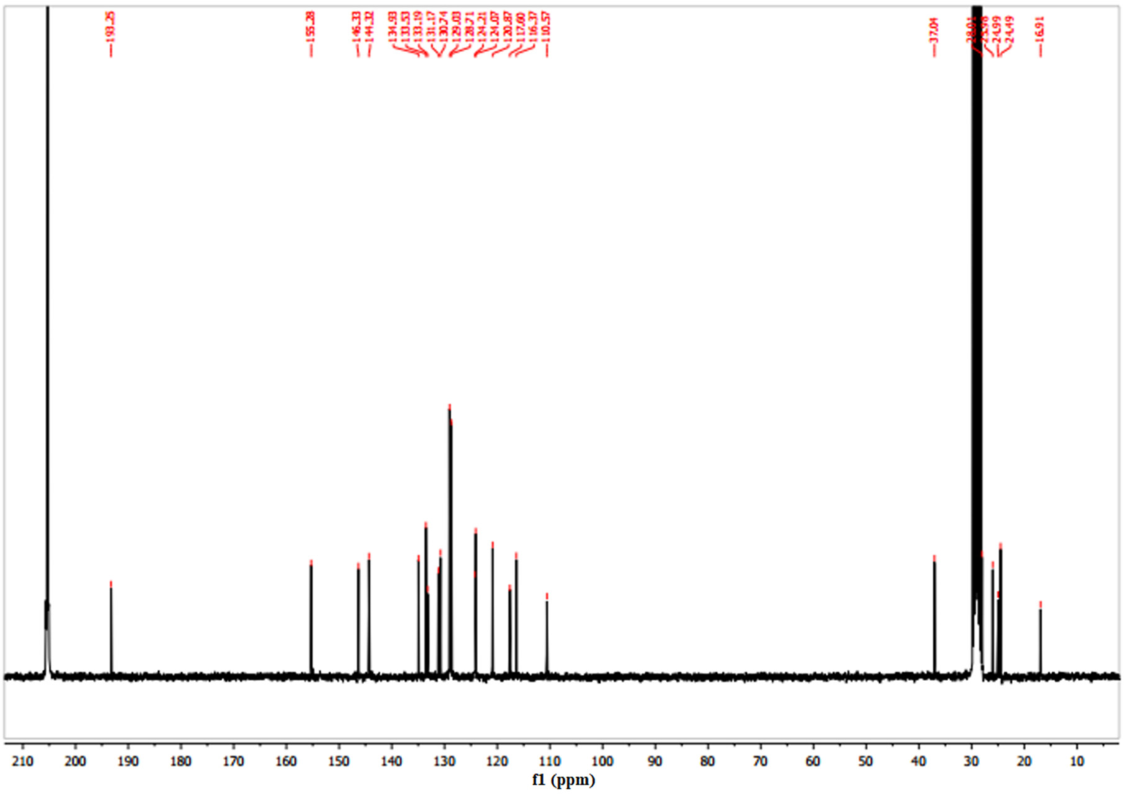

Figure S21. ^{13}C NMR spectrum of compound **4a**.

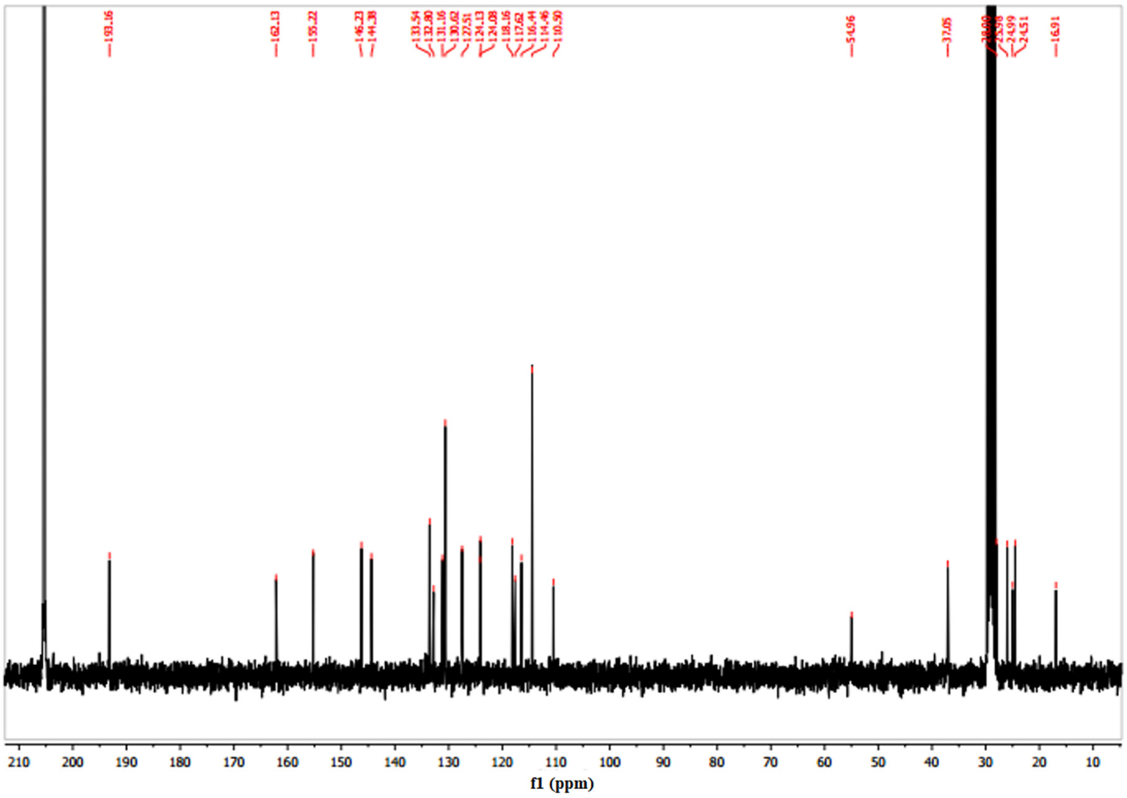

Figure S22. ^{13}C NMR spectrum of compound **4b**.

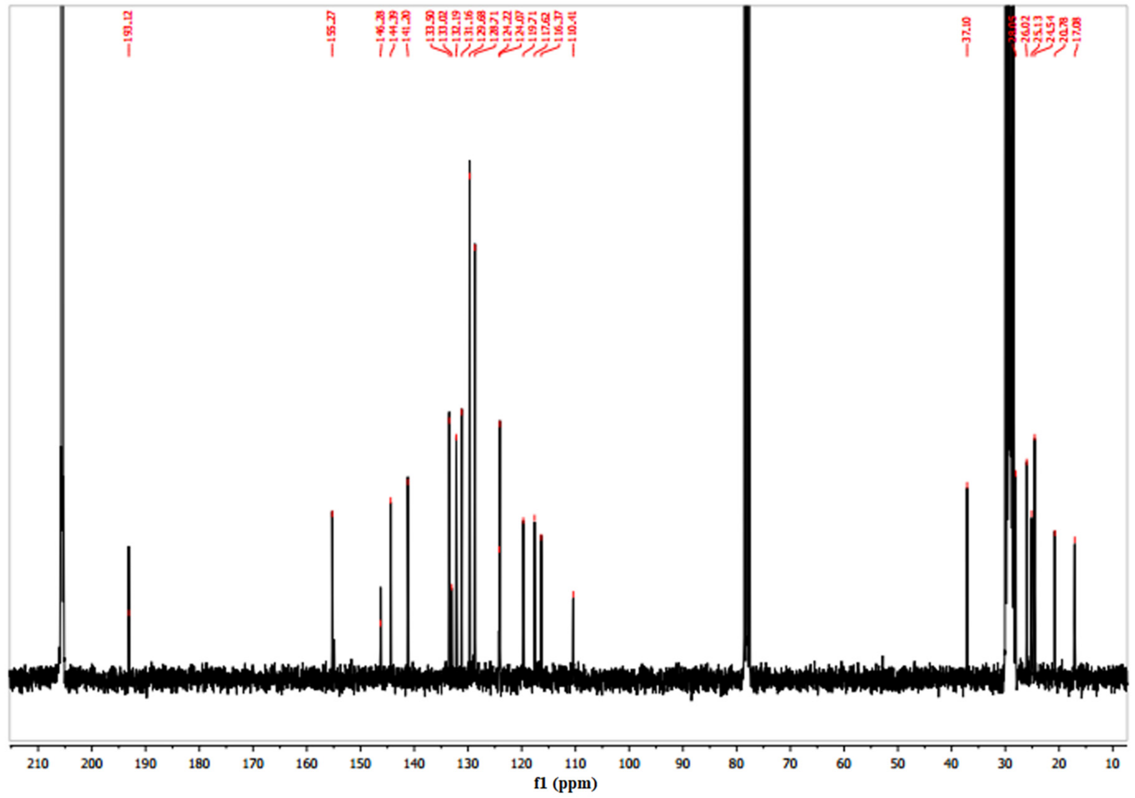

Figure S23. ^{13}C NMR spectrum of compound **4c**.

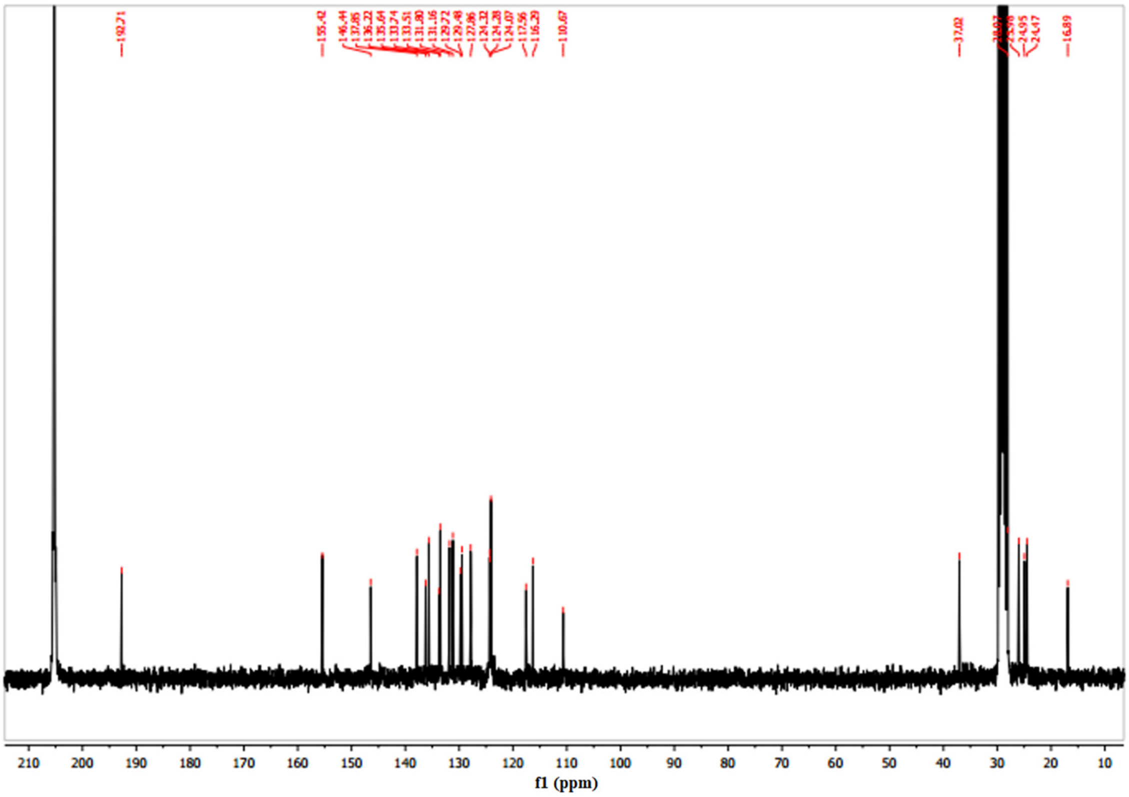

Figure S24. ^{13}C NMR spectrum of compound **4d**.

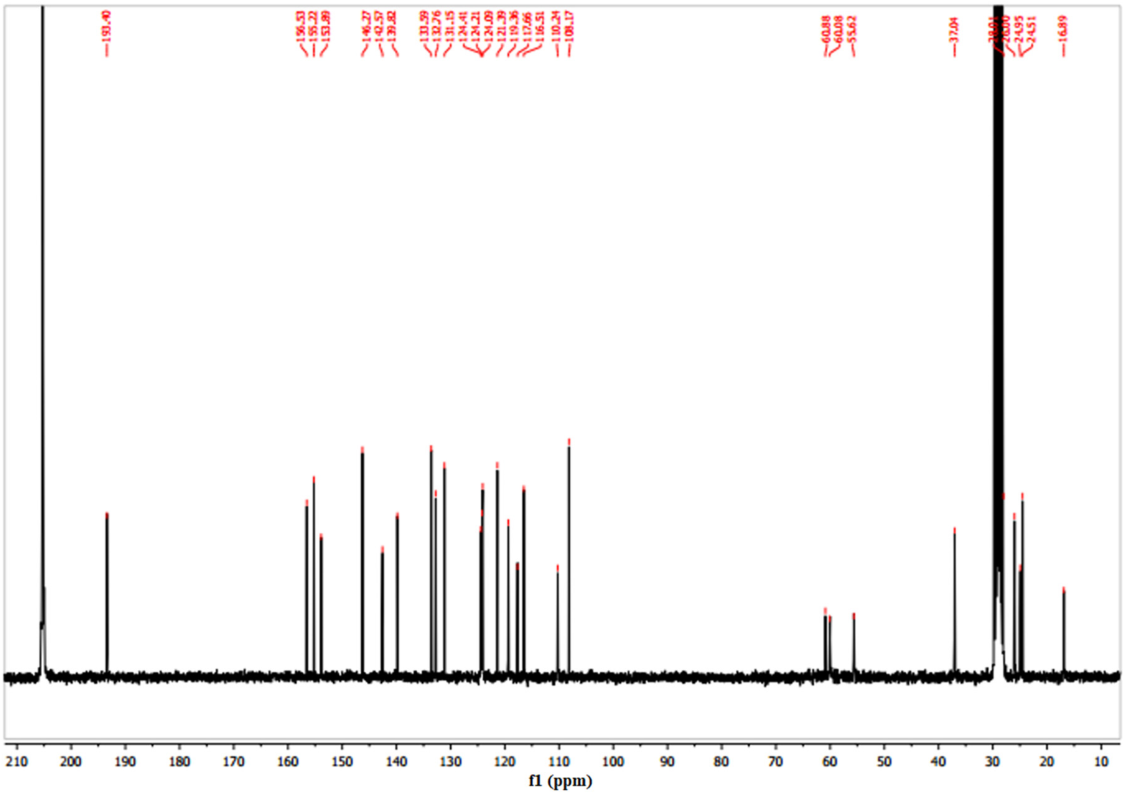

Figure S25. ^{13}C NMR spectrum of compound **4e**.

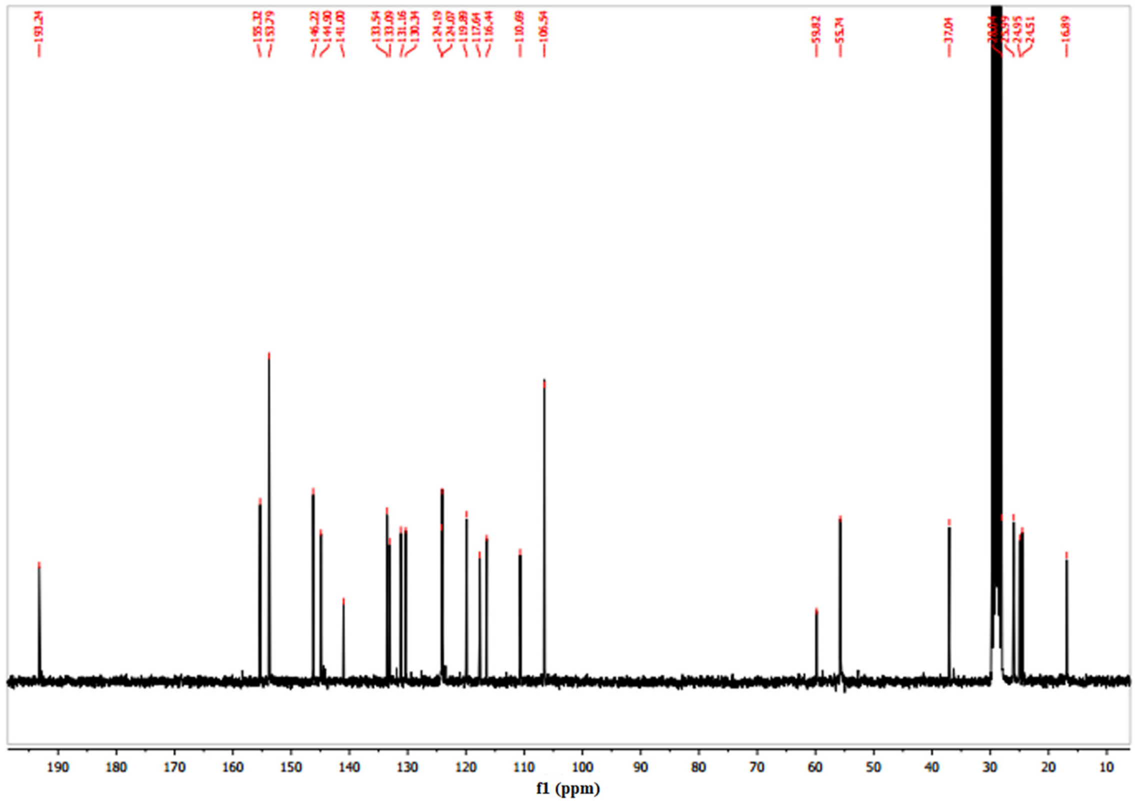

Figure S26. ^{13}C NMR spectrum of compound **4f**.

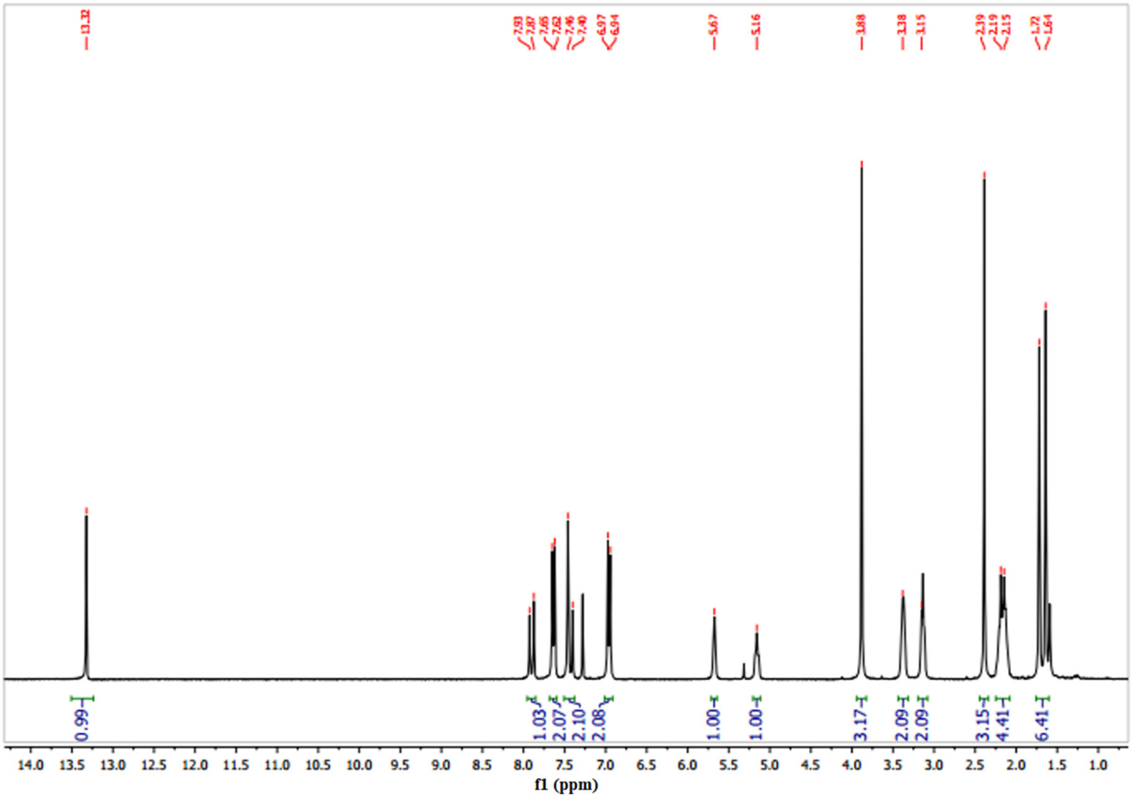

Figure S27. ^{1}H NMR spectrum of compound **5´b**.

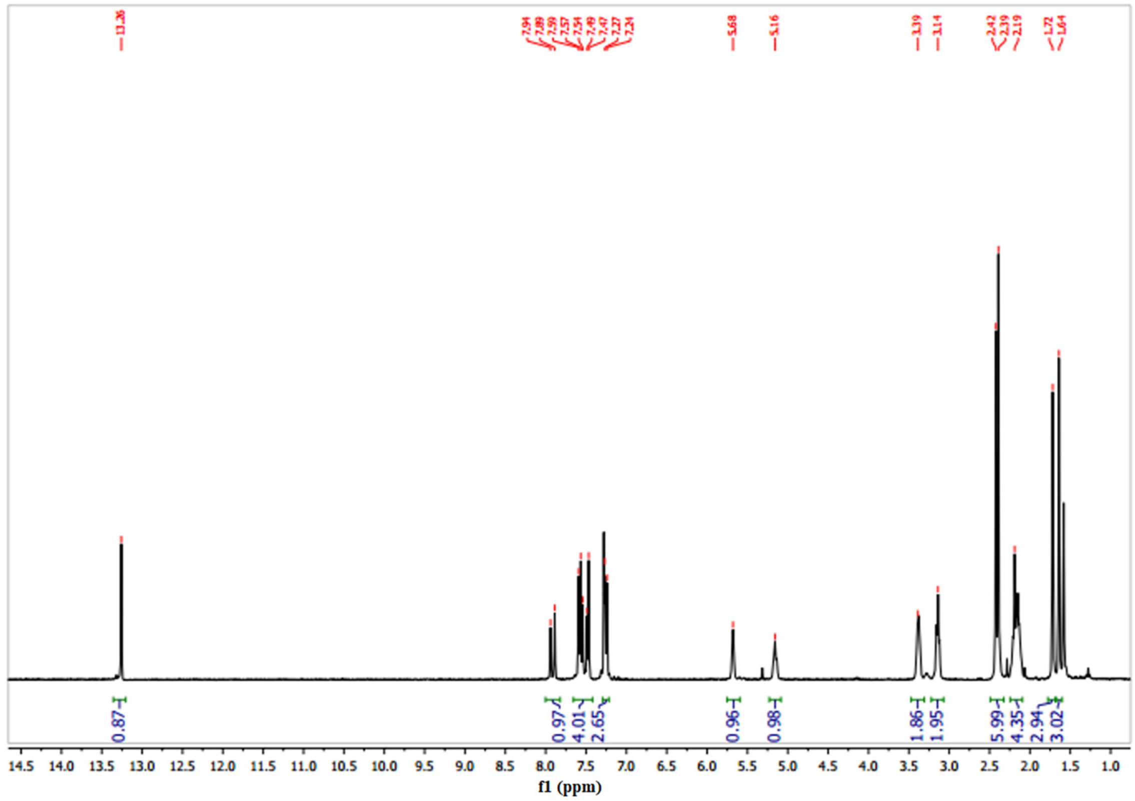

Figure S28. ^{1}H NMR spectrum of compound **5´c**.

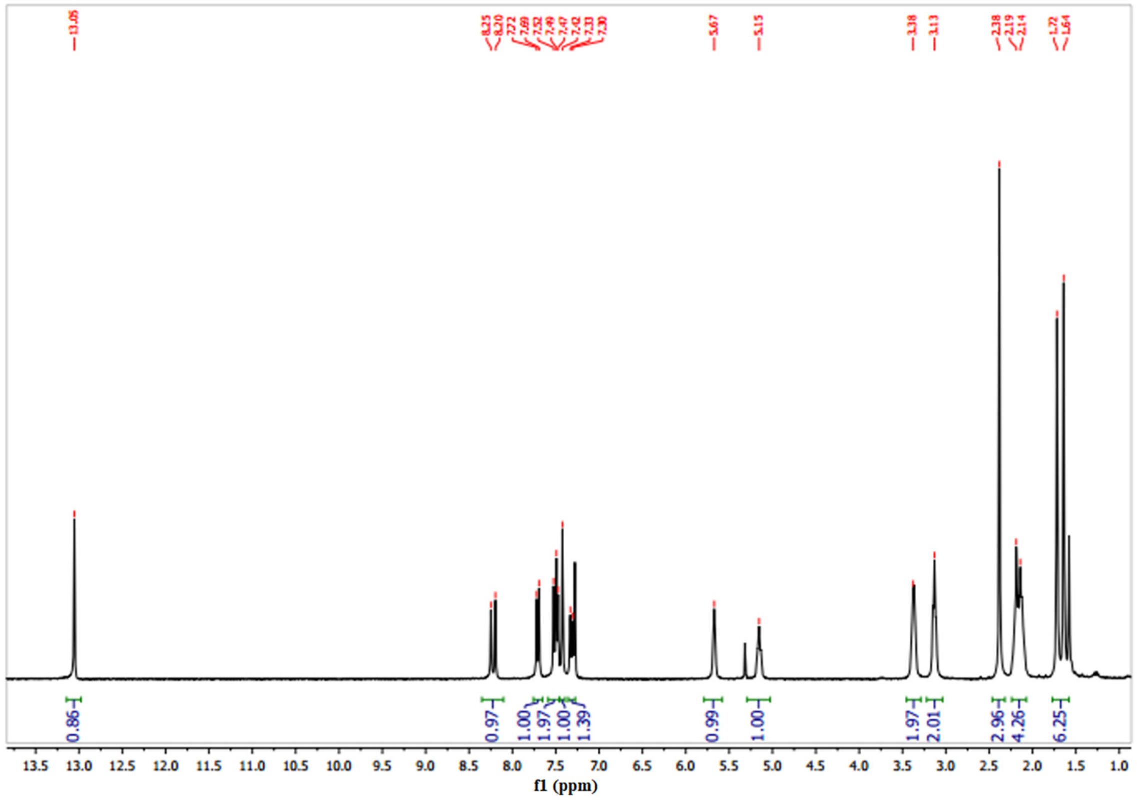

Figure S29. ^{1}H NMR spectrum of compound **5´d**.

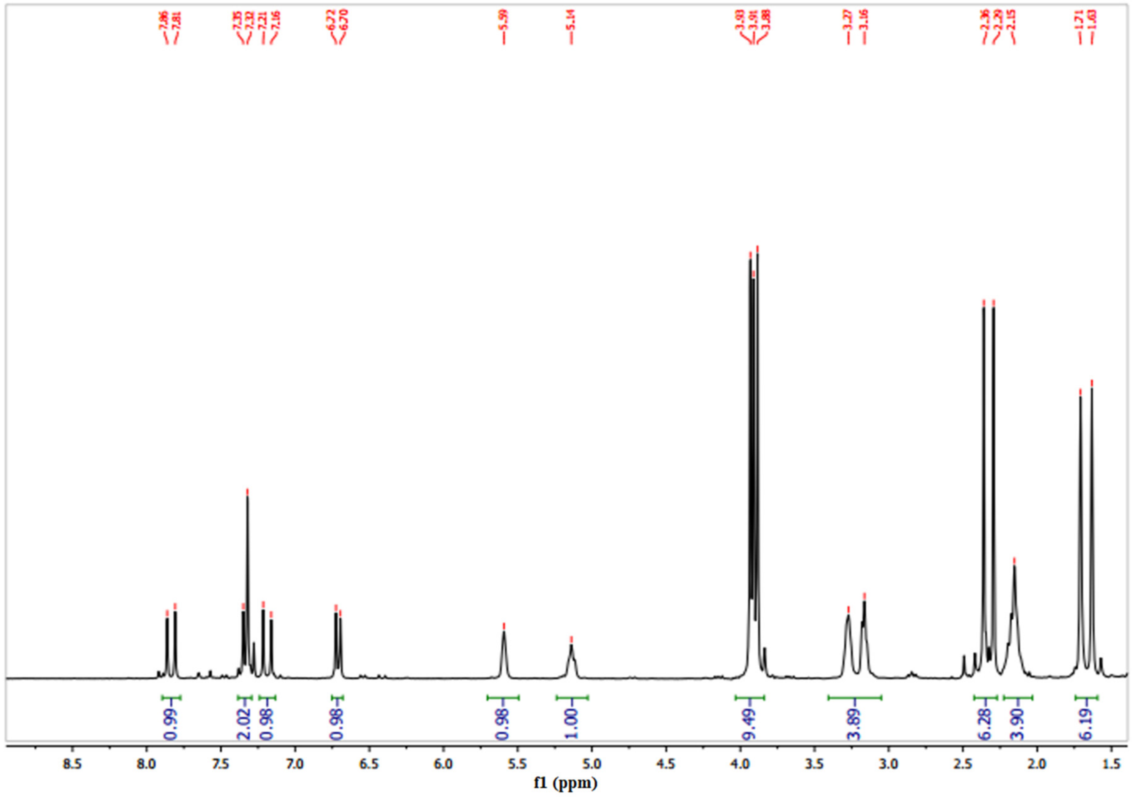

Figure S30. ^{1}H NMR spectrum of compound **5e**.

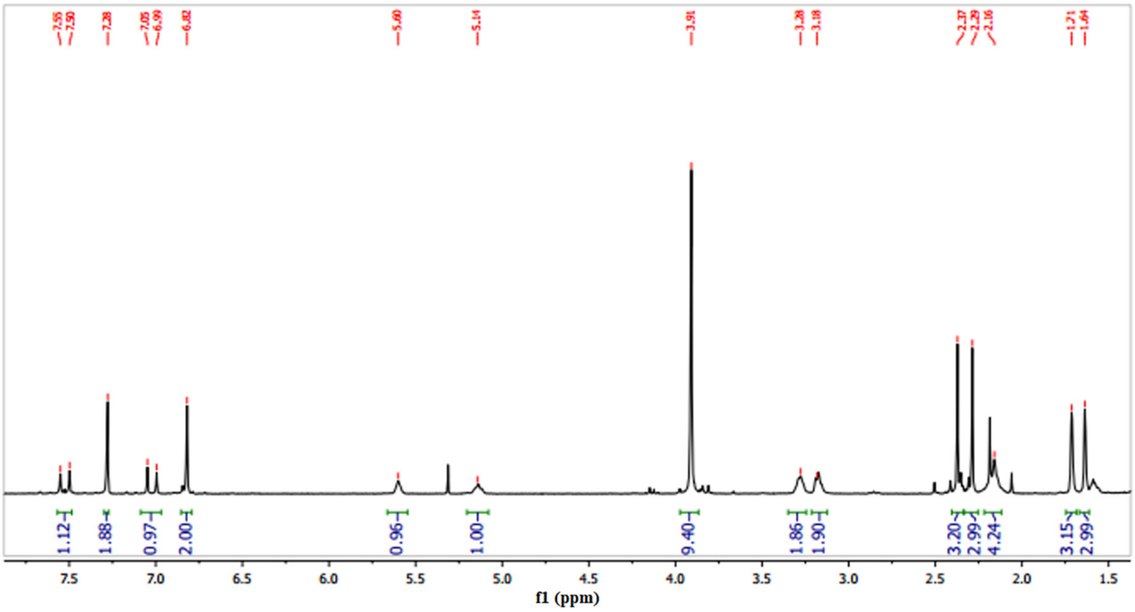

Figure S31. ^{1}H NMR spectrum of compound **5f**.

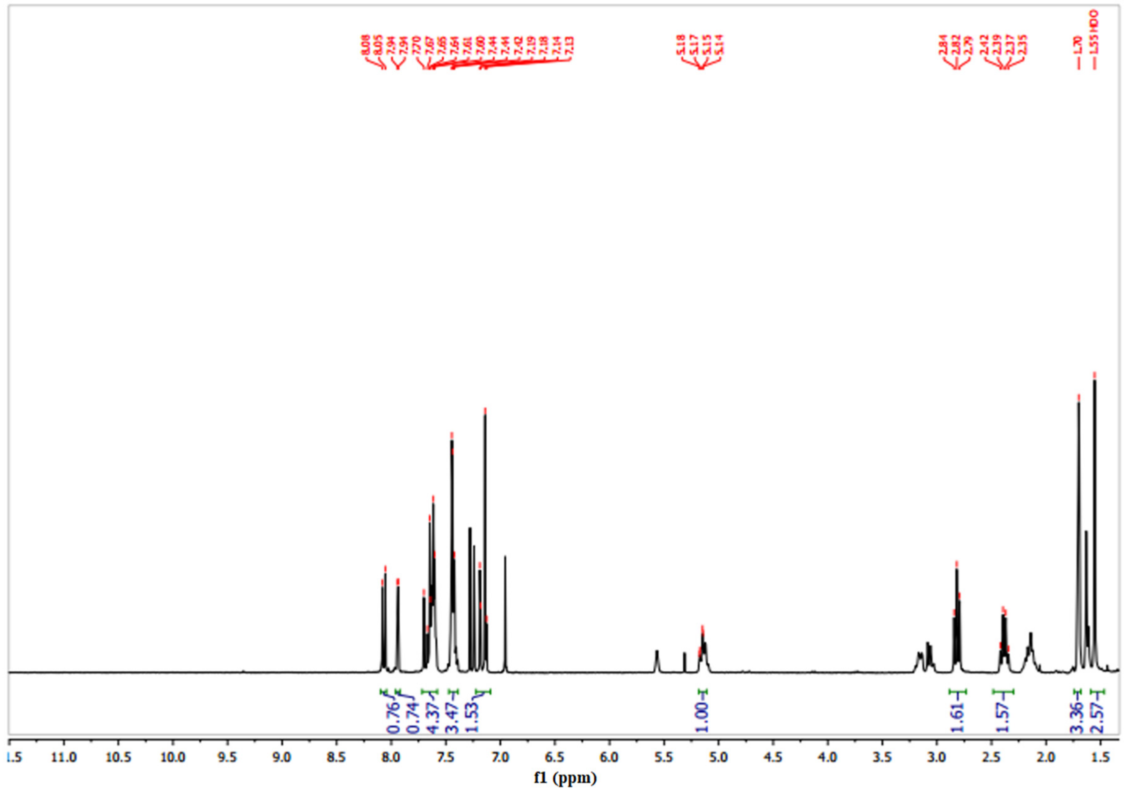

Figure S32. ^{1}H NMR spectrum of compound **6a**.

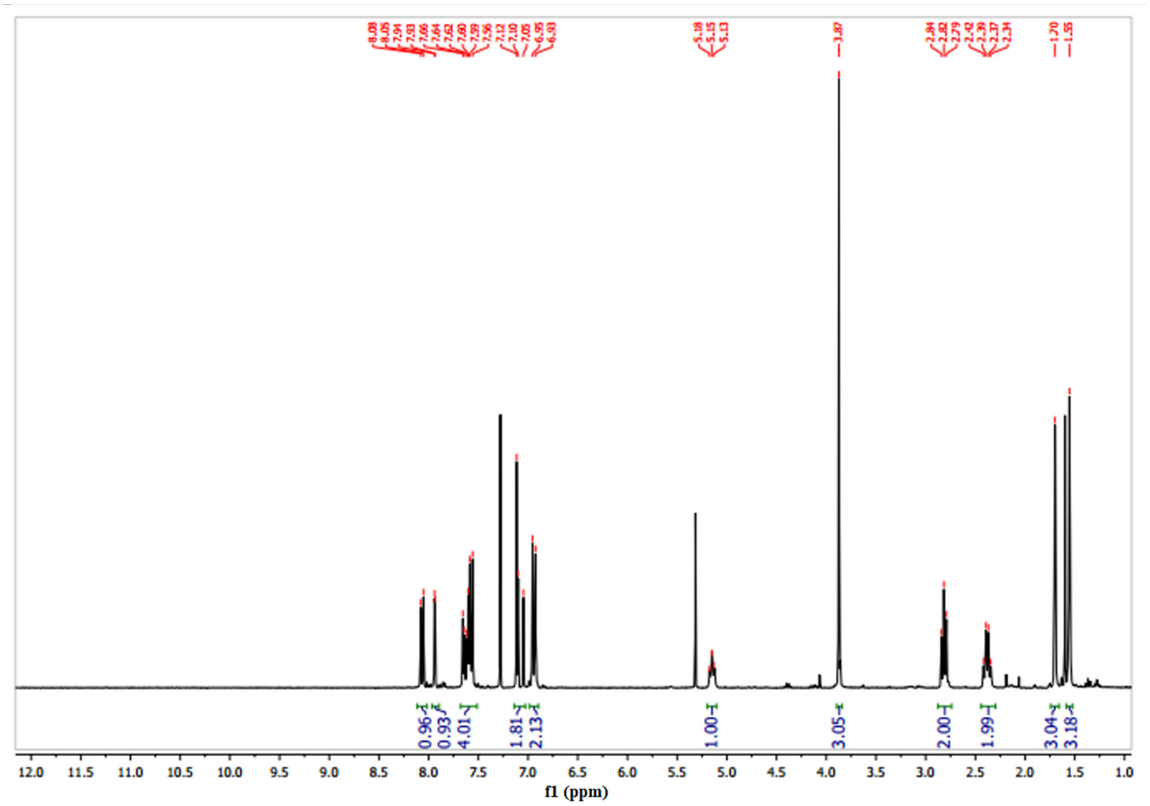

Figure S33. ^{1}H NMR spectrum of compound **6b**.

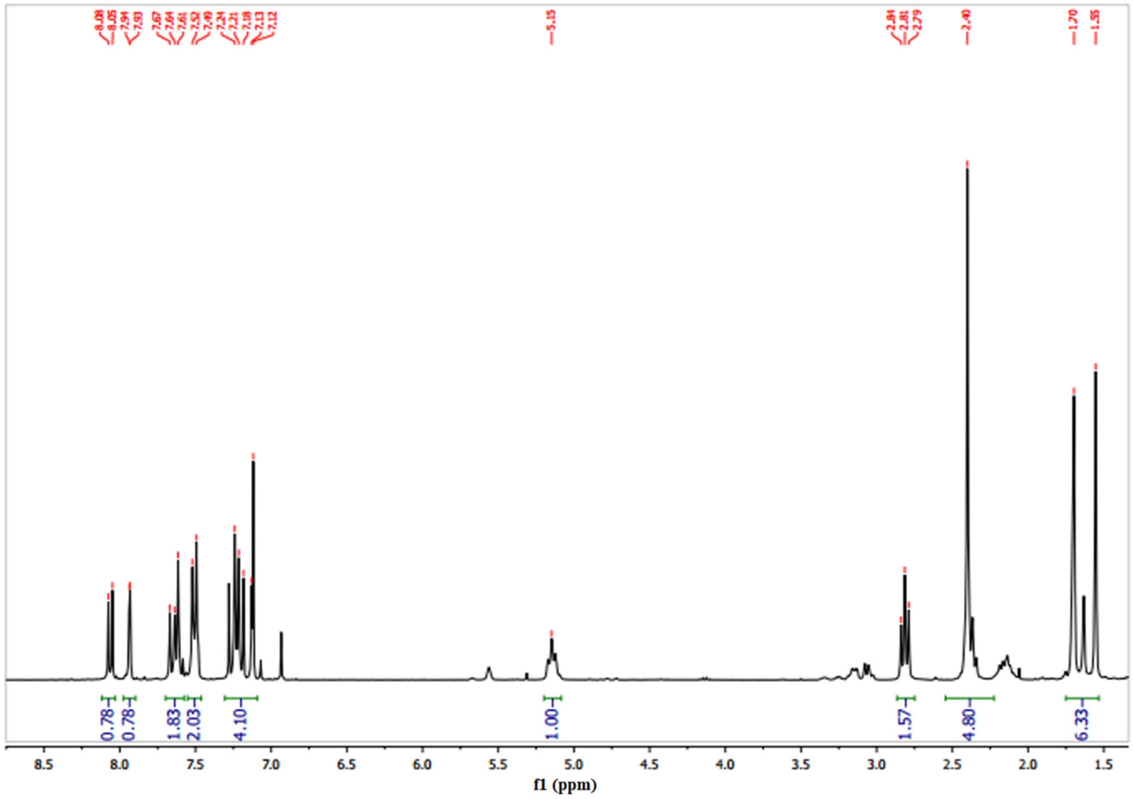

Figure S34. ^{1}H NMR spectrum of compound **6c**.

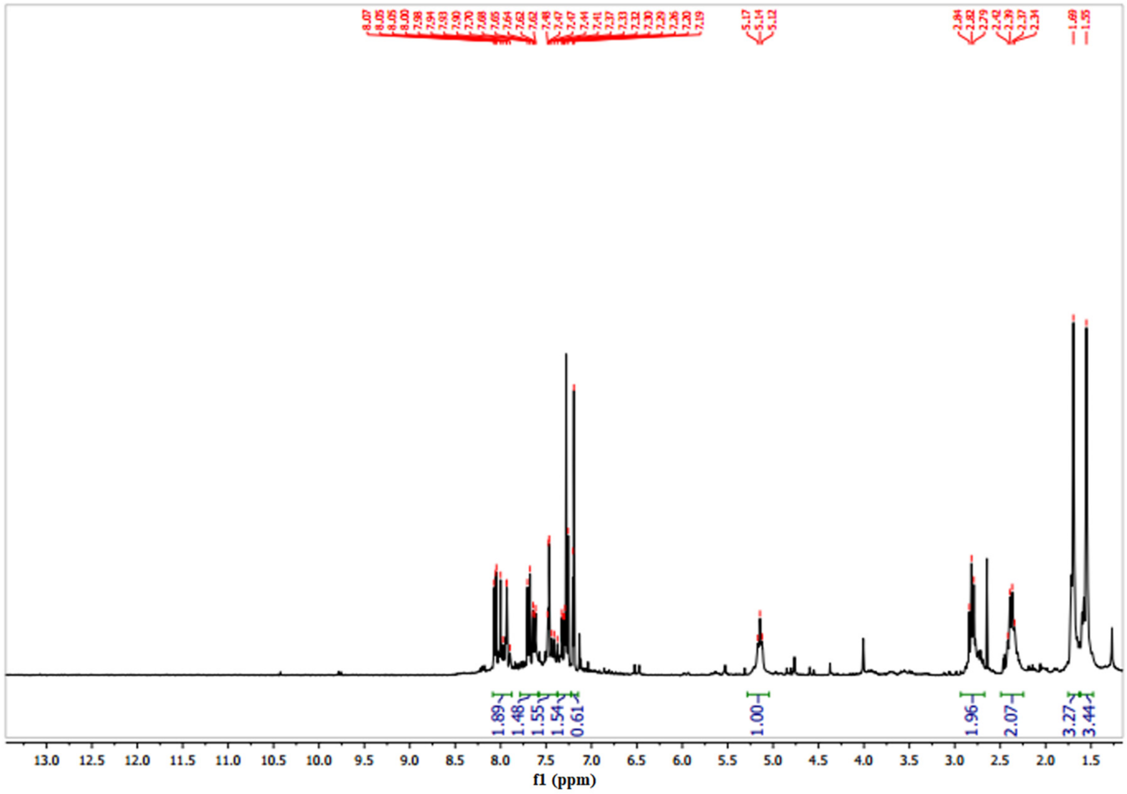

Figure S35. ^{1}H NMR spectrum of compound **6d**.

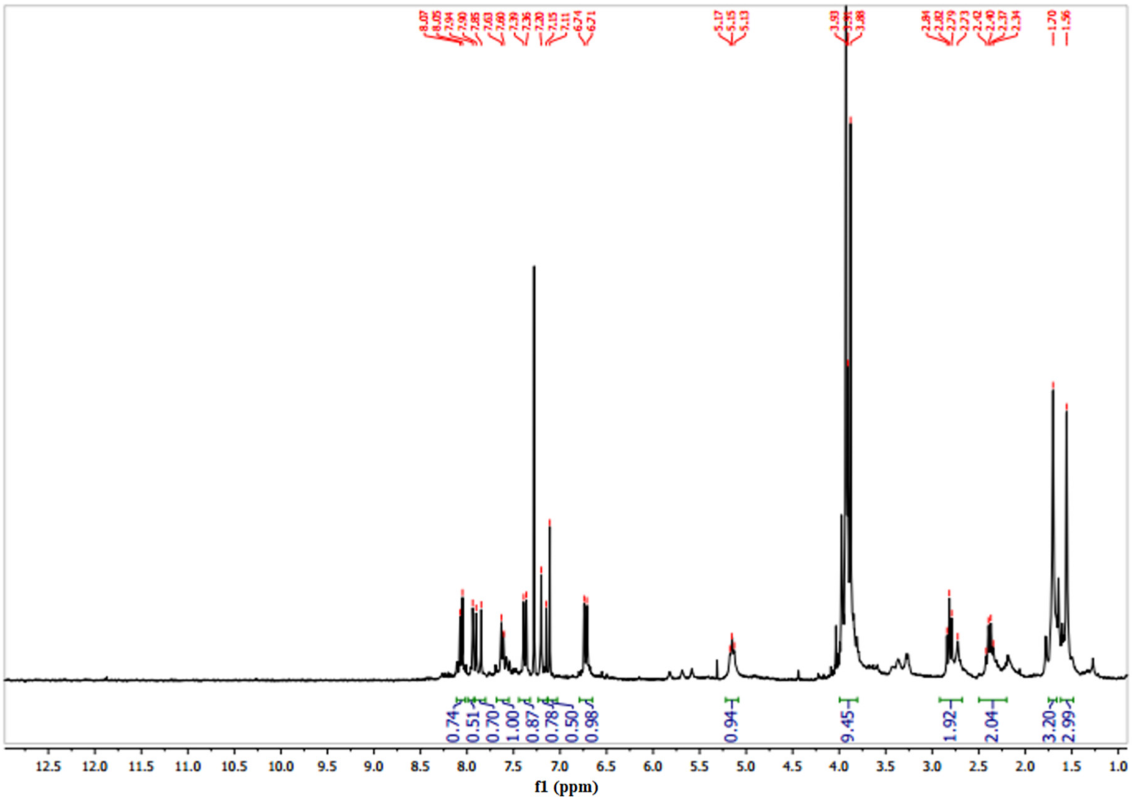

Figure S36. ^{1}H NMR spectrum of compound **6e**.

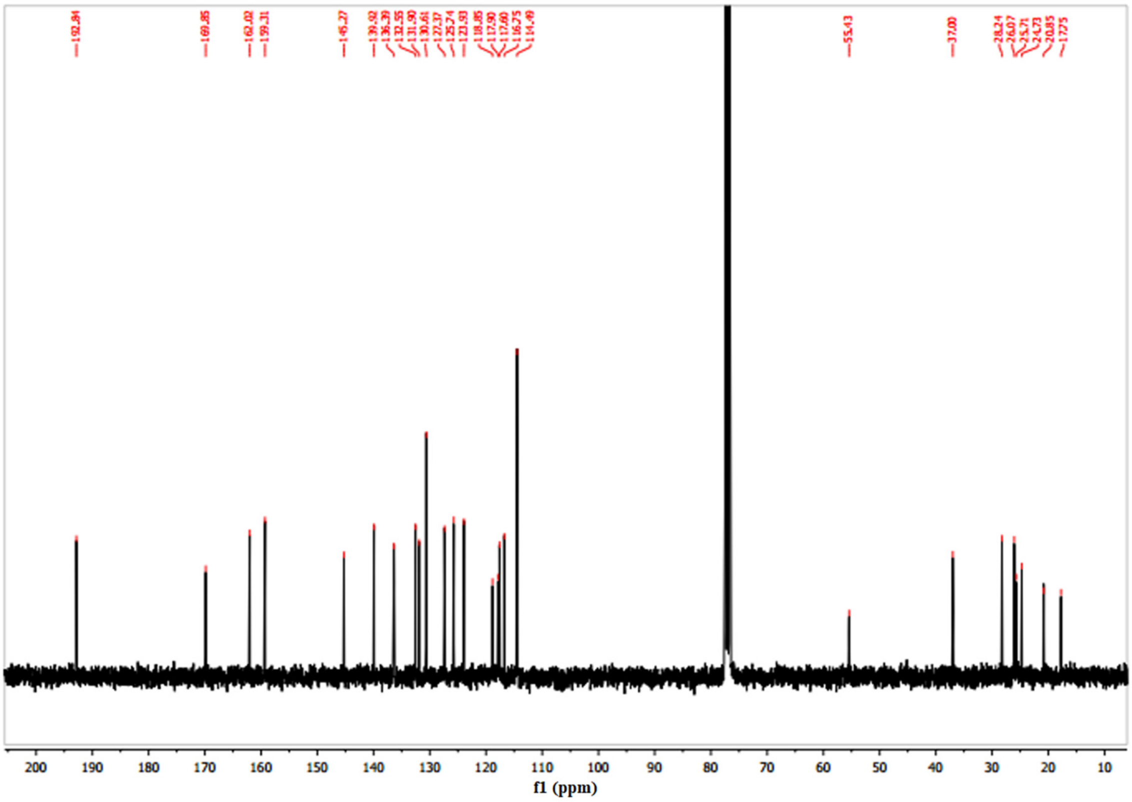

Figure S37. ^{13}C NMR spectrum of compound **5´b**.

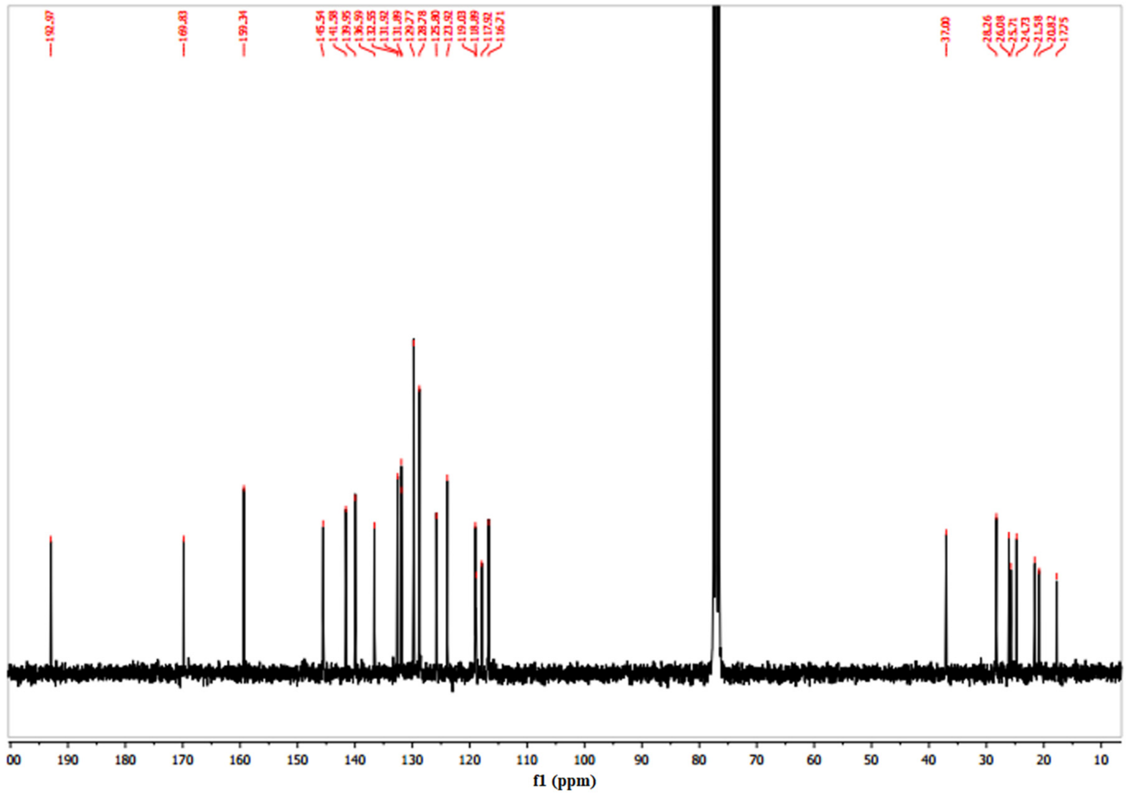

Figure S38. ^{13}C NMR spectrum of compound **5´c**.

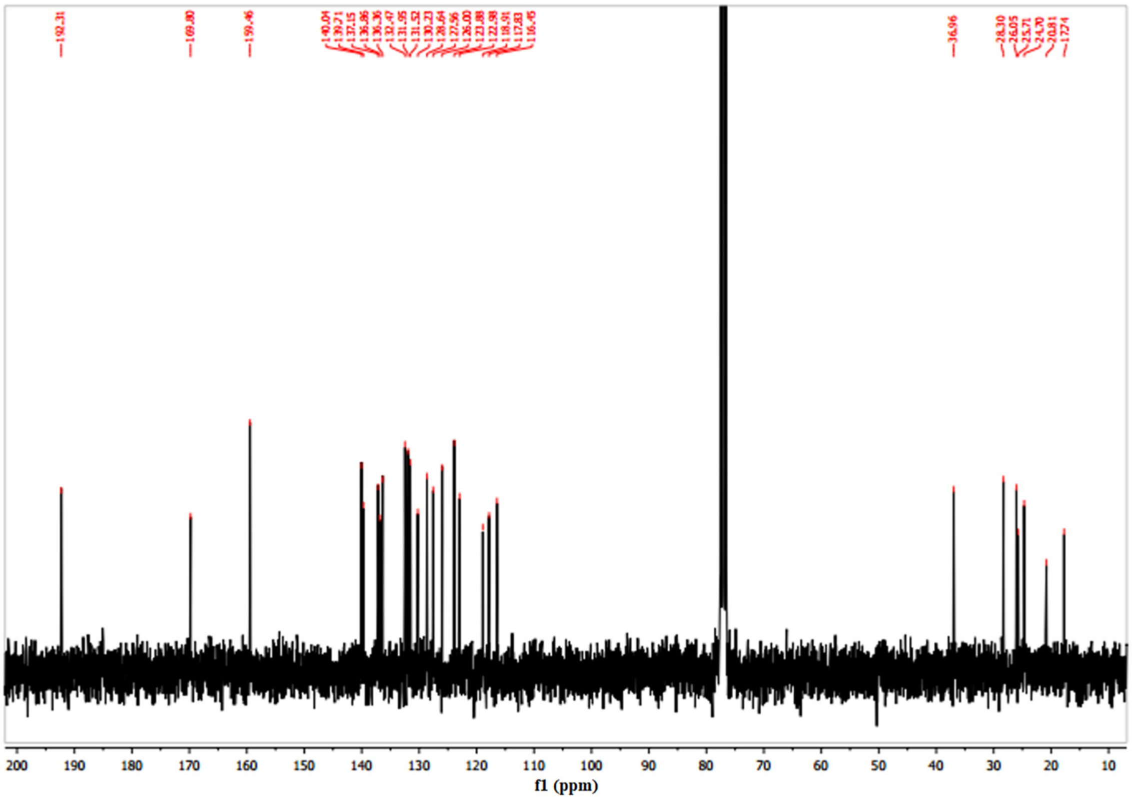

Figure S39. ^{13}C NMR spectrum of compound **5´d**.

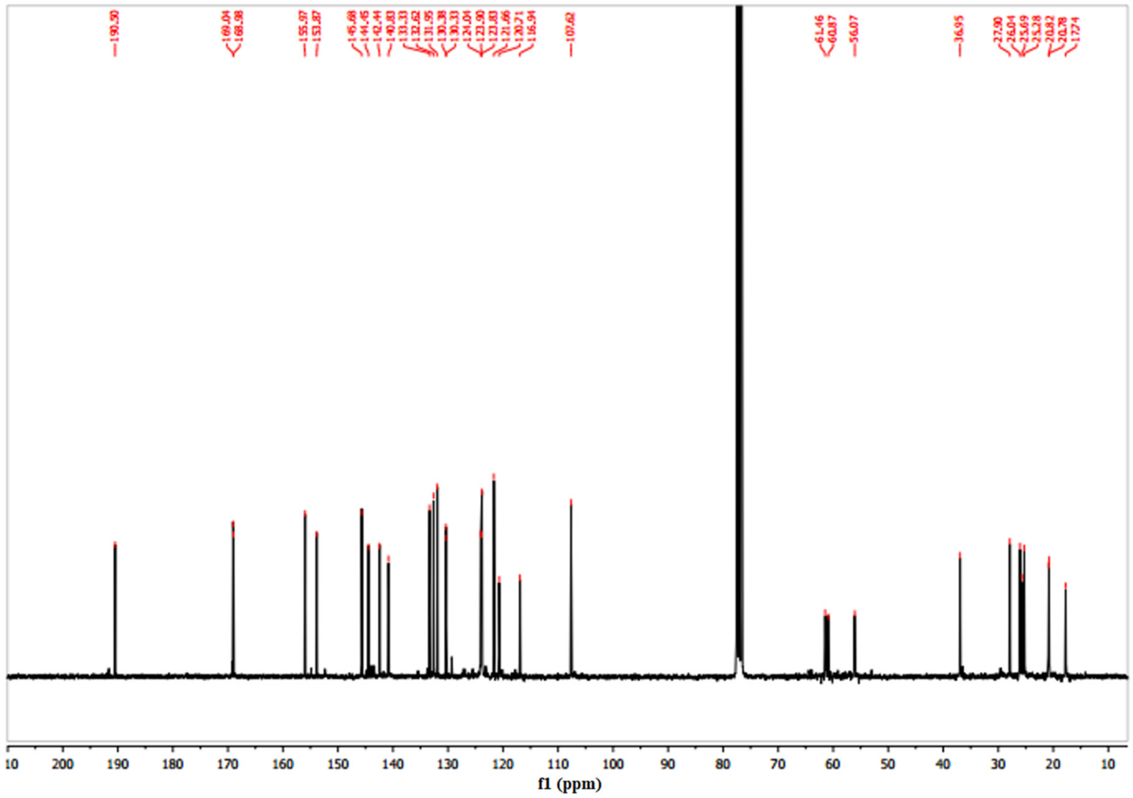

Figure S40. ^{13}C NMR spectrum of compound **5e**.

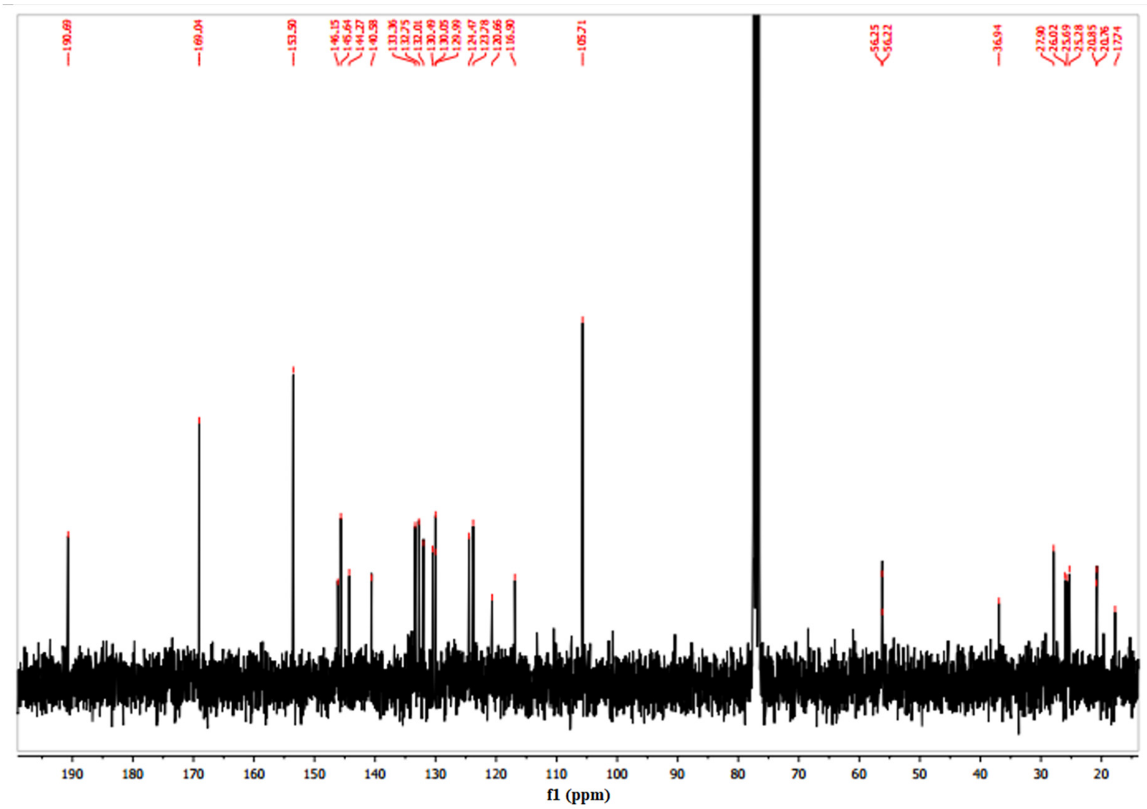

Figure S41. ^{13}C NMR spectrum of compound **5f**.

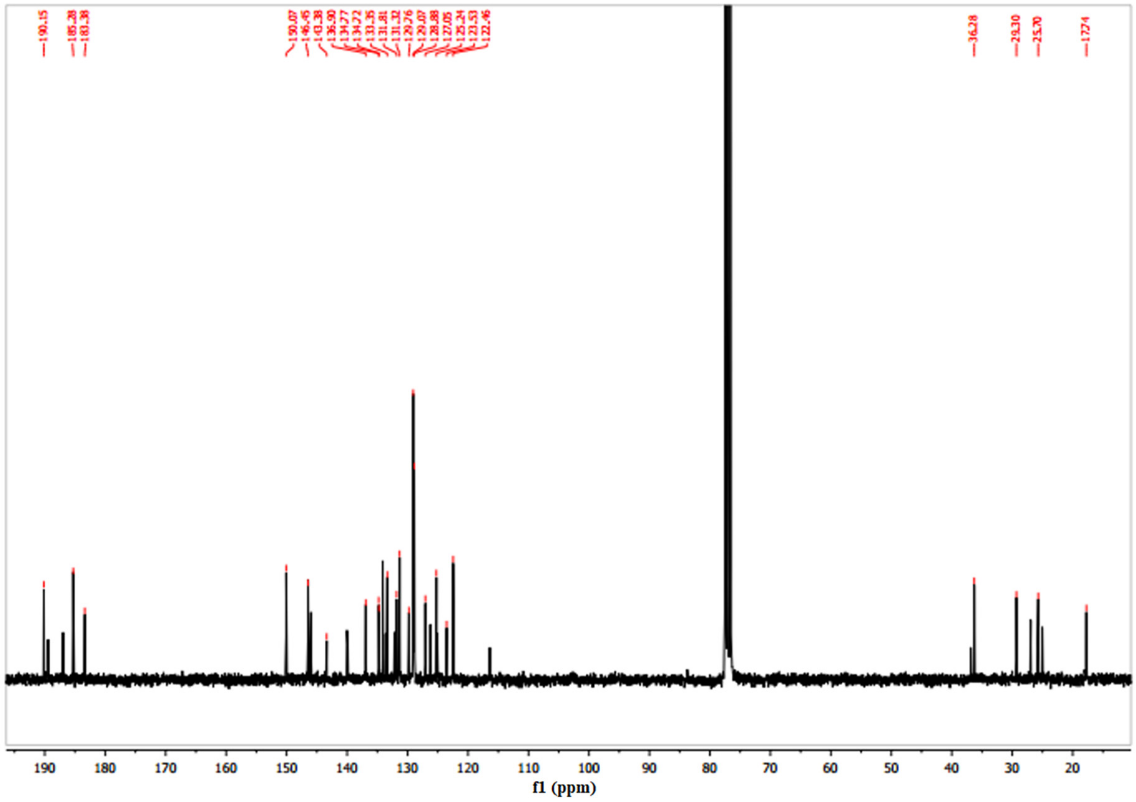

Figure S42. ^{13}C NMR spectrum of compound **6a**.

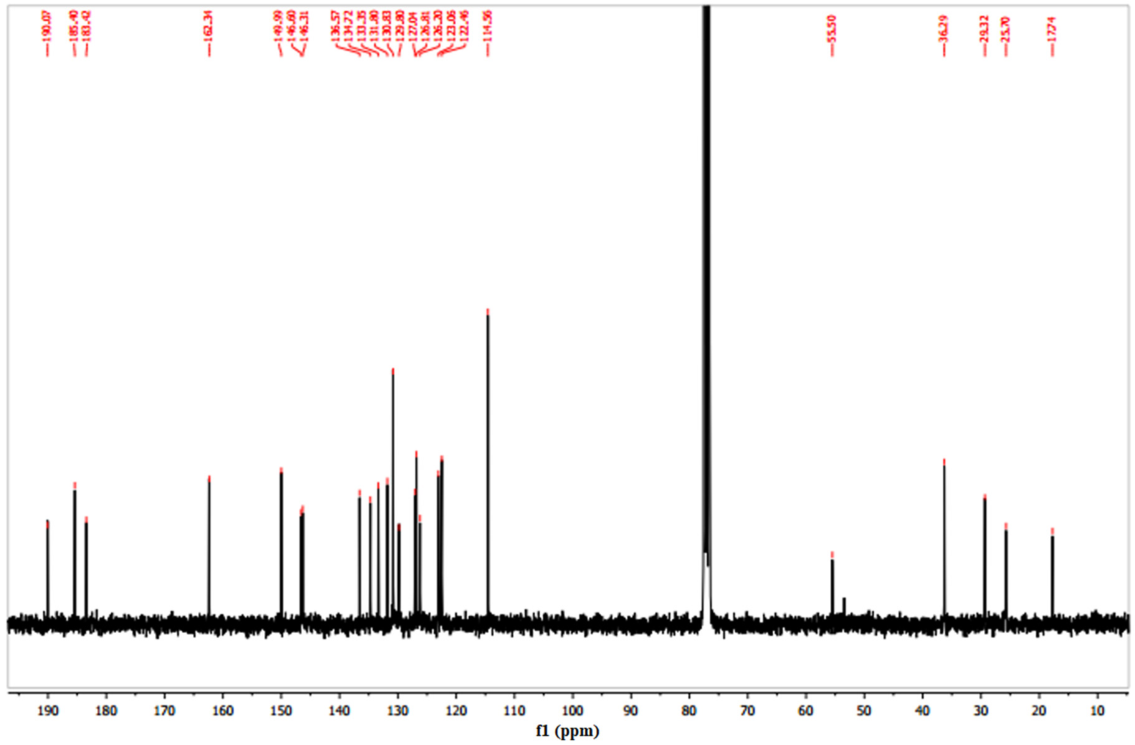

Figure S43. ^{13}C NMR spectrum of compound **6b**.

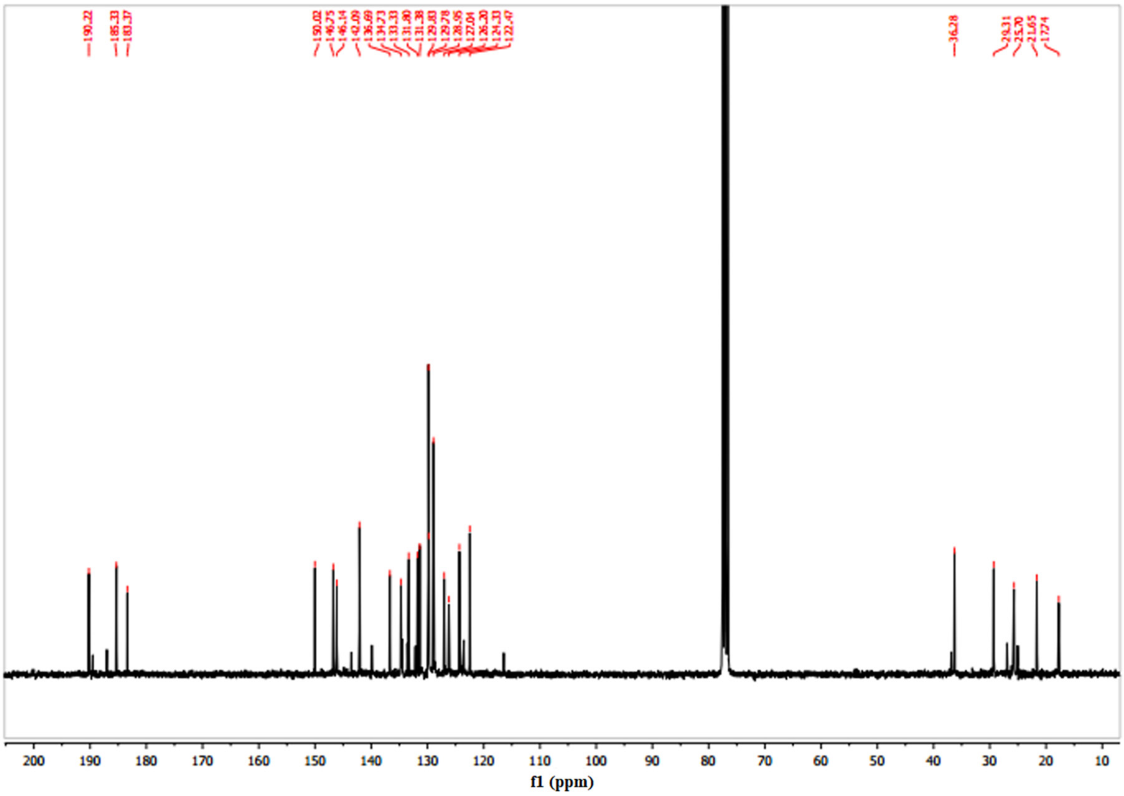

Figure S44. ^{13}C NMR spectrum of compound **6c**.

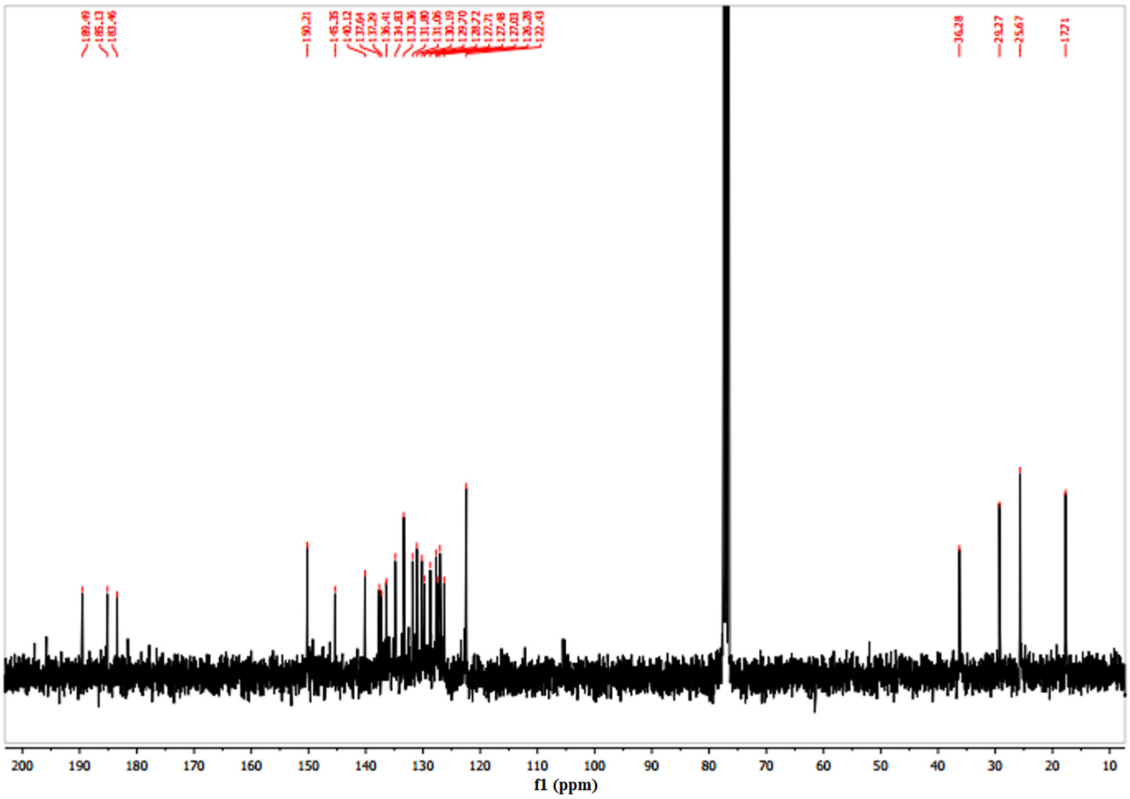

Figure S45. ^{13}C NMR spectrum of compound **6d**.

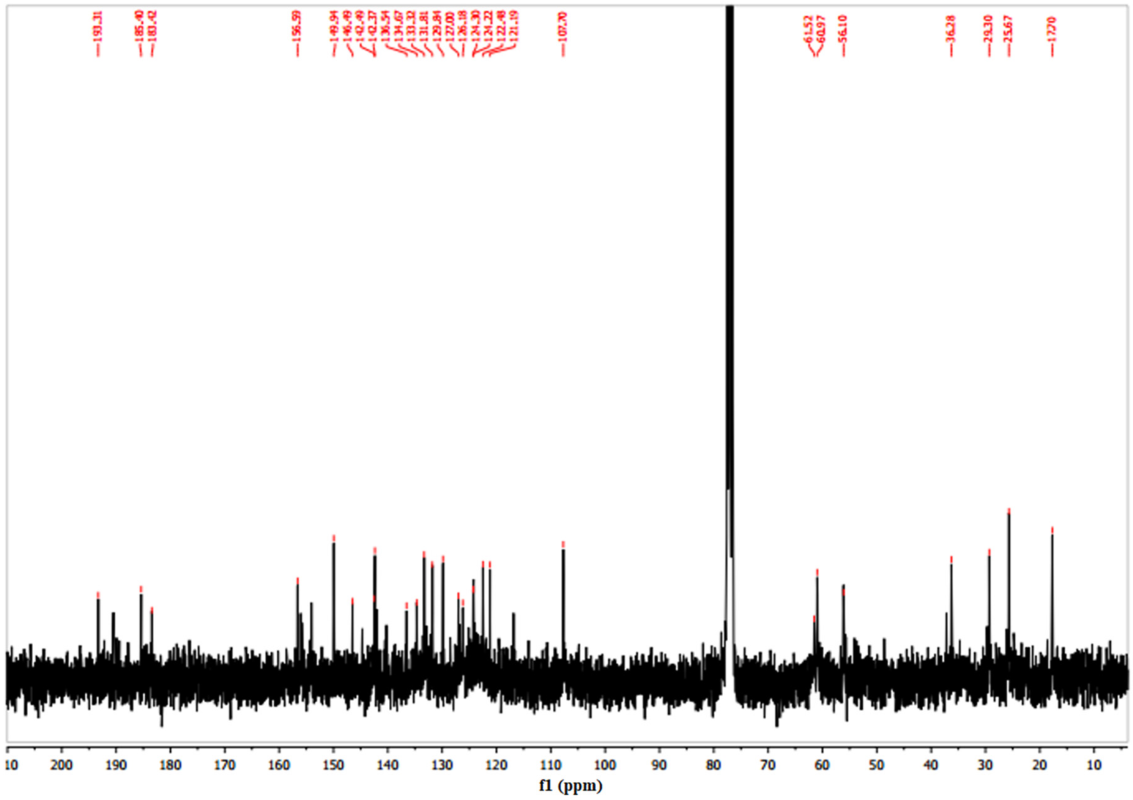

Figure S46. ^{13}C NMR spectrum of compound **6e.**

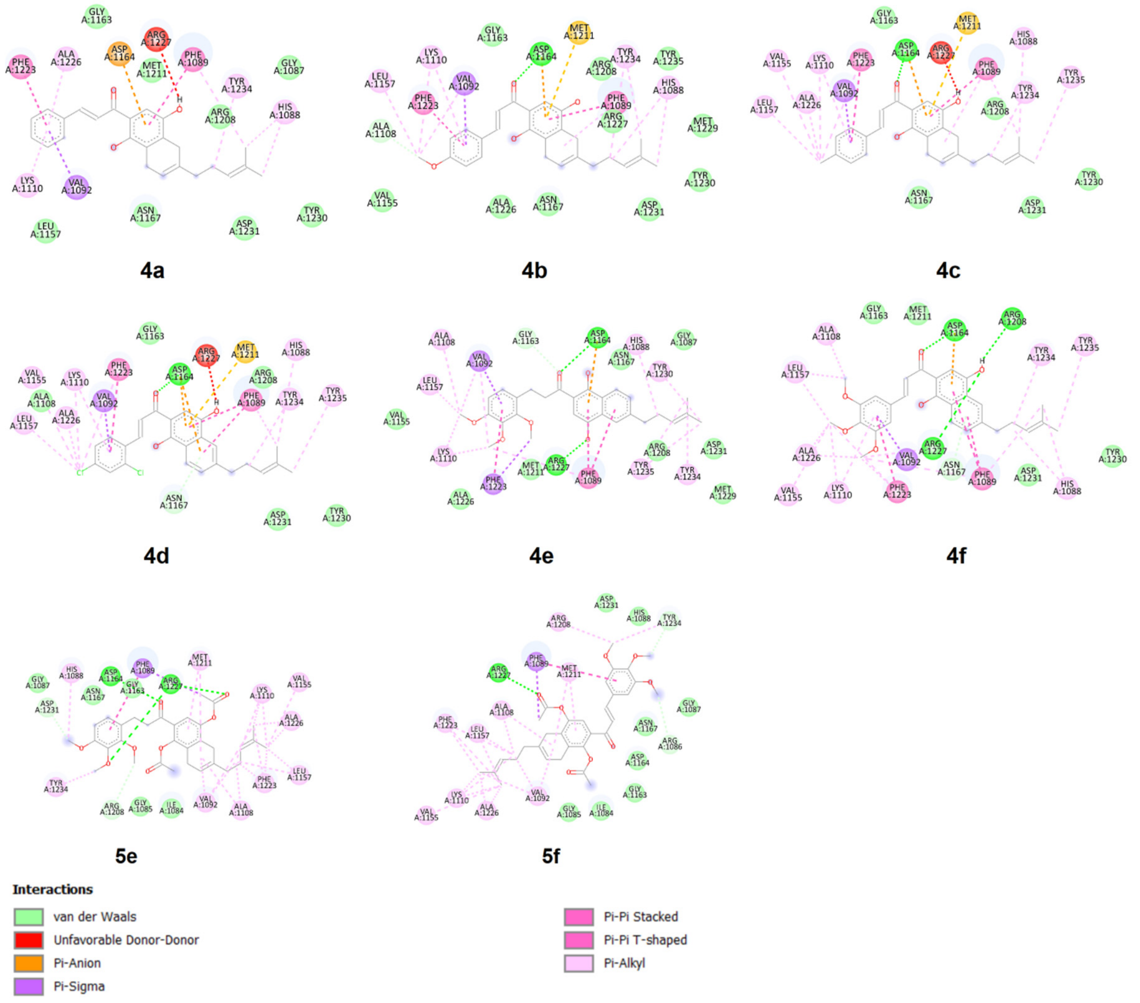

Figure S47. Plotted 2D maps of H-bonds and hydrophobic interactions of CNQ **5e** and **5f** with c-MET residues.

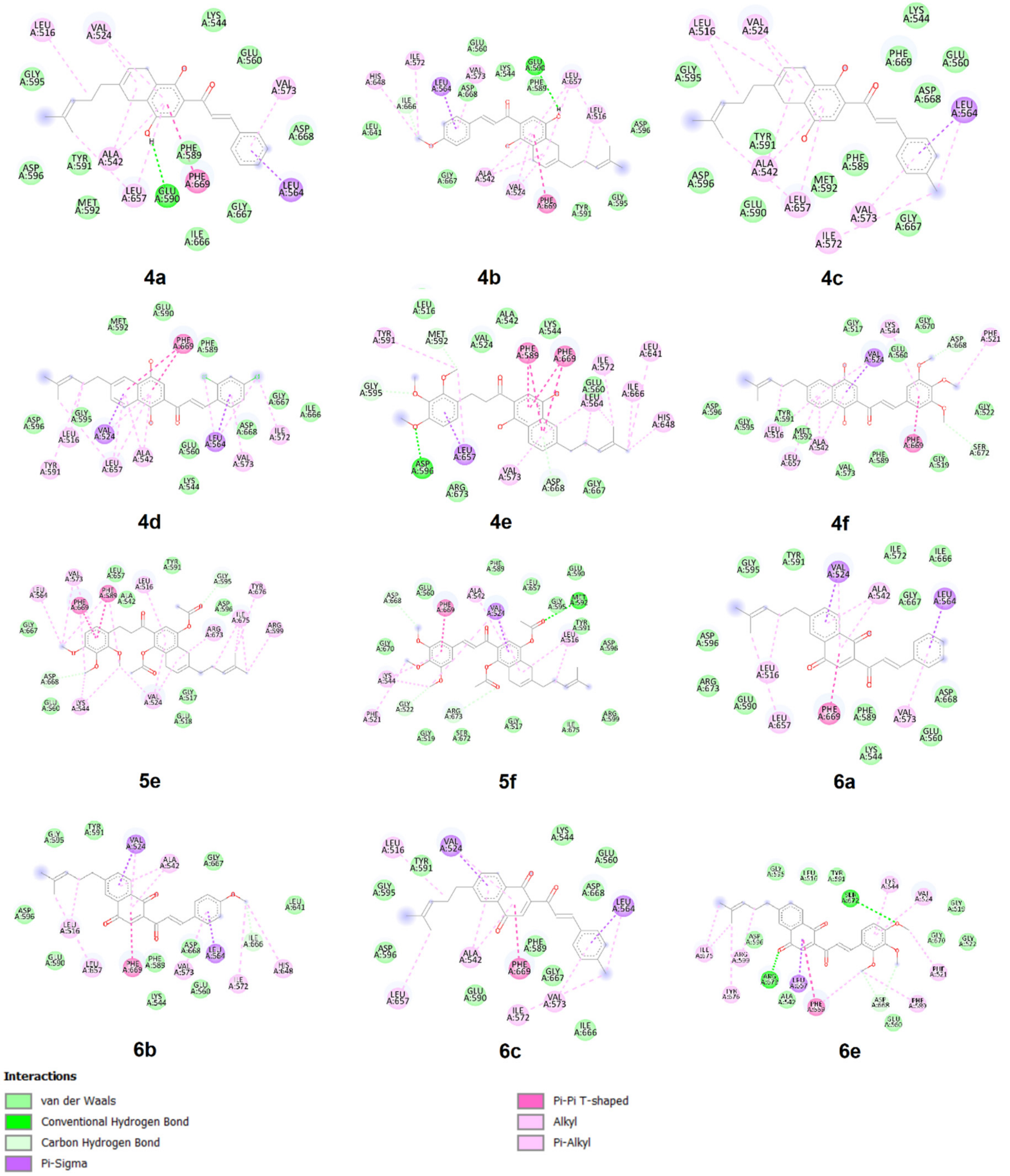

Figure S48. Plot 2 D-maps of H-bonds and hydrophobic interactions of **5e**, **5f**, and **6a–c**,**e** with TRKA residues.

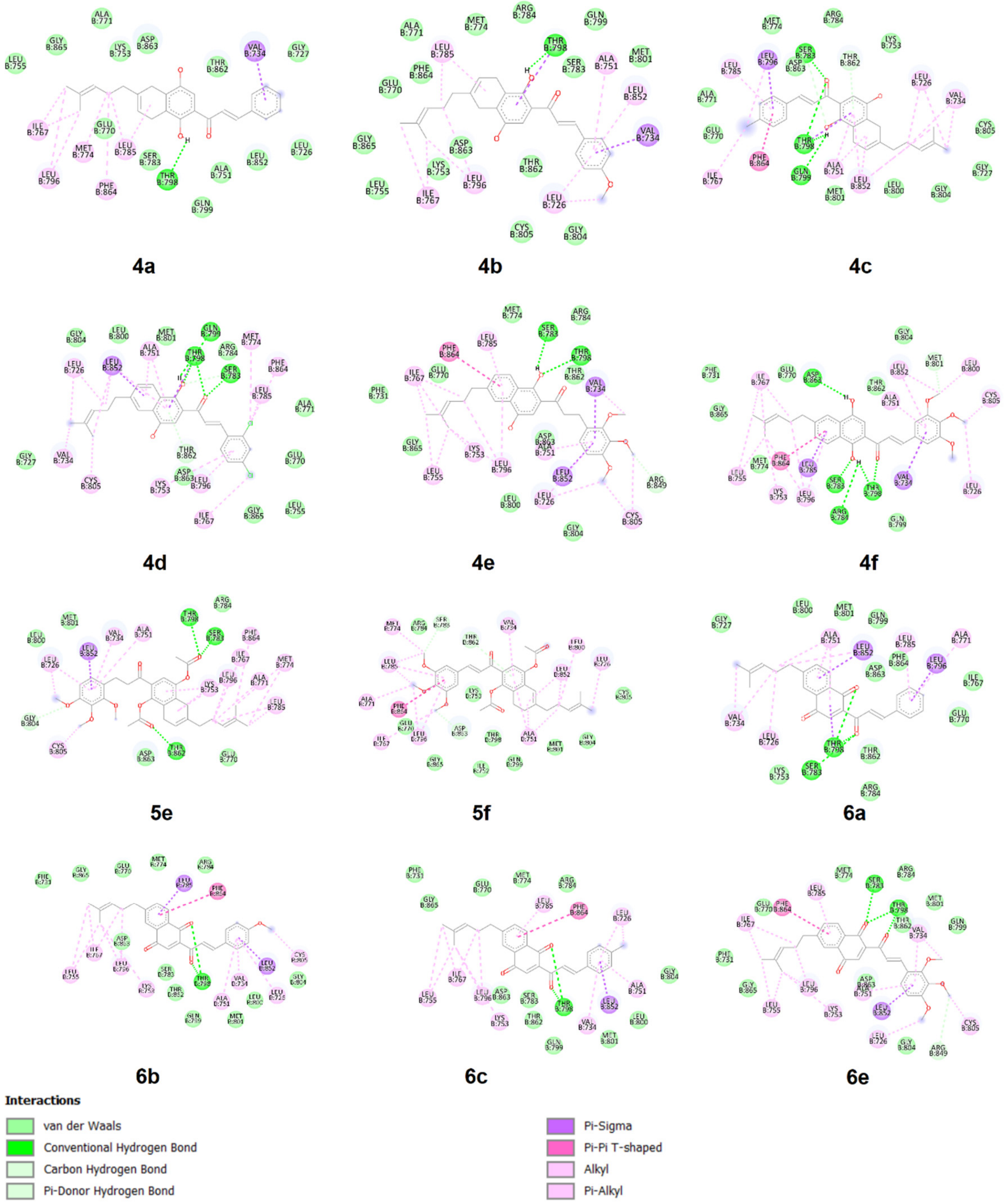

Figure S49. Plotted 2D maps of H-bonds and hydrophobic interactions of CNQ **5e**, **5f**, and **6a–c**,**e** with HER2 residues.

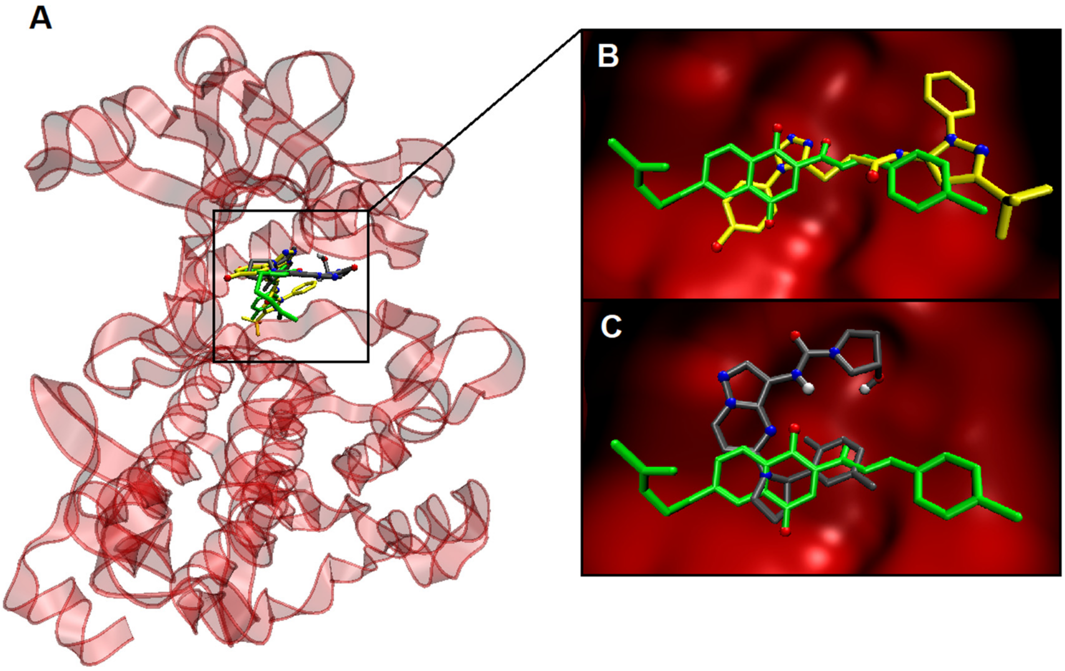

Figure S50. A: Overlapping of the docking poses for CNQ hybrid **6c** (green), ligand 2 (yellow), and larotrectinib (grey) into TRKA. Superimposition of the docking poses for B: **6c** and ligand 1, and C: **6c** and larotrectinib.

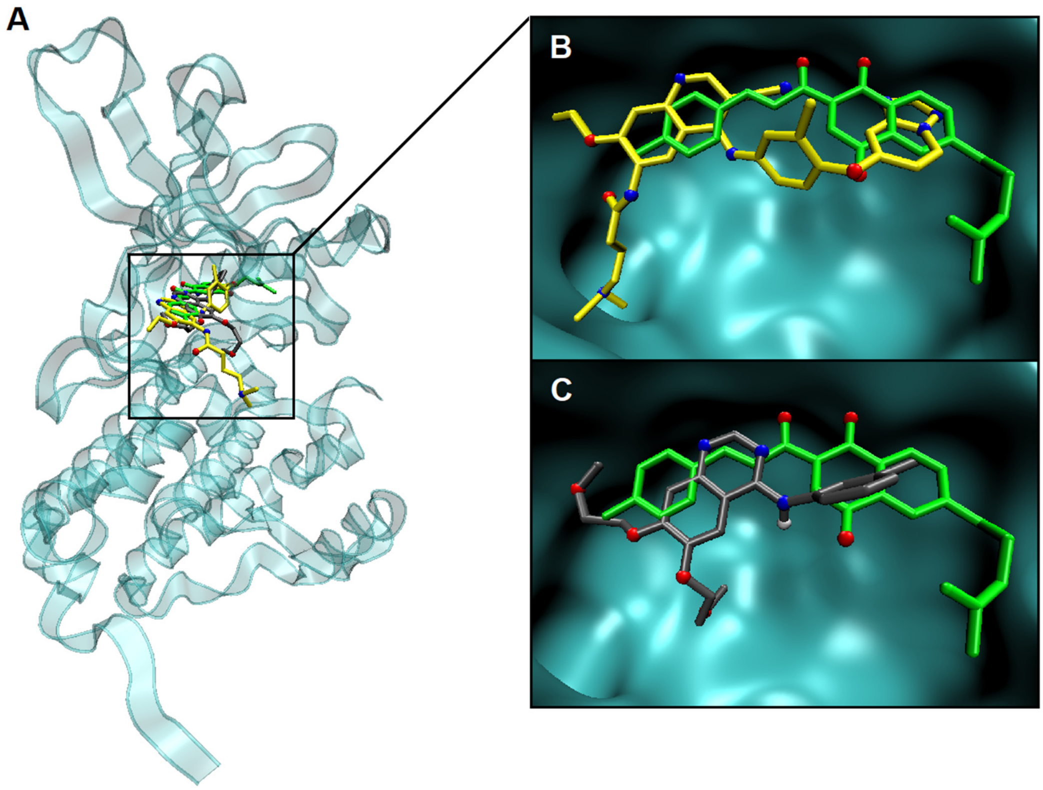

Figure S51. A: Overlapping of the docking poses for CNQ hybrid **6c** (green), ligand 2 (yellow), and erlotinib (grey) into HER2. Superimposition of the docking poses for B: **6c** and ligand 1, and C: **6c** and erlotinib.
