# Supplementary material for: 2-Acetyl-5,8-dihydro-6-(4-methyl-3-pentenyl)-1,4-naphthohydroquinone-Derived Chalcones as Potential Anticancer Agents
Source: Molecules. 2023 Oct 19;28(20):7172. doi: 10.3390/molecules28207172 (PMC10609043; doi:10.3390/molecules28207172)
Supplement: Supplementary file 1 [file molecules-28-07172-s001.zip › Table S1.pdf]

Table S1. Predicted binding free energy values (ΔG_{bin} kcal/mol) of synthesized cytotoxic hybrids with selected proteins overexpressed in cancer.

**Compounds**	**Target proteins**															**Total avge.**
	**DHFR**	**FGFR-2**	**VEGRF-2**	**NR3A1**	**EGFR**	**HER2**	**NR3A2**	**c-MET**	**TRKA**	**ERK2**	**MEK1**	**CK4**	**TPK**	**TopoII**	**TUB**	
**5e**	-7.5	-8.9	-7.7	-8.7	-7.2	-8.3	-8.6	-9.8	-8.9	-9.0	-9.2	-7.6	-8.6	-6.7	-8.8	-8.34
**5f**	-7.6	-8.9	-6.2	-9.3	-7.4	-8.6	-6.9	-9.7	-9.1	-9.1	-8.7	-8.8	-8.2	-6.4	-9.0	-8.26
**6a**	-10.0	-9.6	-10.3	-9.2	-10.6	-10.8	-8.8	-10.3	-10.5	-9.4	-10.5	-9.8	-10.3	-6.6	-9.7	-9.78
**6b**	-9.7	-9.2	-10.0	-9.0	-10.6	-10.2	-9.5	-10.3	-10.7	-9.5	-9.8	-9.8	-10.3	-6.5	-9.4	-9.63
**6c**	-9.8	-9.6	-10.5	-9.2	-11.1	-10.4	-9.8	-10.6	-11.0	-9.8	-10.2	-8.8	-10.4	-7.0	-9.7	-9.86
**6e**	-9.0	-8.8	-10.0	-9.3	-10.0	-10.2	-8.5	-9.8	-9.6	-7.6	-9.9	-9.2	-9.9	-6.5	-8.8	-9.14
**P avge.**	**-8.88**	**-9.17**	**-9.12**	**-9.12**	**-9.53**	**-9.75**	**-8.68**	**-10.08**	**-9.97**	**-9.07**	**-9.72**	**-9.00**	**-9.62**	**-6.62**	**-9.22**	
**Doxorubicin**	-7.7	-9.0	-9.1	-7.7	-8.9	-6.2	-8.2	-8.7	-9.0	-9.2	-9.1	-7.2	-10.1	-7.7	-8.1	

Proteins with their respective (PDB) entries: **DHFR**: Dihydrofolate reductase (1DLS); **FGFR-2**: Fibroblast growth factor receptor 2 (1GJO); **VEGRF-2**: Vascular endothelial growth factor receptor 2 (3VHE); **NR3A1**: Estrogen receptor α (3ERT); **EGFR**: Epidermal growth factor receptor (5GTY); **HER2**: Epidermal growth factor receptor 2 (7JXH); **TRKA**: Tropomyosin receptor kinase A (6PL2); **NR3A2**: Estrogen receptor beta (2QTU); **c-MET**: Mesenchymal-epithelial transition factor (3RHK); **TRKA**: Tropomyosin receptor kinase A (6PL2); **ERK2**: Extra-cellular signal-regulated kinase 2 (2OJG); **MEK1**: MAPK/ERK kinase (4AN3); **CK4**: Cyclin-dependent kinase 4 (1G3N); **TPK**: Tyrosine-protein kinase (4EHZ); TopoII: Topoisomerase II (5GWK); **TUB**: alpha tubulin (6WSL) P avge.: Protein average. mean of the ΔG_{bin} values for the interactions of each protein with all the hybrids; the two proteins with the highest global chalcones-1,4-Naphthoquinone /Hydroquinones affinity are highlighted on red colour.
