# Supplementary material for: 2-Acetyl-5,8-dihydro-6-(4-methyl-3-pentenyl)-1,4-naphthohydroquinone-Derived Chalcones as Potential Anticancer Agents
Source: Molecules. 2023 Oct 19;28(20):7172. doi: 10.3390/molecules28207172 (PMC10609043; doi:10.3390/molecules28207172)
Supplement: Supplementary file 1 [file molecules-28-07172-s001.zip › Table S2.pdf]

Table S2. Comparison (ΔG_{bin}, kcal/mol) of chalcones-1,4-Naphthoquinone/Hydroquinone hybrids with kinase proteins overexpressed in cancer.

Compounds	Target proteins						cLogP
	EGFR	HER2	c-MET	TRKA	MEK1	TPK	
**4a**	-10.9	-10.5	-9.7	-10.1	-10.3	-10.2	6.11
**4b**	-10.6	-9.8	-9.9	-10.3	-9.8	-10.0	6.04
**4c**	-11.1	-9.9	-10.4	-10.4	-9.8	-10.6	6.45
**4d**	-11.2	-10.0	-10.3	-10.4	-10.2	-10.7	7.32
**4e**	-10.5	-9.5	-9.3	-9.7	-9.0	-9.0	5.90
**4f**	-10.0	-9.4	-9.4	-9.1	-10	-8.4	5.90
**5a**	-8.8	-9.4	-9.6	-9.7	-9.5	-9.3	6.77
**5´b**	-9.2	-9.7	-9.4	-10.1	-9.3	-8.8	6.37
**5´c**	-9.2	-10.1	-10.2	-10.4	-10.2	-9.7	6.78
**5´d**	-9.3	-10	-9.9	-10.3	-9.9	-9.6	7.65
**5e**	-7.2	-8.3	-9.8	-8.9	-9.2	-8.6	6.56
**5f**	-7.4	-8.6	-9.7	-9.1	-8.7	-8.2	6.56
**6a**	-10.6	-10.8	-10.3	-10.5	-10.5	-10.3	5.53
**6b**	-10.6	-10.2	-10.3	-10.7	-9.8	-10.3	5.46
**6c**	-11.1	-10.4	-10.6	-11.0	-10.2	-10.4	5.87
**6d**	-11.2	-10.6	-10.4	-11.0	-10.4	-10.6	6.74
**6e**	-10.0	-10.2	-9.8	-9.6	-9.9	-9.9	5.32
**6f**	-8.6	-9.5	-10.4	-9.1	-10.2	-9.5	5.32

Proteins with their respective (PDB) entries: **EGFR**: Epidermal growth factor receptor (5GTY); **HER2**: Epidermal growth factor receptor 2 (7JXH); **c-MET**: Mesenchymal-epithelial transition factor (3RHK); **TRKA**: Tropomyosin receptor kinase A (6PL2); **MEK1**: MAPK/ERK kinase (4AN3); **TPK**: Tyrosine-protein kinase (4EHZ).
