# Supplementary material for: 2-Acetyl-5,8-dihydro-6-(4-methyl-3-pentenyl)-1,4-naphthohydroquinone-Derived Chalcones as Potential Anticancer Agents
Source: Molecules. 2023 Oct 19;28(20):7172. doi: 10.3390/molecules28207172 (PMC10609043; doi:10.3390/molecules28207172)
Supplement: Supplementary file 1 [file molecules-28-07172-s001.zip › Table S3.pdf]

Table S3. Predicted binding free energy values (ΔGbin, kcal/mol) and binding site contacts of synthesized cytotoxic hybrids with amino acids of MEK1, TPK, and EGFR.

Compounds	ΔGbin	H-Bonds and Hydrophobic Contacts in the Binding Site*
		**MEK1** (mean ΔGbin = -9.72 kcal/mol)
**5e**	-9.2	Gly79, **Lys97**, Ile99, Leu115, Leu118, Ile141, **Arg189**, Asp190, Cys207, Asp208, Gly210, Val211, Leu215, Ile216, Met219*, Ala220, Gly225, Arg227, Met230, **Arg234**, Tyr240
**5f**	-8.7	Leu74, Gly75, Ala76, Gly77, Asn78, **Gly79**, Val82, **Lys97**, Ile99, Ile141, Ser150, Asp190*, **Lys192**, **Ser194**, Asn195, Leu197, Asp208*, Phe209, Leu215, Met219*, Gly210, Ile216
**6a**	-10.5	**Lys97**, Ile99, Leu115, Leu118*, Val127, Gly128, Phe129, Ile141, Met143*, Arg189, Cys207, Asp208, Phe209, Gly210, Val211, Ser212, Leu215*, Ile216, Met219*, Ala220
**6b**	-9.8	Lys97, Ile99, Leu118*, Gly128, Phe129, Ile141, Met143*, Arg189*, Asp190, Asp208*, Phe209*, Gly210, Val211, Ser212, Leu215, Ile216*, Met219*, Ala220, Gly225, Met230
**6c**	-10.2	Lys97, Leu115, Leu118, Val127, Phe129, Ile141*, Met143, Arg189*, Asp190, Cys207, Asp208, Phe209, Gly210, **Val211**, **Ser212**, Leu215, Ile216*, Met219, Met230
**6e**	-9.9	Lys97, Ile99, Leu115, Leu118, Val127, Phe129, Ile141, Met143, **Arg189**, Asp190, Cys207, Asp208, Phe209, Gly210, Ser212, Val211, Leu215, Ile216, Met219, Ala220, Gly225, Thr226, met230, **Arg234**, Tyr240
		**TPK** (mean ΔGbin = -9.62 kcal/mol)
**5e**	-8.6	Leu881, Gly882,Glu883, **Gly884**, His885, Phe886, Gly887, Lys888, Val889*, **Lys908**, Ser963, Glu966, His918, Asp1003, **Arg1007**, Asn1008, Leu1010, Gly1020, Asp1021*, Gly1023, Leu1024, Asp1039, Asp1042, Pro1044
**5f**	-8.2	Leu881, Gly882, Glu883, Gly884*, His885, Phe886, Gly887, Val889, **Lys908**, Leu910, His918, Asp921, Ser963, Lys965, Asp1003, **Arg1007**, Asn1008, Leu1010, Asp1021, Gly1023, Leu1024, Asp1039, Asp1042
**6a**	-10.3	Leu881*, Gly882, Glu883, Gly884*, His885, Phe886, Gly887, Lys888, Val889*, Ala906*, Lys908*, Leu910, His918, Asp921, Leu922, Met956*, Asp1003, **Arg1007**, Asn1008, Leu1010*, Gly1020, Asp1021*
**6b**	-10.3	Leu881*, Gly882, Glu883, Gly884, His885, Phe886, Gly887, Lys888, Val889*, Ala906*, Lys908*, Leu910, Asp921, Leu922, Glu925, Met956*, Phe958*, Leu959*, Asp1003, **Arg1007**, Asn1008, Leu1010*, Gly1020, Asp1021*, Gly1023
**6c**	-10.4	Leu881*, Gly882, Glu883, Gly884, His885, Phe886, Gly887, Lys888, **Val889***, Ala906*, Ly908*, Met956, Phe958*, Leu959, Asp1003, **Arg1007**, **Asn1008**, Leu1010*, Gly1020, Asp1021*, Gly1023, Leu1024, Asp1042
**6e**	-9.9	Leu881, Gly882, Glu883, Gly884, Phe886, Gly887, Lys888, Val889*, Ala906, Lys908*, Leu910, His918, Asp921, Leu922, Glu925, Phe958, Leu959, Gly962, Ser963, Lys965, Glu966, Asp1003, **Arg1007**, Asn1008, Leu1010*, Asp1021*, Gly1023
		**EGFR** (mean ΔGbin = -9.53 kcal/mol)
**5e**	-7.2	Leu718, Gly719, Ser720, Val726*, Ala743*, Ile744, **Lys745**, Met766*, Val769, Arg776, Leu777*, Leu788, Ile789, Met790*, Gln791, Met793, Gly796, Cys797, Arg841, Leu844*, **Thr854**, Asp855, Phe856, Leu858
**5f**	-7.4	Leu718*, Gly719, Ser720, Gly721, Val726*, Ala743, Ile744, Lys745, Met766*, Cys775, Arg776, Leu777*, Leu788*, Met790*, Gln791, Leu792, Met793, Gly796, Cys797*, Asp800, **Arg841**, Asn842, Leu844*, Asp855, Phe856, Leu858
**6a**	-10.6	Gly719, Ser720, Val726*, Ala743*, Ile744, **Lys745***, Mey766, Cys775, Arg776, Leu777, Leu788, Ile789, Met790*, Met793, Cys797, Arg841, Leu844, Thr854, Asp855, Phe856*, Leu858
**6b**	-10.6	Gly719, Ser720, Val726*, Ala743, Ile744, **Lys745***, Met766, Cys775, Arg776, Leu777, Leu788, Ile789, Met790*, Met793, Cys797 Amino Acids , Asp800, Arg841, Leu844, Thr854, Asp855, Phe856, Leu858
**6c**	-11.1	Val726*, Ala743*, Ile744, **Lys745***, Met766, Cys775, Arg776, Leu777, Leu788, Ile789, Met790*, Cys797, Gly719, Arg841, Leu844 Thr854, Asp855, Phe856, Leu858

**6e**	-10.0	Gly719, Ser720, Gly721, Val726*, Ala743*, Ile744, **Lys745***, Met766, Cys775, Arg776, Leu777, Leu788*, Ile789, Met790*, Gly796, Cys797*, Asp800, Arg841, Asn842, Leu844, **Thr854**, Asp855, Phe856, Leu858

Bolded names correspond to those amino acids involved in H-bonds with the corresponding CBHQs and CNQs.
Residues with * correspond to amino acids that interact with the ligand by any type of Pi-interactions
