# Supplementary material for: 2-Acetyl-5,8-dihydro-6-(4-methyl-3-pentenyl)-1,4-naphthohydroquinone-Derived Chalcones as Potential Anticancer Agents
Source: Molecules. 2023 Oct 19;28(20):7172. doi: 10.3390/molecules28207172 (PMC10609043; doi:10.3390/molecules28207172)
Supplement: Supplementary file 1 [file molecules-28-07172-s001.zip › Table S4.pdf]

Table S4. Physical and Pharmacokinetic data predicted by QikProp[a] extensions for compounds **5e**, **5f**, and **6a-c,e.**

**Compound**	% **HOA**[b]	QP**PCaco**[c]	QP**logBB**[d]	QP**logHERG**[e]	QP**logKhsa**[f]	QP**logS**[g]	**PSA**[h]	Number of violations (<3)
**5e**	90.757	1.454.968	-1.168	**-6.064**	0.665	**-6.773**	113.326	2
**5f**	84.271	661.610	-1.628	**-6.101**	0.766	**-7.281**	115.871	2
**6a**	100.000	592.613	-1.272	**-6.749**	0.416	-5.746	78.898	1
**6b**	100.000	639.622	-1.303	**-6.515**	0.378	-5.749	87.049	1
**6c**	100.000	602.342	-1.283	**-6.565**	0.584	**-6.260**	78.848	2
**6e**	100.000	559.964	-1.444	**-5.799**	0.274	-5.312	101.681	1

a: QikProp (QP) version 6.5; [b] HOA, Human oral absorption (>80% - high and <25% - poor) ; [c] QPPCaco: Predicted apparent Caco-2 cell permeability (<25 nm/sec - poor and >500 nm/sec great); [d] QPlogBB: Predicted log brain/blood partition coefficient (appropriate values between -3.0 and 1.2); [e] QPlogHERG: Predicted IC_{50} value for blockage of HERG K+ channels (above −5); [f] QPlogKhsa: Prediction of binding to human serum albumin (appropriate values between -1.5 and 1.5); [g] QPlogS: Predicted log aqueous solubility (appropriate values above -6.5); [h] PSA: polar surface area (appropriate values between 70–200 Å^2). The intervals of appropriate values are defined by QP. Bold numbers correspond to violations of the Jorgensen Rule-of-Three.
