# Supplementary material for: 2-Acetyl-5,8-dihydro-6-(4-methyl-3-pentenyl)-1,4-naphthohydroquinone-Derived Chalcones as Potential Anticancer Agents
Source: Molecules. 2023 Oct 19;28(20):7172. doi: 10.3390/molecules28207172 (PMC10609043; doi:10.3390/molecules28207172)
Supplement: Supplementary file 1 [file molecules-28-07172-s001.zip › Table S5.pdf]

Table S5. Evaluation parameters of Lipinski's rule of five and its extensions for compounds **5e**, **5f**, and **6a-c,e.**

Compound	mol_MW (<500amu)	donorHB (<5)	accptHB (<10)	QPlogPo/w (<5)	#rotor (0−15)	N of violations (<5)
**5e**	**548.632**	0.000	9.250	**5.656**	12	2
**5f**	**548.632**	0.000	9.250	**5.595**	12	2
**6a**	370.447	0.000	6.000	4.202	7	0
**6b**	400.473	0.000	6.750	4.283	8	0
**6c**	384.474	0.000	6.000	4.519	7	0
**6e**	460.526	0.000	8.250	4.216	10	0

a: QikProp (QP) version 6.5; mol_MW: Molecular weight; donorHB: Number of hydrogen-bond donors (OH, NH, SH); accptHB: Number of hydrogen-bond acceptors (heteroatom O; N; S; F or pairs of transferable electrons); logP: Predicted log *n*-octanol/water partition coefficient; #rotor: Number of non-trivial (not CX3), non-hindered (not alkene, amide, small ring) rotatable bonds. Bold numbers correspond to Lipinski Rule-of Five violations.
